# Direct Catalytic Enantio- and Diastereoselective Ketone Aldol Reactions of Isocyanoacetates**

**DOI:** 10.1002/anie.201411852

**Published:** 2015-03-03

**Authors:** Raquel delaCampa, Irene Ortín, Darren J Dixon

**Affiliations:** a Department of Chemistry, Chemistry Research Laboratory University of Oxford, Mansfield Road, Oxford OX1 3TA (UK)

**Keywords:** aldol reaction, asymmetric catalysis, enantioselectivity, isocyanoacetates, oxazolines

## Abstract

A catalytic asymmetric aldol addition/cyclization reaction of unactivated ketones with isocyanoacetate pronucleophiles has been developed. A quinine-derived aminophosphine precatalyst and silver oxide were found to be an effective binary catalyst system and promoted the reaction to afford chiral oxazolines possessing a fully substituted stereocenter with good diastereoselectivities and excellent enantioselectivities.

The aldol reaction is one of the most powerful methods for the construction of β-hydroxy carbonyl compounds.^1^ The importance of these building blocks, contained in a wide variety of biologically relevant compounds, has promoted the development of several catalytic asymmetric methods for their production.^2^ However, despite enormous progress in the aldol addition arena, its application to the synthesis of tertiary alcohols still remains a major challenge, principally owing to a lack of reactivity and the fact that the differentiation of the enantiotopic faces is more difficult with ketone electrophiles than with the corresponding aldehydes. Furthermore, deleterious side reactions, such as retro-aldol reactions, can predominate when a ketone moiety is involved.^3^ Although a few catalytic asymmetric aldol reactions with unactivated ketones have been reported,^4^ the development of new and efficient catalytic asymmetric methods to access chiral tertiary alcohols remains an important goal in modern asymmetric catalysis.^5^

Along these lines, we recognized that the catalytic asymmetric ketone aldol reaction of isocyanoacetate pronucleophiles^6^ could be a synthetically powerful approach. Isocyanoacetate ester addition reactions to carbonyl^7^ or imine electrophiles^8^, ^9^ directly afford the respective oxazoline or imidazoline heterocycles, which can be ring-opened under mild hydrolytic conditions to yield β-substituted α-amino acids. Although the catalytic asymmetric version of this reaction has been widely studied using aldehydes,^7^ to date, no enantioselective example using unactivated ketones has been reported despite its potential to provide an elegant asymmetric route to α-amino acid derivatives possessing a chiral tertiary alcohol in the β-position (Scheme1).^10^ In a related study, the asymmetric aldol addition reaction of isothiocyanato esters and unactivated ketones, which afforded oxazolidinethione products with a fully substituted β-stereocenter, was described.^11^

**scheme 1:**
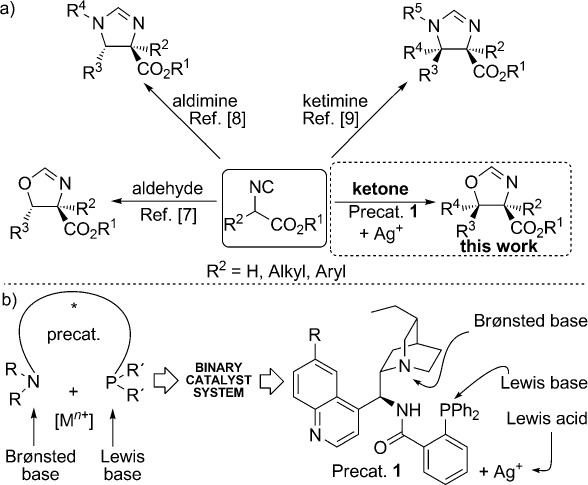
a)Catalytic asymmetric addition reactions of isocyanoacetate pronucleophiles to carbonyl or imine electrophiles. b)Aminophosphine/silver(I) binary catalyst system applied in this work.

For promoting and controlling various addition reactions, our group has developed an effective binary catalyst system comprising a “soft” metal ion, such as a silver (I) ion, and a cinchona-derived aminophosphine precatalyst of type **1**. This system promotes the highly diastereo- and enantioselective aldol reaction of isocyanoacetates with aldehydes, 7l and Mannich reactions of aldimines8e and ketimines.9a The precatalyst is equipped with Brønsted basic and Lewis basic sites and also possesses a hydrogen-bond donor group located in the proximity of the chiral pocket that is created by the cinchona scaffold (Scheme1). In conjunction with Ag^I^ ions, these features provide remarkable catalytic activity in reactions of isocyanoacetate pronucleophiles and accordingly prompted us to address the challenging enantioselective aldol reaction of unactivated ketones.

Initially, the reaction of acetophenone (**3 a**) and *tert*-butyl isocyanoacetate (**2 a**) in EtOAc at −20 °C was selected as a model system, and the performance of our previously described silver oxide/aminophosphine catalytic system, which was employed in a 2:1 molar ratio of aminophosphine precatalyst to metal, was assessed. 7l Pleasingly, using cinchonidine-derived aminophosphine **1 c**, *trans*-oxazoline (4*R*,5*S*)-**4 a** was obtained as the major product with significant diastereo- and enantiocontrol (Table1, entry1; 89:11 d.r., 90:10 e.r.). With quinine-derived precatalyst **1 a**, product **4 a** was afforded in higher diastereo- and enantioselectivity (entry2; 95:5 d.r., 94:6 e.r.), whereas pseudoenantiomeric **1 b** yielded the enantiomeric product (4*S*,5*R*)-**4 a** as a 90:10 mixture of diastereomers with 90:10 e.r. (entry3). Alternative silver sources were also tested in conjunction with precatalyst **1 a** (entries4 and 5) in the hope that an increase in the selectivity would be observed. Although the use of silver carbonate afforded *trans*-oxazoline product (4*R*,5*S*)-**4 a** with high enantioselectivity (entry4; 91:9 d.r., 94:6 e.r.), **4 a** was formed with enhanced diastereocontrol and yield when silver(I) oxide was employed (see entry2). In terms of diastereoselectivity, EtOAc was found to be the best solvent compared with TBME, CH_2_Cl_2_, or *i*PrOAc (entries2 and 6–8). Changing the temperature of the reaction to −30 °C or to 0 °C from −20 °C made no improvement to the enantioselectivity (entries9 and 10). Finally, reactions performed in the absence of Ag_2_O or without precatalyst **1 a** gave no product after five days at −20 °C (entries11 and 12). These results demonstrate that the active catalyst is made from a combination of aminophosphine precatalyst **1 a** and a Ag^I^ salt.

**Table 1:** Optimization studies. 
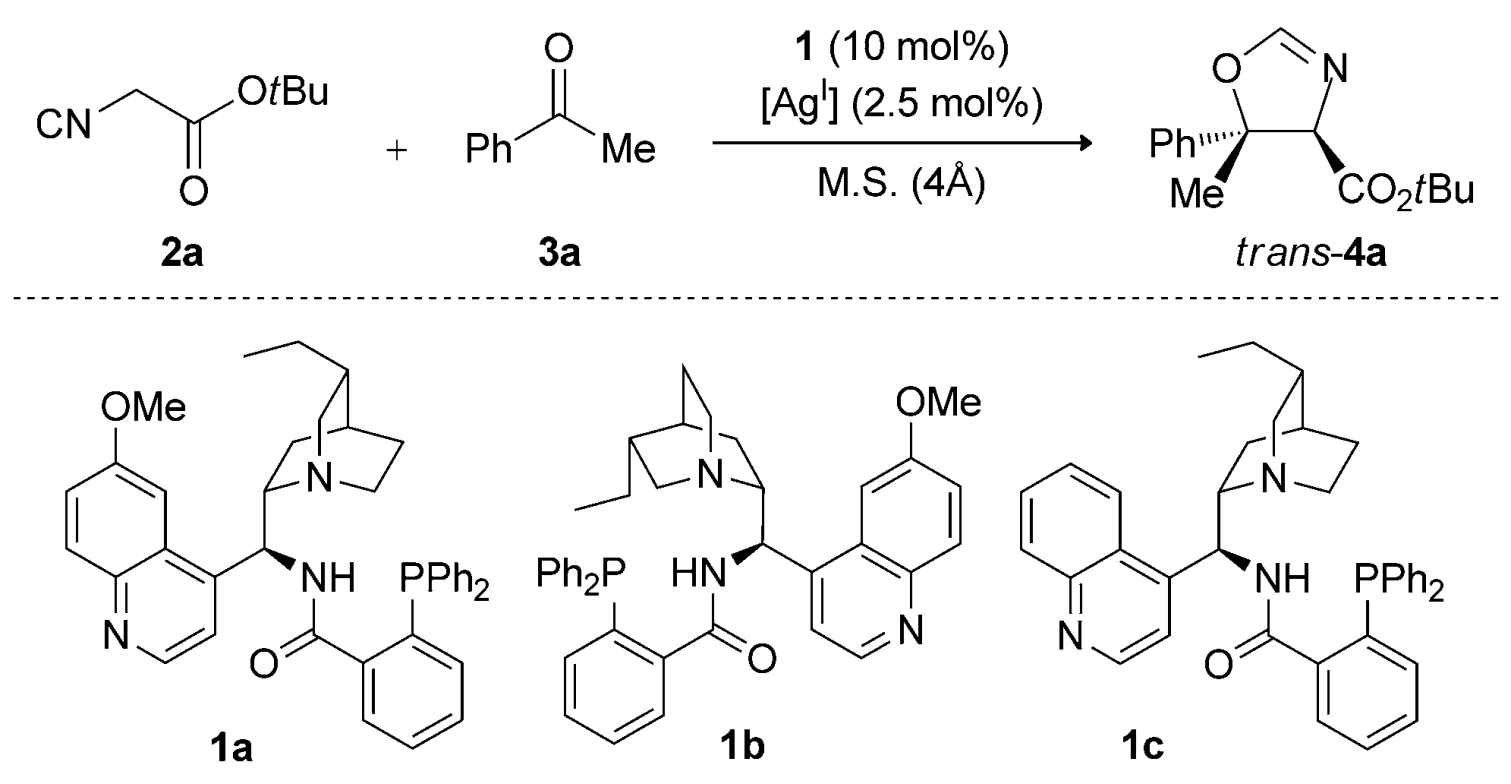

Entry	1	[Ag]	*T*[°C]	Solvent	*t*[h]	Yield[a] [%]	d.r.[b]	e.r.[c]
1	**1 c**	Ag_2_O	−20	EtOAc	60	78	89:11	90:10
2	**1 a**	Ag_2_O	−20	EtOAc	60	93	95:5	94:6
3	**1 b**	Ag_2_O	−20	EtOAc	60	82	90:10	90:10[d]
4	**1 a**	Ag_2_CO_3_	−20	EtOAc	24	64	91:9	94:6
5	**1 a**	AgOAc	−20	EtOAc	48	55	88:12	91:9
6	**1 a**	Ag_2_O	−20	TBME	36	74	88:12	94:6
7	**1 a**	Ag_2_O	−20	CH_2_Cl_2_	36	63	79:12	85:15
8	**1 a**	Ag_2_O	−20	*i*PrOAc	36	79	89:11	93:7
9	**1 a**	Ag_2_O	−30	EtOAc	96	84	90:10	94:6
10	**1 a**	Ag_2_O	0	EtOAc	48	84	89:11	92:8
11	**1 a**	–	−20	EtOAc	120	0	–	–
12	–	Ag_2_O	−20	EtOAc	120	0	–	–

[a] Combined yield of both diastereomers after flash column chromatography.

[b] The diastereomeric ratio (d.r.) is given as the *trans*/*cis* ratio and was determined by ^1^HNMR analysis of the crude reaction mixture.

[c] The enantiomeric ratios (e.r.) were determined by HPLC analysis on a chiral stationary phase.

[d] Enantiomeric (*4S*,*5R*)-**4 a** was obtained. M.S.=molecular sieves, TMBE=*tert*-butyl methyl ether.

Subsequently, the effect of lowering the catalyst loading was studied (Table2). *trans*-Oxazoline **4 a** was obtained with marginally lower levels of enantioselectivity when the loading was reduced to 5 and 1mol % of **1 a** while maintaining a precatalyst/metal ratio of 2:1 ratio (entries2 and 3). However, diastereo- and enantioselectivities that are comparable to the best values reported in Table1 were achieved when precatalyst **1 a** and Ag^I^ were employed in a 1:1 ratio at a precatalyst loading of 5mol % (entry1).

**Table 2:** Variations of the catalyst loading. 
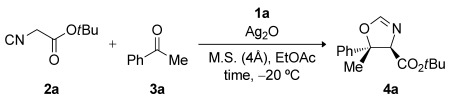

Entry	1 a(mol %)	Ag_2_O (mol %)	*t*[h]	Yield[a] [%]	d.r.[b]	e.r.[c]
1	5	2.5	72	84	94:6	94:6
2	5	1.25	96	85	90:10	93:7
3	1	0.25	96	73	86:14	90:10

[a] Combined yield of both diastereomers after flash column chromatography.

[b] Given as the *trans*/*cis* ratios and determined by ^1^HNMR analysis of the crude reaction mixture.

[c] Determined by HPLC analysis on a chiral stationary phase.

With the optimized conditions established, we proceeded to study the scope of the reaction of *tert*-butyl isocyanoacetate (**2 a**) with different alkyl aryl ketones in the presence of quinine-derived aminophosphine precatalyst **1 a** and Ag_2_O (Table3). Several substituted methyl aryl ketones with either electron-withdrawing or -donating groups afforded the *trans*-configured oxazolines **4 b**–**4 f** as the major products with good diastereoselectivities and very good enantioselectivities (Table3, entries2–6; 93:7–96:4 e.r.). Pleasingly, methyl ketone substrates **3 i** and **3 j**, which feature five- and six-membered heteroaromatic rings, were also well tolerated giving products **4 i** and **4 j** with good stereoselectivities (entries9 and 10). An important success was observed when aryl ethyl ketones **3 k**–**3 n** were used in the reaction. *trans*-Oxazolines **4 k**–**4 n** were obtained as the major products with good diastereoselectivities and excellent enantioselectivities (entries11-14; 98:2–99:1 e.r.). Aryl propyl ketones **3 o**–**3 p** were also excellent substrates and afforded the *trans*-configured oxazoline products **4 o**–**4 p** in high yields and in good to excellent enantioselectivities (entries15 and 16). Finally, isovalerophenone **3 q** afforded *trans*-oxazoline **4 q** in 75 % yield, 96:4 d.r., and 97:3 e.r. (entry17), demonstrating the broad scope of the reaction with respect to different non-activated ketones. We also studied the scope of the reaction with different isocyanoacetates (entries18–20). With ethyl isocyanoacetate (**2 b**) or methyl isocyanoacetate (**2 c**), the *trans*-configured oxazoline products **4 r**–**4 t** were obtained with very good diastereoselectivities and excellent enantioselectivities. Unfortunately, under the optimized conditions, symmetric and unsymmetric aliphatic ketones afforded the corresponding oxazoline products with poor enantioselectivities.^12^

**Table 3:** Scope of the ketone aldol/cyclization reaction with isocyanoacetates. 
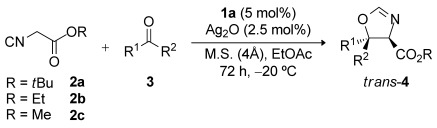

Entry	2	3	R^1^	R^2^	4	Yield [%][a]	d.r.[b]	e.r.[c]
1	**2 a**	**3 a**	Ph	Me	**4 a**	84	94:6	94:6
2	**2 a**	**3 b**	*p*-CH_3_OC_6_H_4_	Me	**4 b**	73	92:8	94:6
3	**2 a**	**3 c**	5*-*Br-thiophen-2-yl	Me	**4 c**	78	88:12	93:7
4	**2 a**	**3 d**	*p-*NO_2_C_6_H_4_	Me	**4 d**	60	90:10	95:5
5	**2 a**	**3 e**	*p-*BrC_6_H_4_	Me	**4 e**	71	89:11	95:5
6	**2 a**	**3 f**	*p-*CNC_6_H_4_	Me	**4 f**	80	90:10	96:4
7	**2 a**	**3 g**	4-F,3*-*BrC_6_H_3_	Me	**4 g**	83	85:15	89:11
8	**2 a**	**3 h**	3,5*-*(CF_3_)_2_C_6_H_3_	Me	**4 h**	82	86:14	88:12
9	**2 a**	**3 i**	5-methylthiazol-2-yl	Me	**4 i**	55	91:9	93:7
10	**2 a**	**3 j**	pyrazin-2-yl	Me	**4 j**	75	91:9	91:9
11	**2 a**	**3 k**	Ph	Et	**4 k**	76	90:10	99:1
12	**2 a**	**3 l**	*p-*MeC_6_H_4_	Et	**4 l**	81	91:9	98:2
13	**2 a**	**3 m**	*p-*FC_6_H_4_	Et	**4 m**	83	88:12	99:1
14	**2 a**	**3 n**	*p-*BrC_6_H_4_	Et	**4 n**	73	90:10	99:1
15	**2 a**	**3 o**	Ph	Pr	**4 o**	79	87:13	99:1
16	**2 a**	**3 p**	2-thienyl	Pr	**4 p**	81	84:16	96:4
17	**2 a**	**3 q**	Ph	CH_2_*i*Pr	**4 q**	75	96:4	97:3
18	**2 b**	**3 k**	Ph	Et	**4 r**	81	90:10	99:1
19	**2 b**	**3 l**	*p-*MeC_6_H_4_	Et	**4 s**	82	91:9	98:2
20	**2 c**	**3 k**	Ph	Et	**4 t**	77	91:9	99:1

[a] Combined yield of both diastereomers after flash column chromatography.

[b] Determined by ^1^HNMR analysis of the crude reaction mixture.

[c] Determined by HPLC analysis on a chiral stationary phase.

As the stereochemical outcome favored the production of the *trans*-configured oxazoline product, and as alkyl groups larger than a methyl group were well tolerated in the ketone aldol reaction, our reaction could be applied to the synthesis of oxazoline-fused γ- and δ-lactam derivatives. Therefore, azides **3 r** and **3 s** were subjected to the standard reaction conditions, and pleasingly, oxazolines **4 u** and **4 v** were afforded in good yield and with excellent enantioselectivities. Subsequently, under standard Staudinger conditions, these oxazolines were transformed into the target lactam products **5 a** and **5 b** with high yields in a straightforward manner without compromising stereochemical integrity (Scheme2).

**scheme 2:**
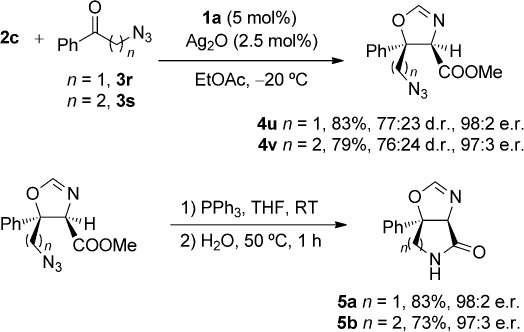
Application of the ketone aldol reaction to the formation of fused bicyclic lactams.

To demonstrate further synthetic utility whilst expanding our knowledge on the hydrolytic manipulation of oxazoline heterocycles,^13^ we subsequently explored the conversion of oxazolines **4** into the corresponding amino acid derivatives under different reaction conditions. The hydrolysis of oxazoline **4 e** using catalytic amounts of HCl afforded the corresponding *N*-formyl derivative **6** in quantitative yield (Scheme3 a). Similarly, methanolysis of **4 e** led to the corresponding β-hydroxy-α-amino acid *tert*-butyl ester **7** under mild conditions (Scheme3 b). These transformations highlight the ability of our method to afford protected serine derivatives with a doubly substituted β-position. Furthermore, treatment of **7** with thiophosgene under basic conditions furnished crystalline oxazolidinethione **8** (Scheme3 c);^14^ its absolute and relative stereochemical configurations were determined by single-crystal X-ray diffraction, and those of the other oxazolines (**4 a**–**4 v**) were assigned by analogy.

**scheme 3:**
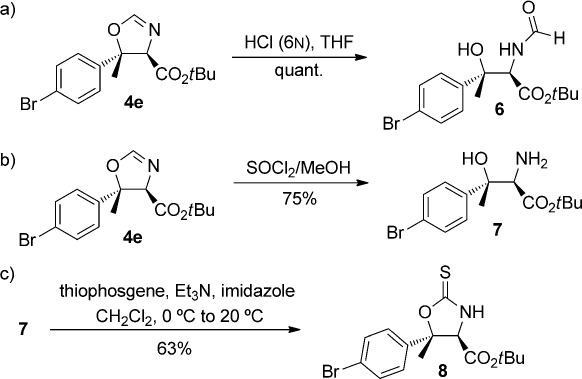
Synthetic manipulations of the oxazoline products.

Based on previous reports9b, 15 and on the known absolute stereochemical configuration of oxazoline products **4**, a transition-state model rationalizing the stereochemical outcome of the ketone aldol reaction between **3 a** and **2 a** in the presence of precatalyst **1 a** and silver oxide is proposed in Scheme4. In the enantiodetermining carbon–carbon bond-forming step, the phosphorus and amide nitrogen atoms of **1 a**, the oxygen atom of ketone **3 a**, and the terminal carbon atom of the isonitrile coordinate to a silver(I) ion in a tetrahedral arrangement. Additional transition-state stabilization is provided through hydrogen bonding of the protonated quinuclidine to the coordinated ketone oxygen atom. Importantly, this interaction creates a well-defined chiral pocket that can readily differentiate the enantiotopic faces of the bound ketone; unfavorable steric interactions force the aryl group away from the quinuclidine, and attack of the enolate occurs preferentially to the *Re* face.

**scheme 4:**
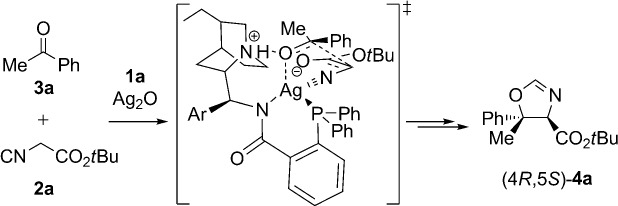
Proposed transition-state model rationalizing the stereochemical outcome of the reaction of 3 a and 2 a in the presence of 1 a and Ag_2_O.

In conclusion, we have developed the first highly enantio- and diastereoselective aldol addition/cyclization reaction of isocyanoacetate esters with unactivated prochiral ketones to afford functionalized oxazolines with a fully substituted stereogenic center at the β-carbon atom. The reaction is efficient and broad in scope and effectively promoted by a binary catalyst system that consists of a cinchona-derived aminophosphine precatalyst and silver oxide. In combination with hydrolytic transformations of the oxazoline heterocycles, this method enables the transformation of simple ketones into the corresponding amino acid derivatives possessing a tertiary alcohol in the β-position. Further studies towards the discovery and application of new asymmetric isocyanoacetate addition reactions are ongoing in our laboratories, and the results will be reported in due course.

## Supporting Information

As a service to our authors and readers, this journal provides supporting information supplied by the authors. Such materials are peer reviewed and may be re-organized for online delivery, but are not copy-edited or typeset. Technical support issues arising from supporting information (other than missing files) should be addressed to the authors.

**miscellaneous_information**
