# Supplementary material for: Direct Catalytic Enantio- and Diastereoselective Ketone Aldol Reactions of Isocyanoacetates
Source: Angew Chem Int Ed Engl. 2015 Mar 3;54(16):4895–8. doi: 10.1002/anie.201411852 (PMC4678506; doi:10.1002/anie.201411852)
Supplement: Supplementary file 1 [file anie0054-4895-sd1.pdf]

Angewandte Chemie
Eine Zeitschrift der Gesellschaft Deutscher Chemiker

Supporting Information

Direct Catalytic Enantio- and Diastereoselective Ketone Aldol Reactions of Isocyanoacetates**

*Raquel de la Campa, Irene Ortín, and Darren J. Dixon**

anie_201411852_sm_miscellaneous_information.pdf

Table of Contents

1 General Experimental
1.1 Solvents and Reagents 4
1.2 Chromatography 4
1.3 Melting points 4
1.4 Mass spectra 4
1.5 Infrared spectra 4
1.6 NMR spectra 4
1.7 Determination of enantiomeric excess 4
1.8 Optical rotations 5
2 Synthesis and Characterization details 5
2.1 Synthesis of starting materials 5
2.1.1 Synthesis and characterization of ligand **1a** 5
2.1.2 Synthesis and characterization of ligand **1b** 6
2.2 General procedure for the synthesis of oxazolines **4** 7
2.2.1 Compound **4a** 7
2.2.2 Compound **4b** 8
2.2.3 Compound **4c** 8
2.2.4 Compound **4d** 8
2.2.5 Compound **4e** 9
2.2.6 Compound **4f** 9
2.2.7 Compound **4g** 9
2.2.8 Compound **4h** 10
2.2.9 Compound **4i** 10
2.2.10 Compound **4j** 10
2.2.11 Compound **4k** 11
2.2.12 Compound **4l** 11
2.2.13 Compound **4m** 11
2.2.14 Compound **4n** 12
2.2.15 Compound **4o** 12
2.2.16 Compound **4p** 12
2.2.17 Compound **4q** 13
2.2.18 Compound **4r** 13
2.2.19 Compound **4s** 13
2.2.20 Compound **4t** 13
2.2.21 Compound **4u** 14
2.2.22 Compound **4v** 14
2.3 General procedure for the synthesis of racemic oxazolines 15

2.4 Synthesis and characterization of compounds **5** 15
2.5 Synthesis and characterization of compound **6** 16
2.6 Synthesis and characterization of compound **7** 16
2.7 Synthesis and characterization of compound **8** 17
3. ^{1}H-NMR and ^{13}C-NMR spectra of pre-catalysts 18
3.1 ^{1}H-NMR and ^{13}C-NMR spectra of compound **1a** 18
3.2 ^{1}H-NMR and ^{13}C-NMR spectra of compound **1b** 20
4. ^{1}H-NMR and ^{13}C-NMR and HPLC traces of oxazolines and derivatives 21
4.1 ^{1}H-NMR and ^{13}C-NMR spectra and HPLC traces of compound **4a** 21
4.2 ^{1}H-NMR and ^{13}C-NMR spectra and HPLC traces of compound **4b** 23
4.3 ^{1}H-NMR and ^{13}C-NMR spectra and HPLC traces of compound **4c** 25
4.4 ^{1}H-NMR and ^{13}C-NMR spectra and HPLC traces of compound **4d** 27
4.5 ^{1}H-NMR and ^{13}C-NMR spectra and HPLC traces of compound **4e** 29
4.6 ^{1}H-NMR and ^{13}C-NMR spectra and HPLC traces of compound **4f** 31
4.7 ^{1}H-NMR and ^{13}C-NMR spectra and HPLC traces of compound **4g** 33
4.8 ^{1}H-NMR and ^{13}C-NMR spectra and HPLC traces of compound **4h** 35
4.9 ^{1}H-NMR and ^{13}C-NMR spectra and HPLC traces of compound **4i** 37
4.10 ^{1}H-NMR and ^{13}C-NMR spectra and HPLC traces of compound **4j** 39
4.11 ^{1}H-NMR and ^{13}C-NMR spectra and HPLC traces of compound **4k** 41
4.12 ^{1}H-NMR and ^{13}C-NMR spectra and HPLC traces of compound **4l** 43
4.13 ^{1}H-NMR and ^{13}C-NMR spectra and HPLC traces of compound **4m** 45
4.14 ^{1}H-NMR and ^{13}C-NMR spectra and HPLC traces of compound **4n** 47
4.15 ^{1}H-NMR and ^{13}C-NMR spectra and HPLC traces of compound **4o** 49
4.16 ^{1}H-NMR and ^{13}C-NMR spectra and HPLC traces of compound **4p** 52
4.17 ^{1}H-NMR and ^{13}C-NMR spectra and HPLC traces of compound **4q** 54
4.18 ^{1}H-NMR and ^{13}C-NMR spectra and HPLC traces of compound **4r** 56
4.19 ^{1}H-NMR and ^{13}C-NMR spectra and HPLC traces of compound **4s** 58
4.20 ^{1}H-NMR and ^{13}C-NMR spectra and HPLC traces of compound **4t** 60
4.21 ^{1}H-NMR and ^{13}C-NMR spectra and HPLC traces of compound **4u** 62
4.22 ^{1}H-NMR and ^{13}C-NMR spectra and HPLC traces of compound **4v** 64
4.23 ^{1}H-NMR and ^{13}C-NMR spectra and HPLC traces of compound **5a** 66
4.24 ^{1}H-NMR and ^{13}C-NMR spectra and HPLC traces of compound **5b** 68
4.25 ^{1}H-NMR and ^{13}C-NMR spectra and HPLC traces of compound **6** 70
4.26 ^{1}H-NMR and ^{13}C-NMR spectra of compound **7** 72
4.27 ^{1}H-NMR and ^{13}C-NMR spectra and HPLC traces of compound **8** 73
5. X-Ray data of compound **8** 75
6. ^{1}H-NMR and ^{13}C-NMR spectra and HPLC traces of **4x**, **4y** and **4z** 75

1 General Experimental

1.1 Solvents and Reagents

All reagents bought from commercial supliers were used as sold. Organic solvents were evaporated under reduced pressure using a Büchi rotary evaporator. Syringes and needles were oven dried at 90 ºC. Anhydrous dichloromethane and tetrahydrofuran were dried by filtration through activated alumina (powder ≈ 159 mesh, pore size 58 A, basic, Sigma aldrich) columns.

1.2 Chromatography

Reactions were monitored by thin layer chromatography (TLC) using Merck silica gel 60 F254 plates and visualized by fluorescence quenching under UV light. In addition, TLC plates were stained with a dipping solution of vanillin (15 g vanillin + 250 mL ethanol + 2.5 mL conc. H_2SO_4). Chromatographic purification was performed on VWR 60 silica gel 40-60 μm using HPLC grade solvents that were used as supplied.

1.3 Melting points

Melting points were obtained on a Leica Galen III Hot-stage melting points apparatus and microscope and are uncorrected.

1.4 Mass spectra

High-resolution mass spectra (HRMS) were recorded on Bruker Daltonics MircroTOF mass spectrometer. High-resolution mass spectra (EI) were recorded on a Bruker FT-ICR Apex III mass spectrometer.

1.5 Infrared spectra

Infrared spectra were recorded on a Bruker Tensor 27 FT-IR spectrometer as a thin film. Only selected maximum absorbances are reported.

1.6 NMR spectra

NMR spectra were recorded using a Bruker Avance 200 MHz or 400 MHz spectrometer, chemical shifts (δ) are quoted per million referenced to the residual solvent peak. The multiplicity of each signal is designated using the following abbreviation: s, singlet; d, doublet; t, triplet; q, quartet; m, multiplet. Coupling constants (*J*) are reported in Hertz (HZ).

1.7 Determination of enantiomeric excess

Enantiomeric excess were determined using analytical high performance liquid chromatography (HPLC) performed on an Agilent Technologies 1200 Series or 1260 Infinity Series systems (column and solvent conditions are given with the compound).

1.8 Optical rotations

Optical rotations were recorded using Perkin-Elmer 241 polarimeter; specific rotation (SR) ($[\alpha]_D$) are reported in 10^{-1} deg.cm^2.g^{-1}; concentrations (*c*), are quoted in g/100mL; *D* refers to the D-line of sodium (589 nm); temperatures (*T*) are given in degree Celsius (ºC).

2 Synthesis and Characterizacion details

2.1 Starting Materials

Compounds **2a**[1], **3r**[2], **3s**[3] and pre-catalyst **1c**[4] were prepared according to literature procedures. Compounds **2b**, **2c** were acquired commercially.

2.1.1 Synthesis and characterization of 2-(diphenylphosphino)-*N*-((*S*)-((1*S*,2*S*,4*S*,5*R*)-5-ethylquinuclidin-2-yl)(6-methoxyquinolin-4-yl)methyl)benzamide 1a

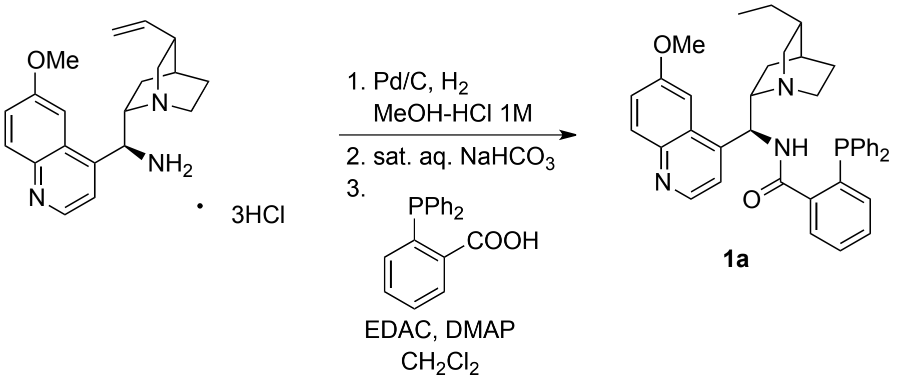

9-amino-9-deoxyepiquinine triple hydrochloride salt (6.2 g, 14.5 mmol, 1.0 equiv) was dissolved in 75 mL of a mixture of 2:1 MeOH/HCl_{aq} 1M and 10% Pd/C (939 mg) was added. The reaction mixture was stired under hydrogen atmosphere for 12 h at room temperature and then filtered through Celite washing with water and MeOH. The filtrate was concentrated under reduced pressure and neutralized with sat. $NaHCO_3$. The resultant solution was extracted several times with CH_2Cl_2. The combined CH_2Cl_2 extracts were dried over Na_2SO_4 and concentrated under reduced pressure to yield a colourless oil (3.5 g of the reduced free amine, 10.5 mmol) which was dissolved in 300 mL of dry CH_2Cl_2. 2-(diphenylphosphino)benzoic acid (3.88g, 12.7 mmol, 1.2 equiv), 4-dimethylaminopyridine (155 mg, 1.2 mmol, 0.12 equiv) were added. The mixture was cooled to 0 ºC, N-(3-Dimethylaminopropyl)-N-ethylcarbodiimide hydrochloride (2.2 g, 11.6 mmol, 1.1 equiv) was added and the ice bath was removed and the resultant solution was stirred at room temperature for 12 h. CH_2Cl_2 was then removed under reduced pressure and the residue was dissolved in EtOAc

[1] N. Elders, Rob F. Schmitz, Frans J. J. de Kanter, Eelco Ruijter, Marinus B. Groen, and Romano V. A. Orru, *J. Org. Chem.* **2007**, *72*, 6135-6142.

[2] A. Cuetos, F. R. Bisogno, I. Lavandera, V. Gotor, *Chem. Commun* **2013**, *49*, 2625-2627.

[3] P. N. D. Singh, S. Muthukrishnan, R. S. Murthy, R. F. Klima, S. M. Mendel, M. Hawk, N. Yarbrough, A. D. Gudmundsdottir *Tetrahedron Lett.* **2003**, *44*, 9169-9171.

[4] F. Sladojevich, A. Trabocchi, A. Guarna, D. J. Dixon, *J. Am. Chem. Soc.* **2011**, *133*, 1710-1713.

and washed with water, 10% $NaHCO_3$, brine and dried over Na_2SO_4. The organic phase was concentrated under reduced pressure and purified by flash column chromatography (EtOAc/MeOH 9:1). Impure fractions were purified by flash column chromatography (EtOAc/Et_3N 99:1 to 95:5) to yield 3.24 g (50%) of **1a**. $[\alpha]_D^{20}$: -15.5 (c 0.4, $CHCl_3$); M.p.: 104-105 °C; ^{1}H-NMR ($CDCl_3$, 500 MHz): δ 8.70 (1H, d, J = 5.0 Hz), 8.05 (1H, d, J = 10.0 Hz), 7.69-7.73 (m, 2H), 7.40-7.43 (m, 2H), 7.28-7.35 (m, 8H), 7.17-7.24 (m, 4H), 6.94-6.97 (m, 1H), 5.46 (br s, 1H), 4.02 (s, 3H), 3.12-3.174 (m, 2H), 3.01 (br s, 1H), 2.61-2.67 (m, 1H), 2.29-2.31 (m, 1H), 1.60-1.65 (m, 1H), 1.34-1.55 (m, 4H), 1.20-1.33 (m, 3H), 0.92-0.94 (m, 1H), 0.85 (t, J = 5.0 Hz, 3H); ^{13}C-NMR ($CDCl_3$, 125 MHz, P-C coupling not removed): δ 12.0, 14.2, 15.3, 21.1, 25.2, 25.9, 27.4, 28.5, 37.2, 41.1, 55.7, 57.6, 60.4, 65.9, 102.3, 121.6, 128.4, 128.5, 128.6, 128.7, 128.8, 130.2, 131.6, 133.5, 133.6, 133.7, 133.8, 134.3, 135.5, 136.9, 137.0, 137.3, 137.4, 141.4, 141.6, 144.7, 157.7, 168.9; ^{31}P-NMR ($CDCl_3$, 162 MHz): δ −10.4; IR: ν_{max}/cm^{-1} 2930, 1646, 1509, 1240, 746; HRMS (ESI, MeOH) calcd $C_{39}H_{41}N_3O_2P$ (M+H$^+$) 614.2931, found 614.2925

2.1.2 Synthesis and characterization of 2-(diphenylphosphino)-*N*-((1*R*)-((2*S*,5*R*)-5-ethylquinuclidin-2-yl)(6-methoxyquinolin-4-yl)methyl)benzamide 1b

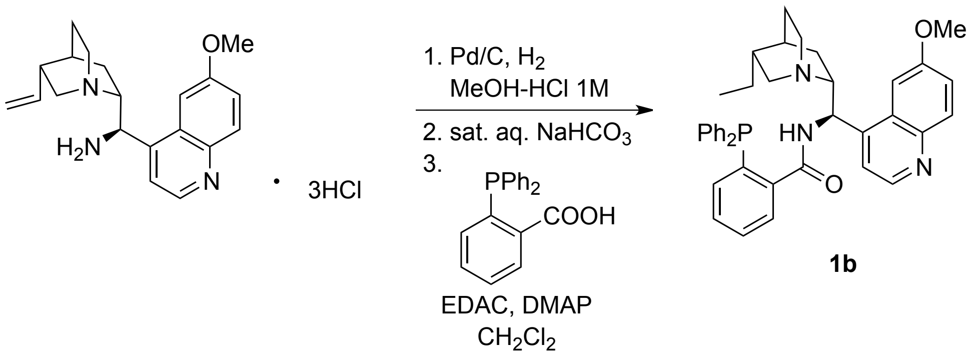

9-amino-9-deoxyepiquinidine triple hydrochloride salt (3.00 g, 6.93 mmol, 1 equiv) was dissolved in a 2:1 mixture MeOH/ 1M HCl (45 mL) and 10% (w/w) Pd/C (369 mg, 0.346 mmol, 0.05 equiv) was added. The reaction mixture was stirred under hydrogen atmosphere (balloon) at room temperature for 7 h, then filtered through Celite washing with MeOH. Evaporation of the filtrate gave a yellow solid, which was taken up with 0.5 M KOH (80 mL) and extracted with a 3:1 mixture of $CHCl_3$/*i*-PrOH (8 times). The combined organic layers were dried over Na_2SO_4, filtered and evaporated to afford a thick orange oil (2.17g of the reduced free amine, 6.65 mmol, 96%). The crude was dissolved in dry CH_2Cl_2 (150 mL) and 2-(diphenylphosphino)benzoic acid (2.20 g, 6.98 mmol, 1.05 equiv) and 4-dimethylaminopyridine (81.0 mg, 0.665 mmol, 0.1 equiv) were added in sequence. After cooling the solution to 0 ºC, *N*-(3-Dimethylaminopropyl)-N-ethylcarbodiimide hydrochloride (1.37 g, 6.98 mmol, 1.05 equiv) was added. The reaction mixture was stirred at room temperature for 13 h, then the solvent was evaporated. The residual orange foam was taken up in EtOAc and washed with water, 10% $NaHCO_3$, brine. The organic phase was dried over Na_2SO_4 and evaporated. The crude was purified by flash column chromatography (first column: EtOAc to EtOAc/MeOH 9:1; second column EtOAc/Et_3N 99:1 to 95:5) to yield 2.04 g (50%) of **1b** as a fluffy white solid. $[\alpha]_D^{20}$: +89.8 (*c* 0.500, $CHCl_3$); M.p.: 103-105 °C; ^{1}H-NMR ($CDCl_3$, 500 MHz): δ 8.59 (1H, d, J = 5.0 Hz), 7.99 (1H, d, J = 9.0 Hz), 7.65 (1H, br s), 7.61 (1H, d, J = 2.5 Hz), 7.47-7.33

(3H, m), 7.30-7.26 (8 H, m), 7.21 (2H, dt, J = 7.5, 1.5 Hz), 7.17 (2H, t, J = 7.5 Hz), 6.91 (1H, m), 5.30 (1H, br s), 3.97 (3H, s), 2.90-2.65 (4H, m), 2.50 (1H, m), 1.76 (1H, br s), 1.51 (1H, br s), 1.45-1.35 (5H, m), 1.22 (1H, m), 0.92 (1H, m), 0.88 (3H, t, J = 7.0 Hz); ^{13}C-NMR ($CDCl_3$, 125 MHz, P-C coupling not removed): δ 169.1, 157.2, 147.3, 144.8, 141.6, 141.4, 137.6, 137.5, 137.2, 137.1, 135.8, 135.7, 134.4, 134.1, 133.9, 133.8, 133.7, 131.8, 130.3, 128.9, 128.9, 128.7, 128.7, 128.6, 128.6, 121.9, 101.8, 55.6 , 49.3, 37.6, 27.6, 26.2, 25.8, 25.2, 12.2; ^{31}P-NMR ($CDCl_3$, 162 MHz): δ −10.4; IR: ν_{max}/cm^{-1} 2933, 1621, 1433, 1227, 1151, 744,; HRMS (ESI, MeOH) calcd $C_{39}H_{41}N_3O_2P$ ($M+H^+$) 614.2931, found 614.2940

2.2 General procedure for the preparation of oxazolines 4

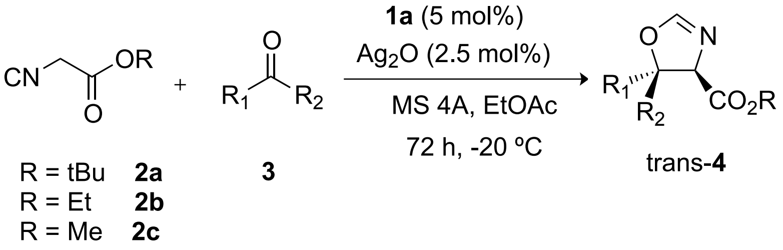

Pre-catalyst **1a** (11.8 mg, 0.017 mmol) and Ag_2O (2.1 mg, 0.008 mmol) were dissolved in 2.0 mL of EtOAc in presence of powdered 4A MS and the ketone **3** (0.386 mmol, 1.1 equiv) was added. The heterogeneous mixture was cooled at -20 ºC in a fridge and stirred for 30 min. After that, the isocyanate **2** (0.351 mmol, 1.0 equiv) previously dissolved in 2.0 mL of EtOAc and cooled at -20 ºC, was added. The reaction mixture was stirred at the same temperature for 72 h (time needed to the total consumption of the isocyanacetate, according with the TLC). The reaction mixture was then quickly filtered through a short pad of celite (in a glass pipette) and eluted with EtOAc. The filtrate was concentrated under reduced pressure and purified by flash column chromatography on silica gel using mixtures of petroleum ether/ethyl acetate (9/1→3/1→1/1) to yield oxazolines **4** as pure compounds.

2.2.1 Synthesis and characterization of (4*S*,5*S*)-*tert*-butyl 5-methyl-5-phenyl-4,5-dihydrooxazole-4-carboxylate 4a. The general procedure was followed. The desired product was obtained as a colorless oil in 84% yield (77 mg, trans:cis=94:6; data for the trans diastereoisomer).The er was determined by HPLC using a Chiralpack AD-H [hexane/iso-propanol 90:10, λ 220, 1 mL/min] t (minor) = 6.95 min., t (major) = 7.58 min (94:6). $[\alpha]_D^{20}$ = -72.6 (*c* 1.00, $CHCl_3$); ^{1}H NMR (400 MHz, C_6D_6) δ 1.31 (s, 9H), 1.62 (s, 3H), 4.81 (d, J = 2.0 Hz, 1H), 6.64 (d, J = 2.0 Hz, 1H), 7.02-7.08 (m, 1H), 7.13-7.17 (m, 2H), 7.42-7.48 (m, 2H); ^{13}C NMR (100 MHz, C_6D_6) δ 24.7 (CH_3), 27.5 (3xCH_3), 78.5 (CH), 81.3 (C), 87.3 (C), 124.1 (2xCH), 127.5 (CH), 128.6 (2xCH), 146.1 (C), 154.8 (C), 168.3 (C); IR ν_{max}/cm^{-1} 1742, 1633, 1369, 1150, 700; HRMS (ES) calcd $C_{15}H_{20}NO_3$ $[M+H]^+$ 262.1438, found 262.1444.

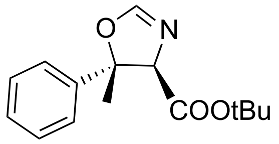

2.2.2 Synthesis and characterization of (4*S*,5*S*)-*tert*-butyl 5-(4-methoxyphenyl)-5-methyl-4,5-dihydrooxazole-4-carboxylate 4b. The general procedure was followed. The desired product was

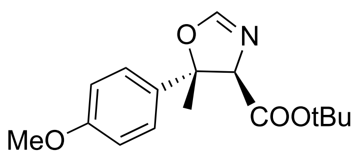

obtained as a colorless oil in 73% yield (75 mg, trans:cis=92:8; data for the trans diastereoisomer); The er was determined by HPLC using a Chiralpack AD-H [hexane/iso-propanol 90:10, λ 220, 1 mL/min] t (major) = 8.59 min., t (minor) = 10.45 min. (94:6). $[\alpha]_D^{20}$ = -61.87 (*c* 0.8, $CHCl_3$); ^{1}H NMR (400 MHz, C_6D_6) δ 1.32 (s, 9H), 1.65 (s, 3H), 3.30 (s, 3H), 4.85 (d, *J* = 2.0 Hz, 1H), 6.64 (d, *J* = 2.0 Hz, 1H), 6.76 (app d, *J* = 8.5 Hz, 2H), 7.37 (app d, *J* = 8.5 Hz, 2H);^{13}C NMR (100 MHz, C_6D_6) δ 24.6 (CH_3), 27.5 (3xCH_3), 54.5 (CH_3), 78.7 (CH), 81.2 (C), 87.2 (C), 113.9 (2xCH), 125.4 (2xCH), 137.9 (C), 154.7 (CH), 159.2 (C), 168.4 (C); IR ν_{max}/cm^{-1} 1744, 1633, 1149, 834. HRMS (ES) calcd $C_{16}H_{121}NNaO_4$ $[M+Na]^+$ 314.1363, found 314.1363.

2.2.3 Synthesis and characterization of (4*S*,5*R*)-*tert*-butyl 5-(5-bromothiophen-2-yl)-5-methyl-4,5-dihydrooxazole-4-carboxylate 4c. The general procedure was followed. The desired product

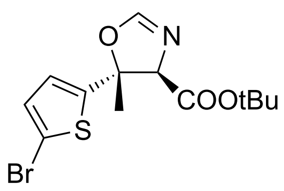

was obtained as a colorless oil in 78% yield (94 mg, trans:cis=88:12; data for the trans diastereoisomer); The er was determined by HPLC using a Chiralpack AD-H [hexane/iso-propanol 90:10, λ 220, 1 mL/min] t (minor) = 6.09 min., t (major) = 8.30 min. (93:7). $[\alpha]_D^{20}$ = -71.7 (*c* 1.00, $CHCl_3$); ^{1}H NMR (400 MHz, C_6D_6) δ 1.27 (s, 9H), 1.54 (s, 3H), 4.73 (d, *J* = 2.0 Hz, 1H), 6.40-6.44 (m, 1H), 6.54 (d, *J* = 4.0 Hz, 1H), 7.16 (s, 1H); ^{13}C NMR (100 MHz, C_6D_6) δ 23.7 (CH_3), 27.5 (3xCH_3), 78.5 (CH), 81.5 (C), 85.1 (C), 111.7 (C), 123.5 (CH), 129.8 (CH), 150.3 (C), 154.2 (CH), 167.3 (C); IR ν_{max}/cm^{-1} 1745, 1632, 1149; HRMS (ES) calcd $C_{13}H_{16}BrNNsO_3S$ $[M+Na]^+$ 367.9926, found 367.9936.

2.2.4 Synthesis and characterization of (4*S*,5*S*)-*tert*-butyl 5-methyl-5-(4-nitrophenyl)-4,5-dihydrooxazole-4-carboxylate 4d. The general procedure was followed. The desired product was

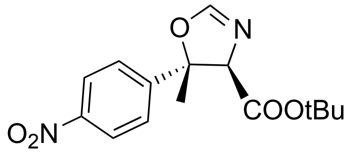

obtained as a colorless oil in 60% yield (64 mg, trans:cis=90:10; data for the trans diastereoisomer); The er was determined by HPLC using a Chiralpack OD-H [hexane/iso-propanol 90:10, λ 220, 1 mL/min] t (minor) = 12.27 min., t (major) = 15.06 min (95:5); $[\alpha]_D^{20}$ = -86.5 (*c* 1.00, $CHCl_3$); ^{1}H NMR (400 MHz, C_6D_6) δ 1.32 (s, 9H), 1.43 (s, 3H), 4.54 (d, *J* = 2.0 Hz, 1H), 6.46 (d, *J* = 2.0 Hz, 1H), 7.11 (d, *J* = 8.0 Hz, 2H), 7.76 (d, *J* = 8.0 Hz, 2H);^{13}C NMR (100 MHz, C_6D_6) δ 24.2 (CH_3), 27.6 (3xCH_3), 78.1 (CH), 81.8 (C), 86.6 (C), 123.6 (2xCH), 124.7 (2xCH), 147.3 (C), 151.7 (C), 154.8 (CH), 168.3 (C); IR ν_{max}/cm^{-1} 1743, 1635, 1522, 1350, 1149, 846; HRMS (ES) calcd $C_{15}H_{18}N_2O_2Na$ $[M+Na]^+$ 329.1108, found 329.1112.

2.2.5 Synthesis and characterization of (4*S*,5*S*)-*tert*-butyl 5-(4-bromophenyl)-5-methyl-4,5-dihydrooxazole-4-carboxylate 4e. The general procedure was followed. The desired product was obtained as a colorless oil in 71% yield (85 mg, trans:cis=89:11; data for the trans diastereoisomer); The er was determined by HPLC using a Chiralpack AD-H [hexane/iso-propanol 90:10, λ 220, 1 mL/min] t (major) = 6.87 min., t (minor) = 8.13 min. (95:5). $[\alpha]_D^{20}$ = -102 (*c* 1.00, $CHCl_3$); ^1H NMR (400 MHz, C_6D_6) δ 1.30 (s, 9H), 1.49 (s, 3H), 4.64 (d, *J* = 2.0 Hz, 1H), 6.54 (d, *J* = 2.0 Hz, 1H), 7.09 (d, *J* = 8.5 Hz, 2H), 7.22 (d, *J* = 8.5 Hz, 2H); ^{13}C NMR (100 MHz, C_6D_6) δ 24.3 (CH_3), 27.5 ($3xCH_3$), 78.3 (CH), 81.5 (C), 86.8 (C), 121.6 (C), 125.9 (2xCH), 131.6 (2xCH), 144.8 (C), 154.4 (CH), 167.9 (C); IR ν_{max}/cm^{-1} 1739, 1630, 1148, 1060; HRMS (ES) calcd $C_{15}H_{19}BrNO_3$ $[M+H]^+$ 340.0543, found 340.0548.

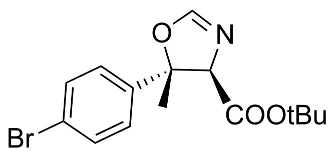

2.2.6 Synthesis and characterization of (4*S*,5*S*)-*tert*-butyl 5-(4-cyanophenyl)-5-methyl-4,5-dihydrooxazole-4-carboxylate 4f. The general procedure was followed. The desired product was obtained as a colorless oil in 80% yield (80 mg, trans:cis=90:10; data for the trans diastereoisomer); The ee was determined by HPLC using a Chiralpack OD-H [hexane/iso-propanol 90:10, λ 220, 1 mL/min] t (minor) = 11.90 min., t (major) = 14.80 min (96:4). $[\alpha]_D^{20}$ = -33.7 (*c* 1.00, $CHCl_3$); ^1H NMR (400 MHz, C_6D_6) δ 1.30 (s, 9H), 1.42 (s, 3H), 4.52 (d, *J* = 2.0 Hz, 1H), 6.50 (d, *J* = 2.0 Hz, 1H), 7.00 (d, *J* = 8.5 Hz, 2H), 7.10 (d, *J* = 8.5 Hz, 2H); ^{13}C NMR (100 MHz, C_6D_6) δ 24.1 (CH_3), 27.5 ($3xCH_3$), 78.1 (CH), 81.7 (C), 86.6 (C), 111.8 (C), 124.6 (2xCH), 132.1 (2xCH), 149.9 (C), 154.2 (CH), 167.7 (C), 118.2 (C); IR ν_{max}/cm^{-1} 1739, 1366, 1216; HRMS (ES) calcd $C_{16}H_{18}N_2O_3Na$ $[M+Na]^+$ 309.1210, found 309.1217.

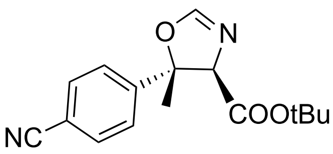

2.2.7 Synthesis and characterization of (4*R*,5*S*)-*tert*-butyl 5-(3-bromo-4-fluorophenyl)-5-methyl-4,5-dihydrooxazole-4-carboxylate 4g. The general procedure was followed. The desired product was obtained as a colorless oil in83% yield (104 mg, trans:cis=85:15; data for the trans diastereoisomer); The er was determined by HPLC using a Chiralpack OD-H [hexane/iso-propanol 90:10, λ 220, 1 mL/min] t (major) = 5.93 min., t (minor) = 6.48 min. (89:11). $[\alpha]_D^{20}$ = -50.78 (*c* 1.28, $CHCl_3$); ^1H NMR (400 MHz, C_6D_6) δ 1.30 (s, 9H), 1.41 (s, 3H), 4.55 (d, *J* = 2.0 Hz, 1H), 6.46 (d, *J* = 2.0 Hz, 1H), 6.60 (app t, *J* = 8.5 Hz, 1H), 7.04 (ddd, *J* = 8.5 Hz, *J* = 6.3 Hz, *J* = 2.3 Hz, 1H), 7.67 (dd, *J* = 6.5 Hz, *J* = 2.3 Hz, 1H); ^{13}C NMR (100 MHz, C_6D_6) δ 24.6 (CH_3), 27.5 ($3xCH_3$), 78.2 (CH), 81.7 (C), 86.2 (C), 109.2 (d, $^2J_{CF}$ = 22.0 Hz, C), 116.4 (d, $^2J_{CF}$ = 21.0 Hz, CH), 124.9 (d, $^3J_{CF}$ = 7.6 Hz, CH), 129.6 (s, CH), 143.2 (d, $^4J_{CF}$ = 4.0 Hz, C), 154.2 (CH), 158.4 (d, $^1J_{CF}$ = 254.0 Hz, C), 167.7 (C); IR ν_{max}/cm^{-1} 1724, 1369, 1216; HRMS (ES) calcd $C_{15}H_{17}BrFNNaO_3$ $[M+Na]^+$ 380.0268, found 380.0274.

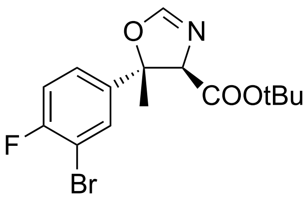

2.2.8 Synthesis and characterization of (4*R*,5*S*)-*tert*-butyl 5-(3,5-bis(trifluoromethyl)phenyl)-5-methyl-4,5-dihydrooxazole-4-carboxylate 4h. The general procedure was followed. The desired

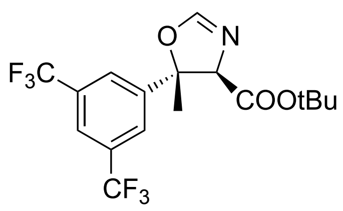

product was obtained as a colorless oil in 82% yield (114 mg, trans:cis=86:14; data for the trans diastereoisomer); The er was determined by HPLC using a Chiralpack AD-H [hexane/iso-propanol 90:10, λ 220, 1 mL/min] t (major) = 5.81 min., t (minor) = 6.41 min. (88:12). $[\alpha]_D^{20}$ = -26.6 (*c* 0.5, $CHCl_3$); ^1H NMR (400 MHz, C_6D_6) δ 1.29 (s, 9H), 1.40 (s, 3H), 4.54 (d, *J* = 2.0 Hz, 1H), 6.41 (d, *J* = 2.0 Hz, 1H), 6.95-6.98 (m, 2H), 7.54 (app d, *J* = 2.0 Hz, 1H);^{13}C NMR (100 MHz, C_6D_6) δ 24.4 (CH_3), 27.5 (3xCH_3), 78.1 (CH), 81.7 (C), 86.2 (C), 123.6 (CH), 126.4 (CH), 130.6 (CH), 131.7 (C), 132.8 (C), 145.9 (C), 154.2 (CH), 167.7 (C); IR ν_{max}/cm^{-1} 1736, 1666, 1370, 1155; HRMS (ES) calcd $C_{17}H_{17}F_6NNaO_3$ $[M+H]^+$ 420.1008, found 420.1005.

2.2.9 Synthesis and characterization of (4*R*,5*R*)-*tert*-butyl 5-methyl-5-(5-methylthiazol-2-yl)-4,5-dihydrooxazole-4-carboxylate 4i. The general procedure was followed. The desired product

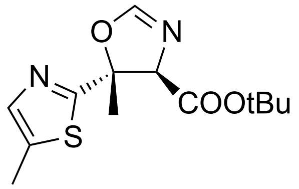

was obtained as a colorless oil in 55% yield (54 mg, trans:cis=91:9; data for the trans diastereoisomer); The er was determined by HPLC using a Chiralpack AD-H [hexane/iso-propanol 90:10, λ 220, 1 mL/min] t (major) = 7.60 min., t (minor) = 9.37 min. (93:7). $[\alpha]_D^{20}$ = -101 (*c* 1.00, $CHCl_3$); ^1H NMR (400 MHz, C_6D_6) δ 1.27 (s, 9H), 1.87 (s, 3H), 2.14 (s, 3H), 5.42 (d, *J* = 2.0 Hz, 1H), 6.20 (d, *J* = 2.0 Hz, 1H), 6.58 (d, *J* = 2.0 Hz, 1H); ^{13}C NMR (100 MHz, C_6D_6) δ 16.7 (CH_3), 22.5 (CH_3), 27.5 (3xCH_3), 77.1 (CH), 81.5 (C), 86.5 (C), 113.8 (CH), 153.2 (C), 154.8 (CH), 167.6 (C), 172.8 (C); IR ν_{max}/cm^{-1} 1738, 1632, 1369, 1152; HRMS (ES) calcd $C_{13}H_{18}N_2NaO_3S$ $[M+Na]^+$ 305.0930, found 305.0931.

2.2.10 Synthesis and characterization of (4*R*,5*R*)-*tert*-butyl 5-methyl-5-(pyrazin-2-yl)-4,5-dihydrooxazole-4-carboxylate 4j. The general procedure was followed. The desired product was

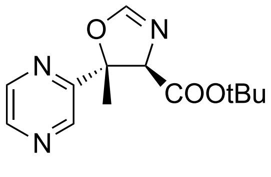

obtained as a colorless oil in 75% yield (69 mg, trans:cis=91:9; data for the trans diastereoisomer); The er was determined by HPLC using a Chiralpack OD-H [hexane/iso-propanol 90:10, λ 220, 1 mL/min] t (minor) = 11.19 min., t (major) = 15.38 min. (91:9). $[\alpha]_D^{20}$ = -62.4 (*c* 1.00, $CHCl_3$); ^1H NMR (200 MHz, C_6D_6) δ 1.30 (s, 9H), 1.70 (s, 3H), 5.20 (d, *J* = 2.0 Hz, 1H), 6.52 (d, *J* = 2.0 Hz, 1H), 7.86 (dd, *J* = 2.5 Hz, *J* = 1.5 Hz, 1H), 7.95 (d, *J* = 2.5 Hz, 1H), 8.69 (d, *J* = 1.5 Hz, 1H); ^{13}C NMR (50 MHz, C_6D_6) δ 22.8 (CH_3), 28.3 (3xCH_3), 77.0 (CH), 82.1 (C), 87.6 (C), 141.9 (CH), 144.1 (CH), 144.7 (CH), 155.8 (CH), 158.7 (C), 168.6 (C); IR ν_{max}/cm^{-1} 1738, 1632, 1154; HRMS (ES) calcd $C_{13}H_{17}N_3NaO_3$ $[M+Na]^+$ 286.1162, found 286.1175.

2.2.11 Synthesis and characterization of (4*S*,5*S*)-*tert*-butyl 5-ethyl-5-phenyl-4,5-dihydrooxazole-4-carboxylate 4k. The general procedure was followed. The desired product was obtained as a colorless oil in 73 yield (71 mg, trans:cis=90:10; data for the trans diastereoisomer); The er was determined by HPLC using a Chiralpack AD-H [hexane/iso-propanol 90:10, λ 220, 1 mL/min] t (major) = 5.35 min., t (minor) = 7.93 min (99:1). $[\alpha]_D^{20}$ = -51.6 (*c* 0.5, $CHCl_3$); ^{1}H NMR (400 MHz, C_6D_6) δ 0.75 (t, *J* = 7.5 Hz, 3H), 1.33 (s, 9H), 1.95-2.05 (m, 1H), 2.08-2.18 (m, 1H), 4.81 (d, *J* = 2.0 Hz, 1H), 6.63 (d, *J* = 2.0 Hz, 1H), 7.03-7.08 (m, 1H), 7.13-7.18 (m, 2H), 7.42-7.47 (m, 2H); ^{13}C NMR (100 MHz, C_6D_6) δ 8.3 (CH_3), 27.6 (3xCH_3), 30.0 (CH_2), 79.2 (CH), 81.2 (C), 90.2 (C), 124.1 (2xCH), 127.5 (CH), 128.6 (2xCH), 146.1 (C), 154.8 (C), 168.3 (C); IR ν_{max}/cm^{-1} 1787, 1687, 1152, 701; HRMS (ES) calcd $C_{15}H_{22}NO_3$ $[M+H]^+$ 276.1594, found 276.1597.

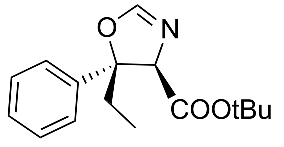

2.2.12 Synthesis and characterization of (4*R*,5*S*)-*tert*-butyl 5-ethyl-5-(*p*-tolyl)-4,5-dihydrooxazole-4-carboxylate 4l. The general procedure was followed. The desired product was obtained as a colorless oil in 81% yield (82 mg, trans:cis=91:9; data for the trans diastereoisomer); The er was determined by HPLC using a Chiralpack AD-H [hexane/iso-propanol 90:10, λ 220, 1 mL/min] t (major) = 6.69 min., t (minor) = 14.12 min (98:2). $[\alpha]_D^{20}$ = +80.5 (*c* 1.0, $CHCl_3$); ^{1}H NMR (400 MHz, C_6D_6) δ 0.81 (t, *J* = 8.0 Hz, 3H), 1.35 (s, 9H), 1.99-2.07 (m, 1H), 2.09 (s, 3H), 2.13-2.21 (m, 1H), 4.86 (d, *J* = 2.0 Hz, 1H), 6.63 (d, *J* = 2.0 Hz, 1H), 6.99 (d, *J* = 8.0 Hz, 1H), 7.38 (d, *J* = 8.0 Hz, 1H);^{13}C NMR (100 MHz, C_6D_6) δ 8.3 (CH_3), 20.6 (CH_3), 27.6 (3xCH_3), 30.0 (CH_2), 79.4 (CH), 81.1 (C), 90.3 (C), 124.6 (2xCH), 129.1 (2xCH), 136.7 (C), 141.2 (C), 154.5 (CH), 168.3 (C); IR ν_{max}/cm^{-1} 2977, 1744, 1636, 1153; HRMS (ES) calcd $C_{17}H_{24}NO_3$ $[M+H]^+$ 290.1751, found 290.1756.

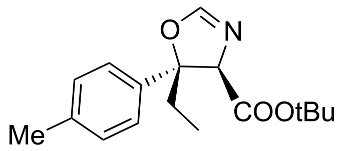

2.2.13 Synthesis and characterization of (4*R*,5*S*)-*tert*-butyl 5-ethyl-5-(4-fluorophenyl)-4,5-dihydrooxazole-4-carboxylate 4m. The general procedure was followed. The desired product was obtained as a colorless oil in 83% yield (85 mg, trans:cis=88:12; data for the trans diastereoisomer); The er was determined by HPLC using a Chiralpack AD-H [hexane/iso-propanol 90:10, λ 220, 1 mL/min] t (major) = 4.98 min., t (minor) = 7.02 min (99:1). $[\alpha]_D^{20}$ = -44.5 (*c* 1.34, $CHCl_3$); ^{1}H NMR (400 MHz, C_6D_6) δ 0.69 (t, *J* = 8.0 Hz, 3H), 1.34 (s, 9H), 1.84-1.93 (m, 1H), 2.02-2.11 (m, 1H), 4.70 (d, *J* = 2.0 Hz, 1H), 6.59 (d, *J* = 2.0 Hz, 1H), 6.65-6.75 (m, 2H), 7.21-7.25 (m, 2H);^{13}C NMR (100 MHz, C_6D_6) δ 8.3 (CH_3), 27.5 (3xCH_3), 30.0 (CH_2), 79.2 (CH), 81.4 (C), 89.8 (C), 115.2 (d, J_{CF} = 21.0 Hz, CH), 126.5 (d, J_{CF} = 8.0 Hz, CH), 139.6 (d, J_{CF} = 3.0 Hz, C), 154.4 (CH), 162.1 (d, J_{CF} = 243.0 Hz, C), 168.1 (C); IR ν_{max}/cm^{-1} 2978, 1674, 1510, 1153; HRMS (ES) calcd $C_{16}H_{20}FNNaO_3$ $[M+Na]^+$ 316.1319, found 316.1320.

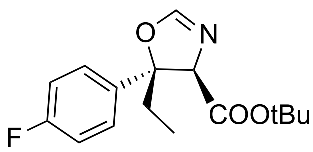

2.2.14 Synthesis and characterization of (4*R*,5*S*)-*tert*-butyl 5-(4-bromophenyl)-5-ethyl-4,5-dihydrooxazole-4-carboxylate 4n. The general procedure was followed. The desired product was

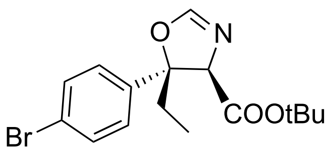

obtained as a colorless oil in 73% yield (90 mg, trans:cis=90:10; data for the trans diastereoisomer); The er was determined by HPLC using a Chiralpack AD-H [hexane/iso-propanol 90:10, λ 220, 1 mL/min] t (major) = 5.81 min., t (minor) = 8.97 min (99:1). $[\alpha]_D^{20}$ = -110.8 (*c* 1.0, $CHCl_3$); ^{1}H NMR (400 MHz, C_6D_6) δ 0.67 (t, *J* = 8.0 Hz, 3H), 1.33 (s, 9H), 1.81-1.90 (m, 1H), 2.00-2.09 (m, 1H), 4.65 (d, *J* = 2.0 Hz, 1H), 6.54 (d, *J* = 2.0 Hz, 1H), 7.09 (d, *J* = 8 Hz, 2H), 7.24 (d, *J* = 8 Hz, 2H);^{13}C NMR (100 MHz, C_6D_6) δ 8.1 (CH_3), 27.5 (3xCH_3), 29.7 (CH_2), 79.0 (CH), 81.4 (C), 89.7 (C), 121.4 (C), 126.5 (2xCH), 131.6 (2xCH), 142.9 (C), 154.3 (CH), 167.9 (C); IR ν_{max}/cm^{-1} 2970, 1739, 1368, 1216; HRMS (ES) calcd $C_{16}H_{21}NBrO_3$ $[M+H]^+$ 354.0699, found 354.0691.

2.2.15 Synthesis and characterization of (4*R*,5*S*)-*tert*-butyl 5-phenyl-5-propyl-4,5-dihydrooxazole-4-carboxylate 4o. The general procedure was followed. The desired product was

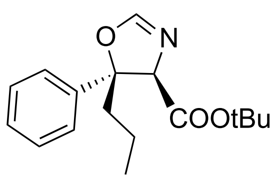

obtained as a colorless oil in 79% yield (80 mg, trans:cis=87:13; data for the trans diastereoisomer); The er was determined by HPLC using a Chiralpack AD-H [hexane/iso-propanol 90:10, λ 220, 1 mL/min] t (major) = 4.93 min., t (minor) = 7.20 min (99:1). $[\alpha]_D^{20}$ = +89.2 (*c* 1.0, $CHCl_3$); ^{1}H NMR (400 MHz, C_6D_6) δ 0.70 (t, *J* = 8.0 Hz, 3H), 1.00-1.12 (m, 1H), 1.37 (s, 9H), 1.40-1.46 (m, 1H), 1.98-2.17 (m, 2H), 4.85 (d, *J* = 2.0 Hz, 1H), 6.61 (d, *J* = 2.0 Hz, 1H), 7.04-7.07 (m, 1H), 7-14-7.18 (m, 2H), 7.47-7.49 (m, 2H); ^{13}C NMR (100 MHz, C_6D_6) δ 13.8 (CH_3), 17.5 (CH_2), 27.6 (3xCH_3), 39.5 (CH_2), 79.4 (CH), 81.3 (C), 89.8 (C), 124.5 (2xCH), 127.2 (CH), 128.4 (2xCH), 144.6 (C), 154.5 (CH), 168.3 (C); IR ν_{max}/cm^{-1} 2962, 1635, 1153

2.2.16 Synthesis and characterization of (4*R*,5*R*)-*tert*-butyl 5-propyl-5-(thiophen-2-yl)-4,5-dihydrooxazole-4-carboxylate 4p. The general procedure was followed. The desired product was

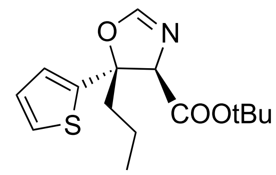

obtained as a colorless oil in 81% yield (84 mg, trans:cis=84:16; data for the trans diastereoisomer); The er was determined by HPLC using a Chiralpack AD-H [hexane/iso-propanol 90:10, λ 220, 1 mL/min] t (major) = 5.93 min., t (minor) = 8.22 min (96:4). $[\alpha]_D^{20}$ = -29.8 (*c* 0.5, $CHCl_3$); ^{1}H NMR (400 MHz, C_6D_6) δ 0.73 (t, *J* = 8.0 Hz, 3H), 1.12-1.32 (m, 1H), 1.35 (s, 9H), 1.44-1.53 (m, 1H), 2.02-2.16 (m, 2H), 4.89 (d, *J* = 2.0 Hz, 1H), 6.54 (d, *J* = 2.0 Hz, 1H), 6.69 (dd, *J* = 5.0 Hz, *J* = 3.5 Hz, 1H), 6.79 (dd, *J* = 5.0 Hz, *J* = 1.0 Hz, 1H), 6.92 (dd, *J* = 3.5 Hz, *J* = 1.0 Hz, 1H); ^{13}C NMR (100 MHz, C_6D_6) δ 13.8 (CH_3), 17.6 (CH_2), 27.5 (3xCH_3), 39.6 (CH_2), 79.8 (CH), 81.3 (C), 88.7 (C), 122.9 (CH), 124.2 (CH), 126.9 (CH), 148.1 (C), 154.4 (CH), 167.7 (C); IR ν_{max}/cm^{-1} 2969, 1742, 1634, 1368, 1153; HRMS (ES) calcd $C_{15}H_{21}NNaO_3S$ $[M+Na]^+$ 318.1134, found 318.1135.

2.2.17 Synthesis and characterization of (4*R*,5*S*)-*tert*-butyl 5-isobutyl-5-phenyl-4,5-dihydrooxazole-4-carboxylate 4q. The general procedure was followed. The desired product was

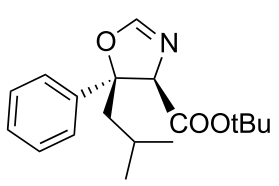

obtained as a colorless oil in 75% yield (80 mg, trans:cis=96:4; data for the trans diastereoisomer); The er was determined by HPLC using a Chiralpack AD-H [hexane/iso-propanol 90:10, λ 220, 1 mL/min] t (minor) = 5.77 min., t (major) = 7.10 min (97:3). $[\alpha]_D^{20}$ = -69 (*c* 1.0, $CHCl_3$); ^{1}H NMR (400 MHz, C_6D_6) δ 0.62 (d, *J* = 8.0 Hz, 3H), 0.92 (d, *J* = 8.0 Hz, 3H), 1.38 (s, 9H), 1.48-1.60 (m, 1H), 1.99 (dd, *J* = 14.0 Hz, *J* = 8.0 Hz, 1H), 2.15 (dd, *J* = 14.0 Hz, *J* = 4.0 Hz, 1H), 4.80 (d, *J* = 2.0 Hz, 1H), 6.64 (d, *J* = 1.0 Hz, 1H), 7.02-7.06 (m, 1H), 7-13-7.16 (m, 1H), 7.43-7.47 (m, 2H); ^{13}C NMR (100 MHz, C_6D_6) δ 22.9 (CH_3), 24.1 (CH_3), 24.6 (CH_3), 27.6 (3xCH_3), 45.3 (CH_2), 80.7 (CH), 81.2 (C), 90.0 (C), 124.4 (2xCH), 127.2 (CH), 128.5 (2xCH), 144.6 (C), 154.7 (CH), 168.2 (C); IR ν_{max}/cm^{-1} 2956, 1637, 1150; HRMS (ES) calcd $C_{18}H_{25}NNaO_3$ $[M+Na]^+$ 326.1727, found 326.1729.

2.1.18 Synthesis and characterization of (4*R*,5*S*)-ethyl 5-ethyl-5-phenyl-4,5-dihydrooxazole-4-carboxylate 4r. The general procedure was followed. The desired product was obtained as a

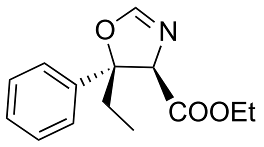

colorless oil in 81% yield (70 mg, trans:cis=90:10; data for the trans diastereoisomer); The er was determined by HPLC using a Chiralpack AD-H [hexane/iso-propanol 90:10, λ 220, 1 mL/min] t (major) = 7.51 min., t (minor) = 10.19 min (99:1). $[\alpha]_D^{20}$ = -89.0 (*c* 0.76, $CHCl_3$); ^{1}H NMR (400 MHz, C_6D_6) δ 0.72 (t, *J* = 4.0 Hz, 3H), 0.92 (t, *J* = 4.0 Hz, 3H), 1.87-1.96 (m, 1H), 2.00-2.08 (m, 1H), 3.93-3.99 (m, 2H), 4.89 (d, *J* = 1.0 Hz, 1H), 6.63 (d, *J* = 1.0 Hz, 1H), 7.03-7.06 (m, 1H), 7.12-7.16 (m, 2H), 7.41-7.43 (m, 2H);^{13}C NMR (100 MHz, C_6D_6) δ 8.2 (CH_3), 13.8 (CH_3), 30.1 (CH_2), 60.7 (CH_2), 78.7 (CH), 90.2 (C), 124.6 (2xCH), 127.3 (CH), 128.5 (2xCH_2), 143.8 (C), 154.8 (CH), 169.1 (C); IR ν_{max}/cm^{-1} 2956, 1637, 1150; HRMS (ES) calcd $C_{14}H_{18}NO_3$ $[M+H]^+$ 248.1281, found 248.1289.

2.2.19 Synthesis and characterization of (4*R*,5*S*)-methyl 5-ethyl-5-(*p*-tolyl)-4,5-dihydrooxazole-4-carboxylate 4s. The general procedure was followed. The desired product was

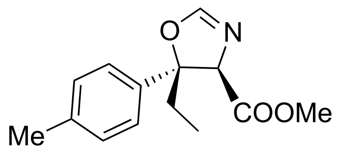

obtained as a colorless oil in 82% yield (71 mg, trans:cis=91:9; data for the trans diastereoisomer); The er was determined by HPLC using a Chiralpack AD-H [hexane/iso-propanol 90:10, λ 220, 1 mL/min] t (major) = 7.74 min., t (minor) = 9.16 min (98:2). $[\alpha]_D^{20}$ = -49.0 (*c* 0.1, $CHCl_3$); ^{1}H NMR (400 MHz, C_6D_6) δ 0.73 (t, *J* = 4.0 Hz, 3H), 1.81-1.88 (m, 1H), 1.90-2.01 M, 1H), 2.08 (s, 3H), 3.33 (s, 3H), 4.90 (d, *J* = 2.0 Hz, 1H), 6.63 (d, *J* = 1.0 Hz, 1H), 6.97 (d, *J* = 8 Hz, 2H), 7.30 (d, *J* = 8 Hz, 2H);^{13}C NMR (100 MHz, C_6D_6) δ 8.2 (CH_3), 20.6 (CH_3), 30.0 (CH_2), 51.2 (CH_3), 78.8 (CH), 90.2 (C), 124.5 (2xCH), 129.2 (2xCH), 136.8 (C), 140.8 (C), 154.9 (CH), 169.6 (C); IR ν_{max}/cm^{-1} 1744, 1635, 1153; HRMS (ES) calcd $C_{14}H_{17}NNaO_3$ $[M+Na]^+$ 270.1101, found 270.1102.

2.2.20 Synthesis and characterization of (4*R*,5*S*)-methyl 5-ethyl-5-phenyl-4,5-dihydrooxazole-4-carboxylate 4t. The general procedure was followed. The desired product was obtained as a

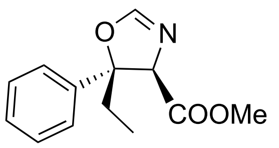

colorless oil in 77% yield (71 mg, trans:cis=91:9; data for the trans diastereoisomer); The er was determinated by HPLC using a Chiralpack AD-H [hexane/iso-propanol 90:10, λ 220, 1 mL/min] t (major) = 8.27 min., t (minor) = 10.84 min (99:1). $[\alpha]_D^{20}$ = -54.3 (*c* 1.0, $CHCl_3$); ^{1}H NMR (400 MHz, C_6D_6) δ 0.68 (t, *J* = 4.0 Hz, 3H), 1.78-1.87 (m, 1H), 1.91-2.00 (m, 1H), 3.31 (s, 3H), 4.87 (d, *J* = 2.0 Hz, 1H), 6.60 (d, *J* = 1.0 Hz, 1H), 7.02-7.06 (m, 1H), 7.11-7.16 (m, 2H), 7.37-7.39 (m, 2H); ^{13}C NMR (100 MHz, C_6D_6) δ 8.1 (CH_3), 30.1 (CH_2), 51.2 (CH_3), 78.7 (CH), 90.1 (C), 124.6 (2xCH), 127.3 (CH), 128.5 (2xCH), 143.7 (C), 154.8 (CH), 169.5 (C); IR ν_{max}/cm^{-1} 1738, 1368, 1228, 1216; HRMS (ES) calcd $C_{13}H_{16}NO_3$ $[M+H]^+$ 234.1125, found 234.1129.

2.1.21 Synthesis and characterization of (4*R*,5*R*)-methyl 5-(azidomethyl)-5-phenyl-4,5-dihydrooxazole-4-carboxylate 4u. The general procedure was followed. The desired product was

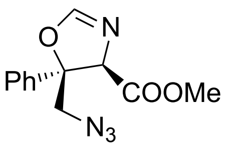

obtained as a colorless oil in 83% yield (75 mg, trans:cis=77:23; data for the trans diastereoisomer); The er was determined by HPLC using a Chiralpack AD-H [hexane/iso-propanol 90:10, λ 220, 1 mL/min] t (major) = 14.03 min., t (minor) = 26.64 min (98:2). $[\alpha]_D^{20}$ = -30.3 (*c* 0.61, $CHCl_3$); ^{1}H NMR (400 MHz, C_6D_6) δ 3.15 (d, *J* = 16.0 Hz, 1H), 3.39 (s, 3H), 3.44 (d, *J* = 16.0 Hz, 1H), 4.73 (d, *J* = 2.0 Hz, 1H), 6.48 (d, *J* = 1.0 Hz, 1H), 7.03-7.16 (m, 3H), 7.39 (d, *J* = 8.0 Hz, 2H); ^{13}C NMR (100 MHz, C_6D_6) δ 51.8 (CH), 56.0 (CH_2), 76.0 (CH), 89.2 (C), 124.7 (2xCH), 127.9 (CH), 128.7 (2xCH), 141.5 (C), 154.1 (CH), 169.4 (C); IR ν_{max}/cm^{-1} 1738, 1436, 1216; HRMS (ES) calcd $C_{12}H_{12}N_4O_3Na$ $[M+Na]^+$ 283.0801, found 283.0795.

2.1.22 Synthesis and characterization of (4*R*,5*S*)-methyl 5-(2-azidoethyl)-5-phenyl-4,5-dihydrooxazole-4-carboxylate 4v. The general procedure was followed. The desired product was

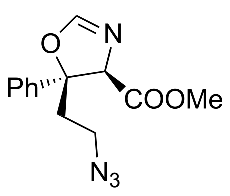

obtained as a colorless oil in 79% yield (63 mg, trans:cis=76:24; data for the trans diastereoisomer); The er was determined by HPLC using a Chiralpack AD-H [hexane/iso-propanol 90:10, λ 220, 1 mL/min] t (major) = 13.10 min., t (minor) = 17.17 min (97:3). $[\alpha]_D^{20}$ = -43.60 (*c* 0.83, $CHCl_3$); ^{1}H NMR (400 MHz, C_6D_6) δ 2.02-2.14 (m, 2H), 2.61-2.68 (m, 1H), 2.87-2.94 (m, 1H), 3.26 (s, 3H), 4.75 (d, *J* = 1.0 Hz, 1H), 6.48 (d, *J* = 1.0 Hz, 1H), 7.02-7.10 (m, 3H), 7.22-7.24 (m, 2H); ^{13}C NMR (100 MHz, C_6D_6) δ 35.9 (CH_2), 46.7 (CH_2), 51.4 (CH_3), 79.1 (CH), 87.7 (C), 124.1 (2xCH), 127.8 (CH), 128.8 (2xCH), 142.6 (C), 154.6 (CH), 169.1 (C); IR ν_{max}/cm^{-1} 2970, 1739, 1368, 1215; HRMS (ES) calcd $C_{13}H_{15}N_4O_3$ $[M+H]^+$ 275.1138, found 235.1135.

2.3 General procedure for the preparation of racemic oxazolines

Ag_2O (6.1 mg, 0.026 mmol), PPh_3 (9.2 mg, 0.03 mmol) were dissolved in 2.0 mL of EtOAc in presence of powdered 4A MS. After that, Et_3N (24 μL, 0.17mmol) and the ketone **3** (0.386 mmol, 1.1 equiv) was added. The heterogeneous mixture was cooled at -20 ºC in a fridge and stirred for 30 min. After that, the isocyanate **2c** (50 mg, 0.351 mmol, 1.0 equiv) previously dissolved in 2.0 mL of EtOAc and cooled at -20 ºC, was added. The reaction mixture was stirred at the same temperature for 72 h (time needed to the total consumption of the isocyanacetate, according with the TLC). The reaction mixture was then quickly filtered through a short pad of celite (in a glass pipette) and eluted with EtOAc. The filtrate was concentrated under reduce pressure and purified by flash column chromatography purified on silica gel using mixtures of petroleum ether/ethyl acetate (9/1→3/1→1/1) to yield racemic oxazolines **4** as pure compounds.

The racemic samples of **4l**, **4q**, **4r**, **4p**, **4t**, **4u**, **4v**, **5a** and **5b** were prepared by mixing equimolar of both enantiomers. The other enantiomer was synthesised in analogous fashion using the ligand **1b** (the pseudoenantiomer of **1a**) following the general procedure for the preparation the oxazolines **4**.

2.4 Synthesis and characterization of compounds 5

2.4.1 Syntesis and characterization of (3a*S*,6a*S*)-6a-phenyl-6,6a-dihydro-3a*H*-pyrrolo[3,4-*d*]oxazol-4(5*H*)-one 5a

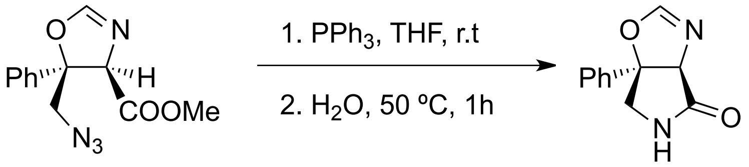

4u **5a**

Oxazoline **4u** (146 mg, 0.56 mmol) was dissolved in 5 mL of dry THF and PPh_3 (147 mg, 0.56 mmol, 1.0 equiv) was added. The reaction mixture was stirring at room temperature for the time necessary to form the iminophosphorane product (following by mass spectrum). Then H_2O (20 μL, 1.12 mmol, 2.0 equiv) was added and the reaction mixture was heating at 50 ºC for 1 h. After that, the reaction mixture was concentrated under reduce pressure and purified by chromatography column on silica gel using mixtures of petroleum ether/ethyl acetate (3/1→1/1→1/2) to yield the bycyclic product **5a** (94 mg, 83%) as a colorless oil compound. The er was determined by HPLC using a Chiralpack AD-H [hexane/iso-propanol 70:30, λ 220, 1 mL/min] t (minor) = 8.85 min., t (minor) = 23.06 min (98:2). $[\alpha]_D^{20}$: +47 (c 0.1, $CHCl_3$); ^1H NMR (400 MHz, $CDCl_3$) δ 3.05 (d, J = 12.0 Hz, 1H), 3.10 (d, J = 12.0 Hz, 1H), 3.76 (s, 1H), 6.19 (s, 1H), 6.44-6.60 (m, 5H), 7.22 (s, 1H); ^{13}C NMR (100 MHz, $CDCl_3$) δ 54.9 (CH_2), 76.4 (CH), 88.8 (C), 123.4 (2xCH), 127.9 (CH), 128.4 (2xCH), 139.2 (C), 154.7 (CH), 172.8 (C); IR ν_{max}/cm^{-1} 3262, 1708, 762; HRMS (ES) calcd $C_{11}H_{11}N_2O_2$ $[M+H]^+$ 203.0815, found 203.0809.

2.4.2 Syntesis and characterization of (3a*S*,7a*R*)-7a-phenyl-5,6,7,7a-tetrahydrooxazolo[4,5-*c*]pyridin-4(3a*H*)-one 5b

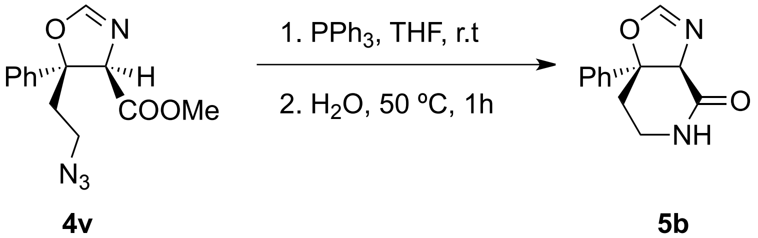

Oxazoline **4v** (95 mg, 0.35 mmol) was dissolved in 8 mL of dry THF and PPh_3 (92 mg, 0.35 mmol, 1.0 equiv) was added. The reaction mixture was stirring at room temperature for the time necessary to form the iminophosphorane product (following by mass spectrum). Then H_2O (12.6 μL, 0.7 mmol, 2.0 equiv) was added and the reaction mixture was heating at 50 ºC for 1 h. After that, the reaction mixture was concentrated under reduced pressure and purified by chromatography column on silica gel using mixtures of petroleum ether/ethyl acetate (3/1→1/1→1/2) to yield the bycyclic product **5b** (55 mg, 73%) as a colorless oil. The er was determined by HPLC using a Chiralpack AD-H [hexane/iso-propanol 80:20, λ 220, 1 mL/min] t (minor) = 11.64 min., t (major) = 23.76 min (97:3). $[\alpha]_D^{20}$: -5.00 (c 0.1, $CHCl_3$); ^{1}H-NMR ($CDCl_3$, 400 MHz) δ 2.13-2.28 (m, 2H), 3.18-3.28 (m, 1H), 3.32-3.38 (m, 1H), 4.58 (s, 1H), 7.08 (s, 1H), 7.27-7.35 (m, 5H), 7.78 (s, 1H); ^{13}C-NMR ($CDCl_3$) δ 35.9 (CH_2), 37.1 (CH_2), 74.7 (CH), 87.5 (C), 124.3 (2xCH), 128.3 (CH), 128.9 (2xCH), 142.9 (C), 156.2 (CH), 169.0 (C); IR ν_{max}/cm^{-1} 3258, 1670, 759; HRMS (ES) calcd $C_{12}H_{13}N_2O_2$ $[M+H]^+$ 217.0967, found 217.0971.

2.5 Synthesis and characterization of compound 6

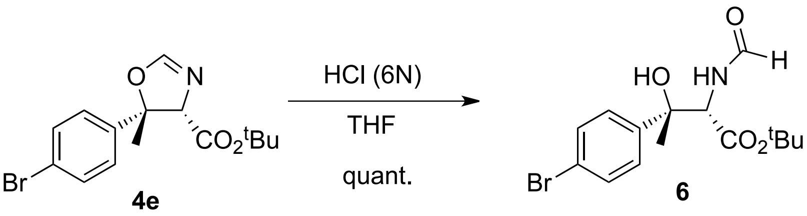

Oxazoline **4e** (50 mg, 0.2 mmol) was dissolved in 3 mL of THF and 5 pipette drops of HCl (6N) were added. The reaction mixture was stirred at room temperature for 4 h. Removal of volatiles under reduced pressure yielded the corresponding opened product **6** in quantitative yield. The er was determined by HPLC using a Chiralpack OD-H [hexane/iso-propanol (80:20, λ 220, 1 mL/min] t (major) = 5.31 min., t (minor) = 8.01 min (95:5). $[\alpha]_D^{20}$ = -48.0 (*c* 0.1, $CHCl_3$); ^{1}H NMR (200 MHz, $CDCl_3$) δ 1.47 (s, 9H), 1.59 (s, 3H), 3.68 (brs, 1H), 4.82 (d, J = 8 Hz, 1H), 6.41 (d, J = 8 Hz, 1H), 7.32 (d, J = 10 Hz, 2H), 7.46 (d, J = 8 Hz, 2H), 8.00 (s, 1H); ^{13}C NMR (50 MHz, $CDCl_3$) δ 28.1 (CH_3), 59.4 (CH_3), 75.5 (C), 84.2 (C), 121.96 (C), 127.4 (2xCH), 131.8 (2xCH), 143.00 (C), 161.2 (CH), 170.6 (C); IR ν_{max}/cm^{-1} 1728, 1243; HRMS (ES) calcd $C_{15}H_{20}NNaBrO_4$ $[M+Na]^+$ 380.0467, found 380.0461.

2.6 Synthesis and characterization of compound 7

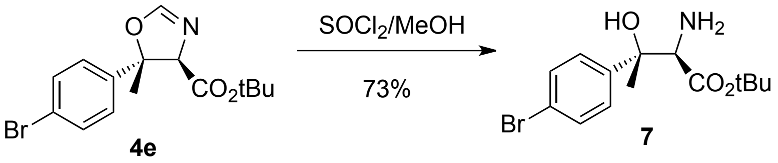

Oxazoline **4e** (281 mg, 0.82 mmol) was dissolved in 8 mL of dry MeOH. The reaction mixture was cooled to 0 ºC and 8 mL of 1M solution of $SOCl_2$ in MeOH (2M HCl) were added dropwise. The solution was stirred 1h 45′ at 0 ºC and then the solvent was removed under reduced pressure. The resultant oil was dissolved in CH_2Cl_2 (10 mL) and few drops of sat. $NaHCO_3$ solution were added under vigorous stirring until P_H 8 (PH paper). The mixture was further diluted with CH_2Cl_2 and dried with Na_2SO_4. The precipites were filtered off and the resultant solution was concentrated under reduced pressure and the crude was purified by FCC (AcOEt/PE 2:1) to yield the amino-ester **7** (138 mg, 73%) as an oil compound. ^{1}H NMR (400 MHz, $CDCl_3$) δ 1.31 (s, 9H), 1.44 (s, 3H), 3.45 (brs, 1H), 4.03 (brs, 1H), 5.22 (s, 1H), 7.28 (d, J = 10 Hz, 2H), 7.39 (d, J = 8 Hz, 2H); ^{13}C NMR (100 MHz, $CDCl_3$) δ 25.2 (3xCH_3), 27.9 (CH_3), 53.5 (C), 63.4 (C), 82.3 (CH), 121.1 (C), 127.6 (2xCH), 131.1 (2xCH), 144.2 (C), 172.8 (C); IR ν_{max}/cm^{-1} 2978, 1725, 1368, 1156; HRMS (ES) calcd $C_{14}H_{20}NNaBrO_3$ $[M+Na]^+$ 352.0518, found 352.0513.

2.6 Synthesis and characterization of compound 8

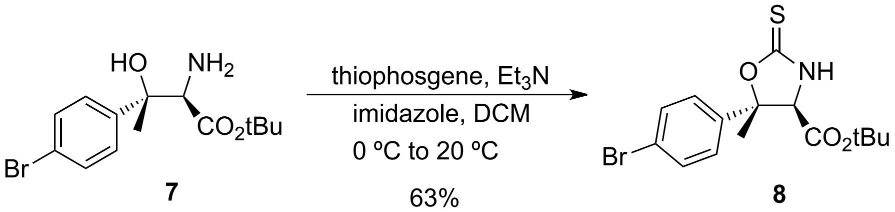

Amino-ester **7** (485 mg, 1.47 mmol) was dissolved in 40 mL of CH_2Cl_2 and the solution was cooled to 0 ºC. Then Et_3N (0.20 mL, 1.44 mmol), imidazole (98.35 mg, 1.44 mmol) and thiophosgene (0.122 mL, 1.54 mmol) were added. The reaction mixture was stirred 12 h at 20 ºC and then was washed with water (10 mL) and brine (10 mL) and finally dried with Na_2SO_4. The reaction mixture was filtered and the resultant solution

concentrated under reduced pressure and purified by chromatography column on silica gel using mixtures of petroleum ether/ethyl acetate (3/1→1/1→1/2) to yield the ciclic product **8** (344 mg, 63%) as a solid compound. The product was recrystallized using EtOAc. The er after recrystallization was determined by HPLC using a Chiralpack AD-H [hexane/iso-propanol 90:10, λ 220, 1 mL/min] t (minor) = 9.73 min., t (major) = 21.58 min (99:1). $[\alpha]_D^{20}$: 82 (c 0.25, $CHCl_3$); Mp = 175-177 ºC; ^{1}H NMR (400 MHz, $CDCl_3$) δ 1.48 (s, 9H), 1.68 (s, 3H), 4.45 (s, 1H), 7.32 (d, J = 8.0 Hz, 2H), 7.46 (d, J = 8.0 Hz, 2H), 8.03 (s, 1H); ^{13}C NMR (100 MHz, $CDCl_3$) δ 24.3 (CH_3), 28.0 (3x CH_3), 68.2 (CH), 84.9 (C), 91.4 (C), 122.7 (C), 126.1 (2xCH), 132.0 (2xCH), 141.5 (C), 166.1

(C), 188.1 (C); IR ν_{max}/cm^{-1} 2979, 1743, 1491, 1230, 1152; HRMS (ES) calcd $C_{15}H_{19}NO_3BrS$ $[M+H]^+$ 372.0263, found 372.0260.

3 ^{1}H-NMR and ^{13}C-NMR spectra of pre-catalysts

3.1 ^{1}H and ^{13}C-NMR spectra of compound 1a

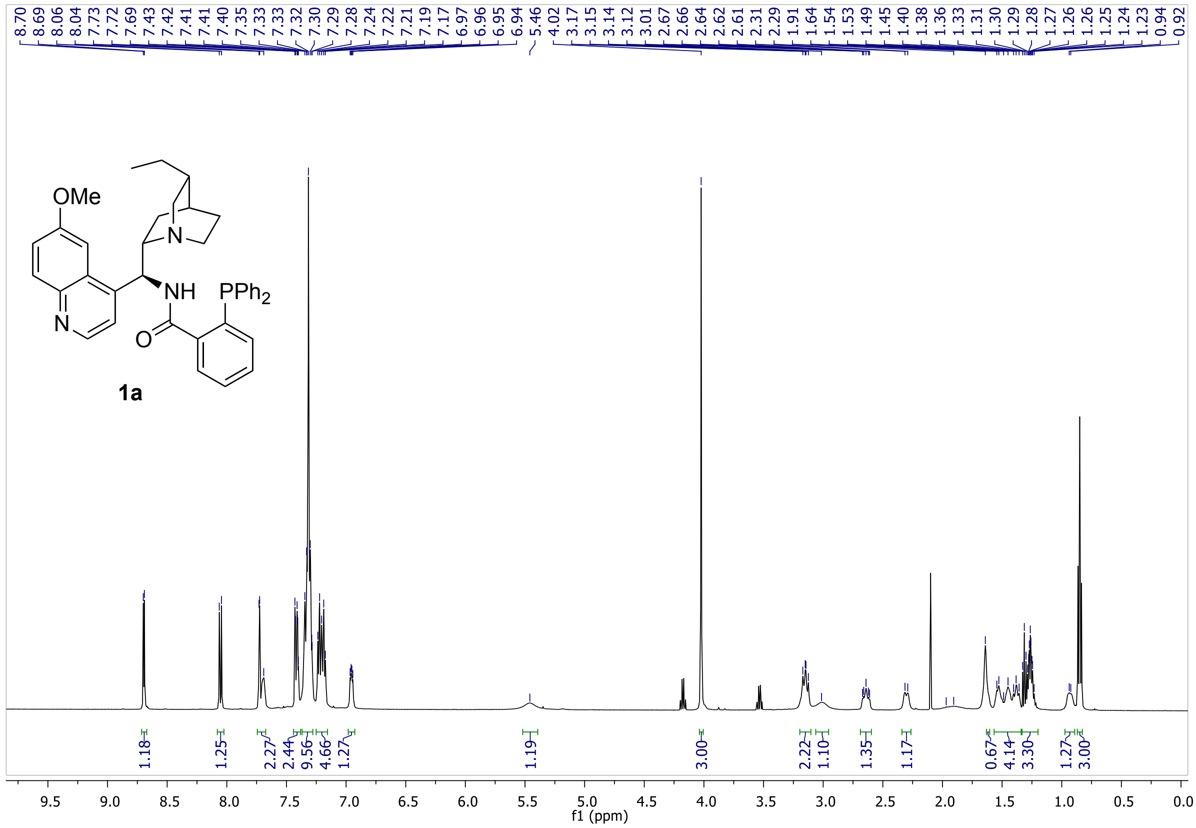

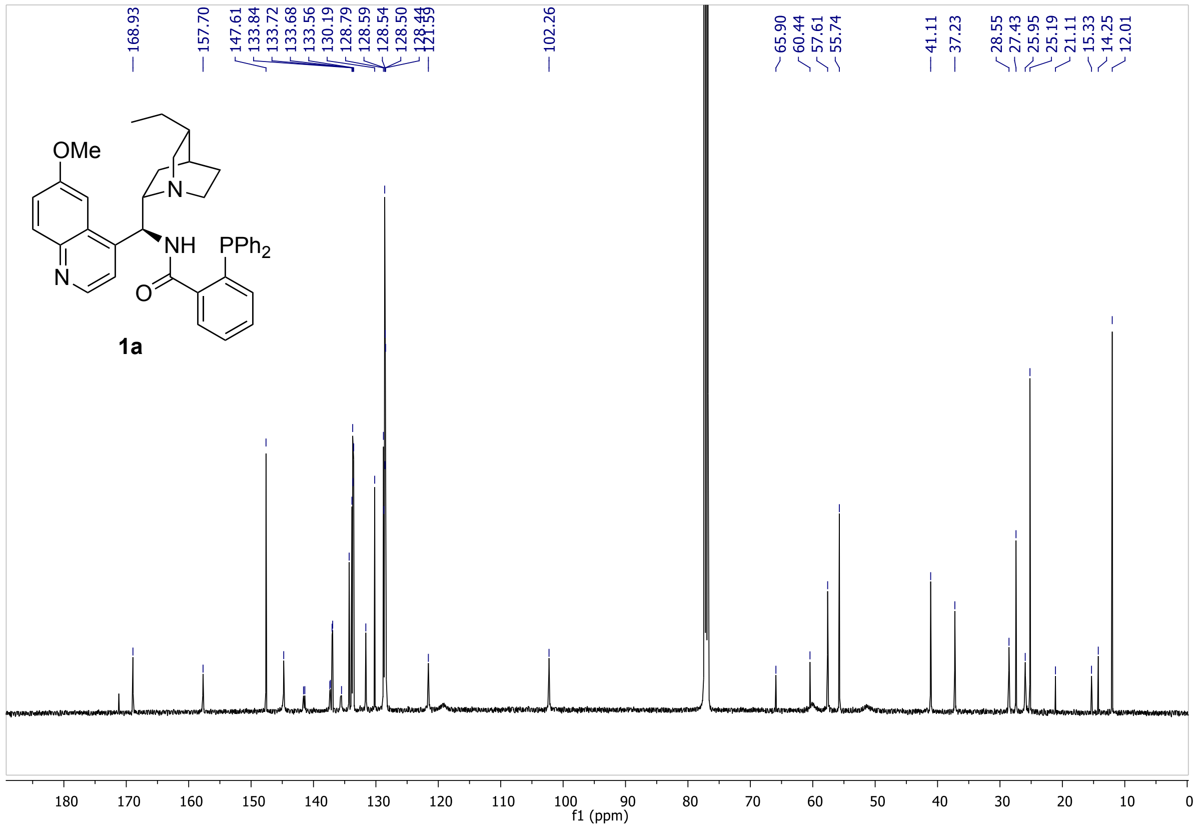
168.93
157.70
147.61
133.84
133.72
133.68
133.56
130.19
128.79
128.59
128.54
128.50
128.44
121.59
102.26
65.90
60.44
57.61
55.74
41.11
37.23
28.55
27.43
25.95
25.19
21.11
15.33
14.25
12.01
OMe
N
NH
PPh2
N
O
1a
f1 (ppm)

3.2 ^{1}H and ^{13}C-NMR spectra of compound 1b

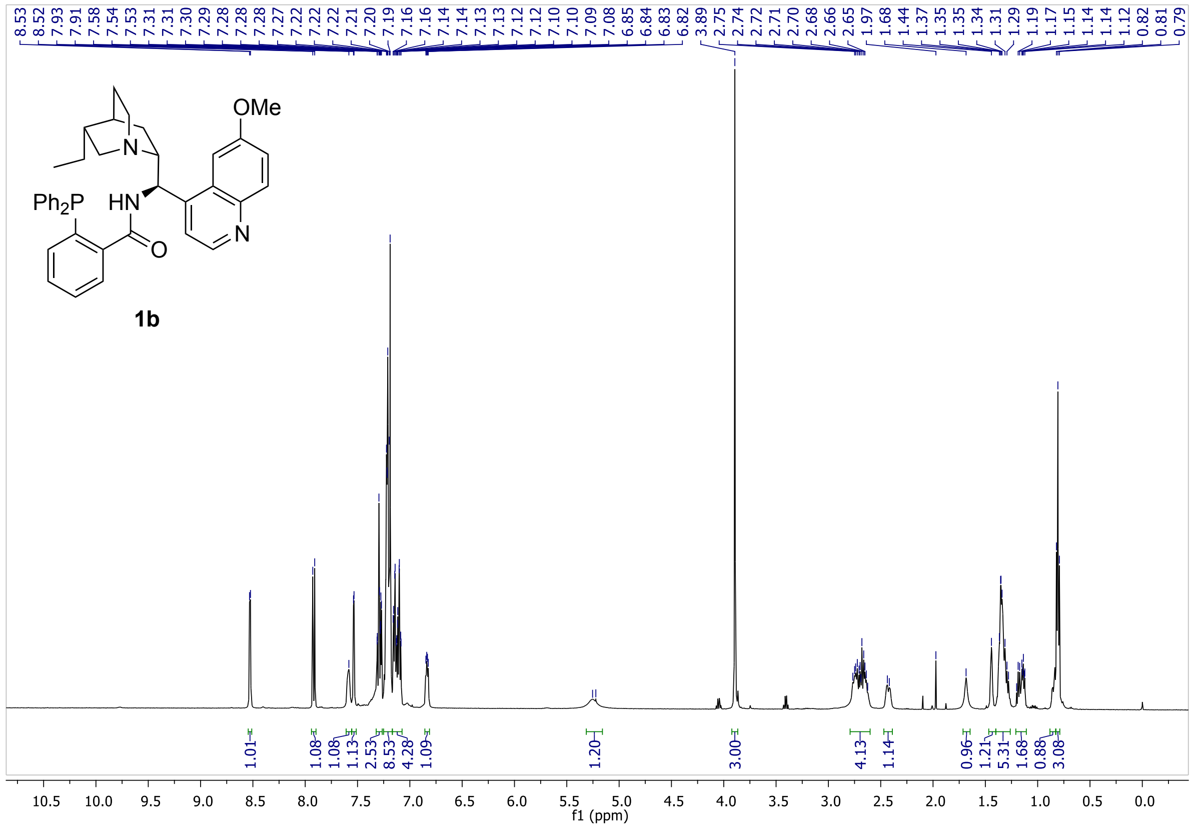

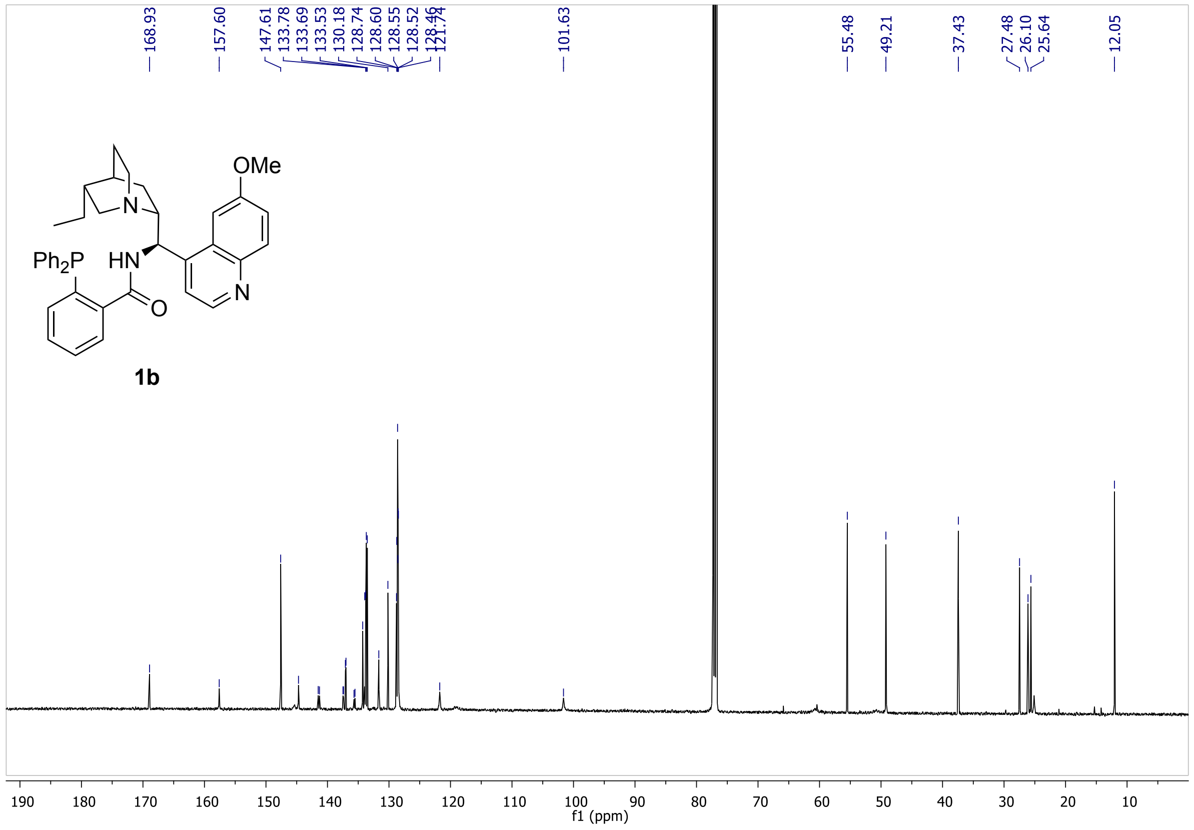

4 ^{1}H and ^{13}C-NMR spectra and HPLC traces of Oxazolines and hydrolyzed derivates

4.1 ^{1}H and ^{13}C-NMR spectra and HPLC traces of compound 4a

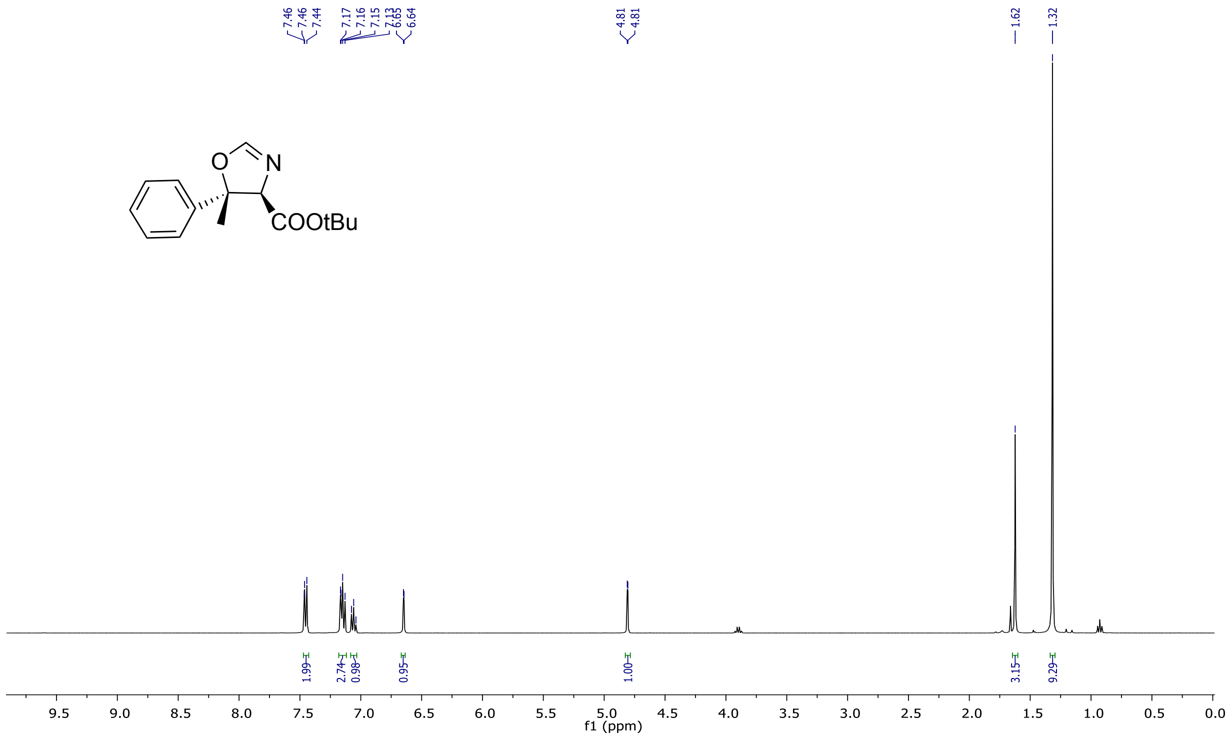

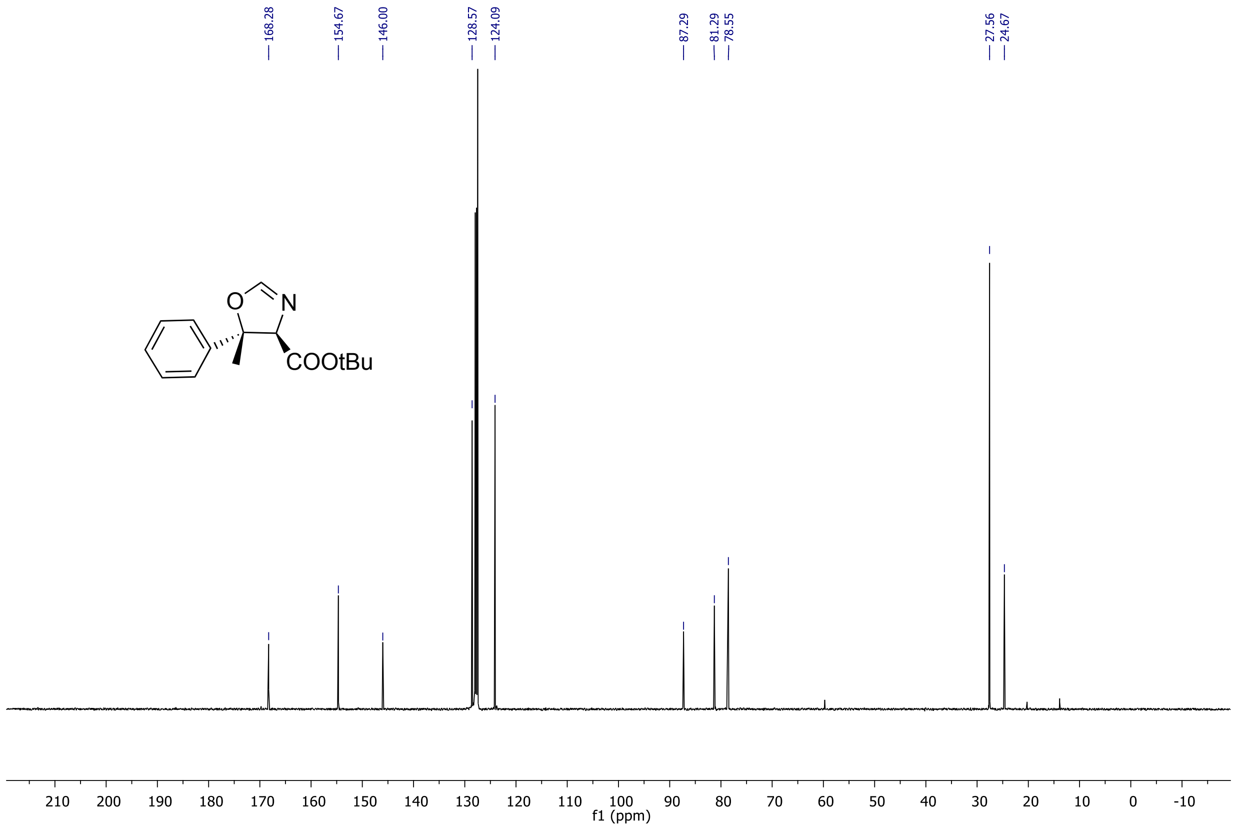

HPLC traces of racemic compound

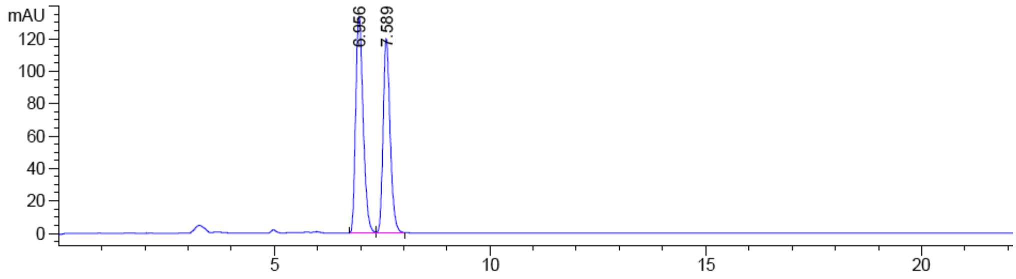

Peak #	RetTime [min]	Type	Width [min]	Area [mAU*s]	Height [mAU]	Area %
1	6.956	BV	0.1891	1612.92114	133.34767	54.3630
2	7.589	VB	0.1740	1354.02734	119.70426	45.6370
Totals :				2966.94849	253.05193	

HPLC traces of **4a**

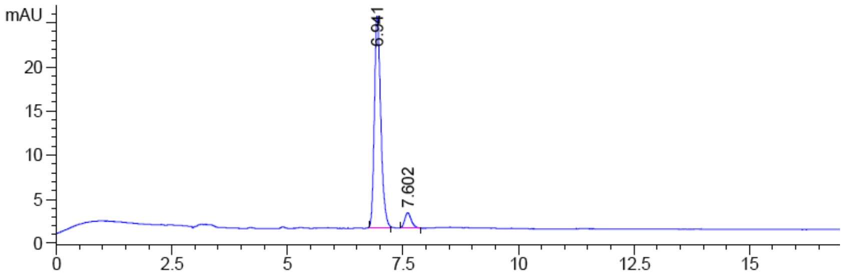

Peak #	RetTime [min]	Type	Width [min]	Area [mAU*s]	Height [mAU]	Area %
1	6.941	MM	0.1629	4864.88232	497.61331	93.5144
2	7.603	MM	0.1569	337.40033	35.83559	6.4856
Totals :				5202.28265	533.44890	

4.2 ^{1}H and ^{13}C-NMR spectra and HPLC traces of compound 4b

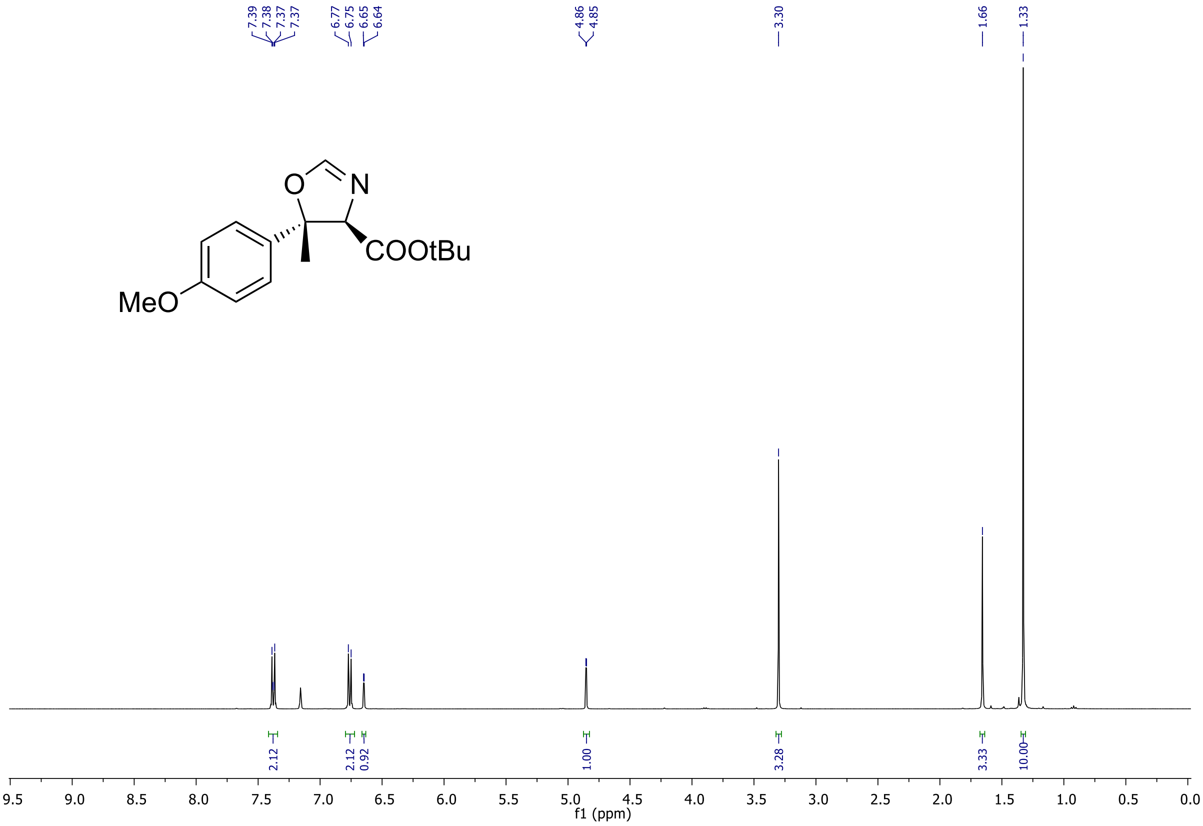

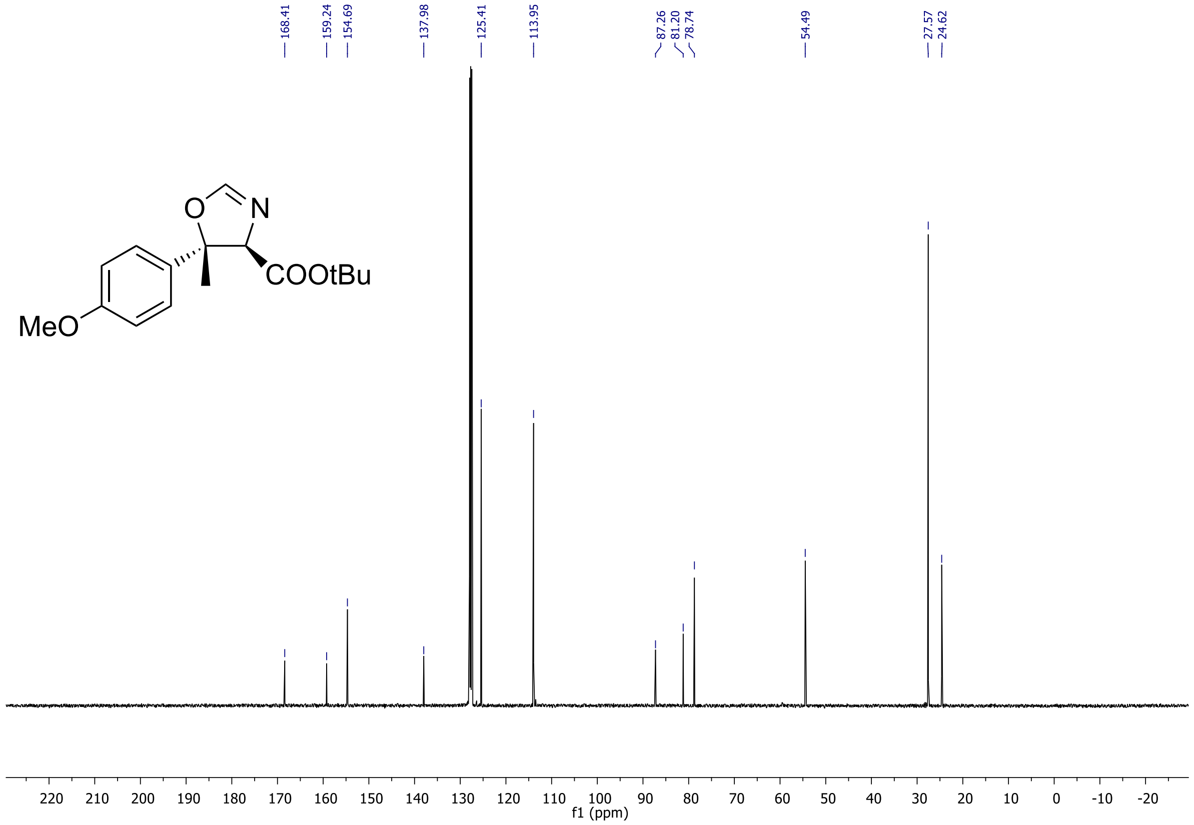

HPLC traces of racemic compound

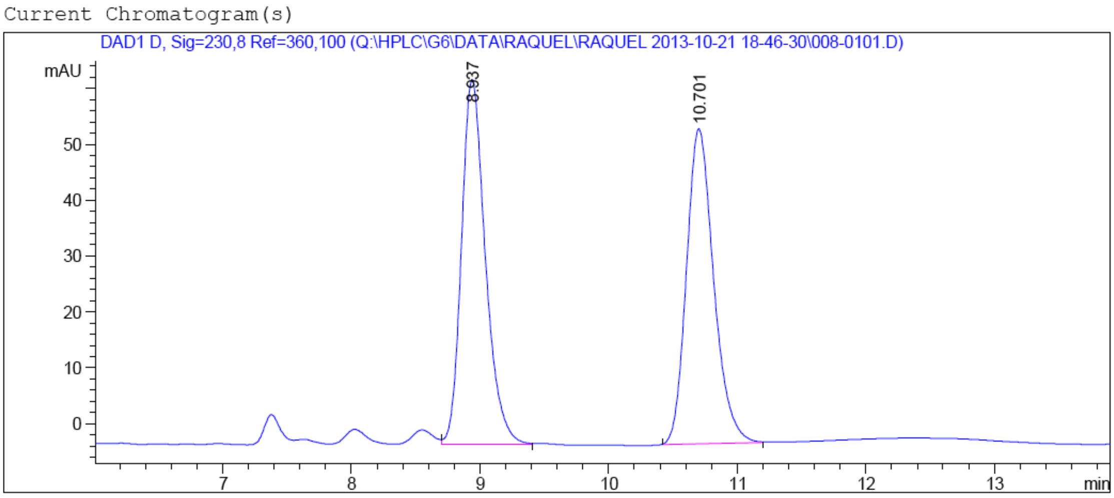

Peak #	RetTime [min]	Type	Width [min]	Area [mAU*s]	Height [mAU]	Area %
1	8.937	VB	0.1968	843.94507	65.30347	50.4431
2	10.701	BB	0.2256	829.11786	56.35551	49.5569

HPLC traces of **4b**

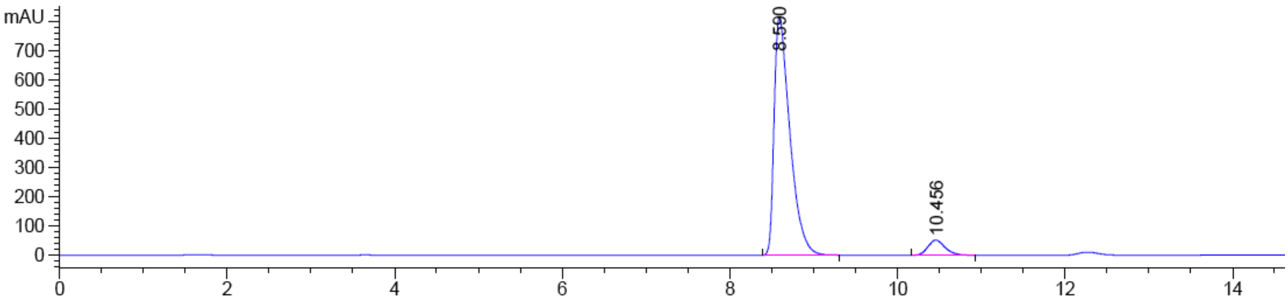

Peak #	RetTime [min]	Type	Width [min]	Area [mAU*s]	Height [mAU]	Area %
1	8.590	BB	0.1997	1.06922e4	811.62561	93.5169
2	10.456	BB	0.2199	741.23425	51.50051	6.4831

4.3 ^{1}H and ^{13}C-NMR spectra and HPLC traces of compound 4c

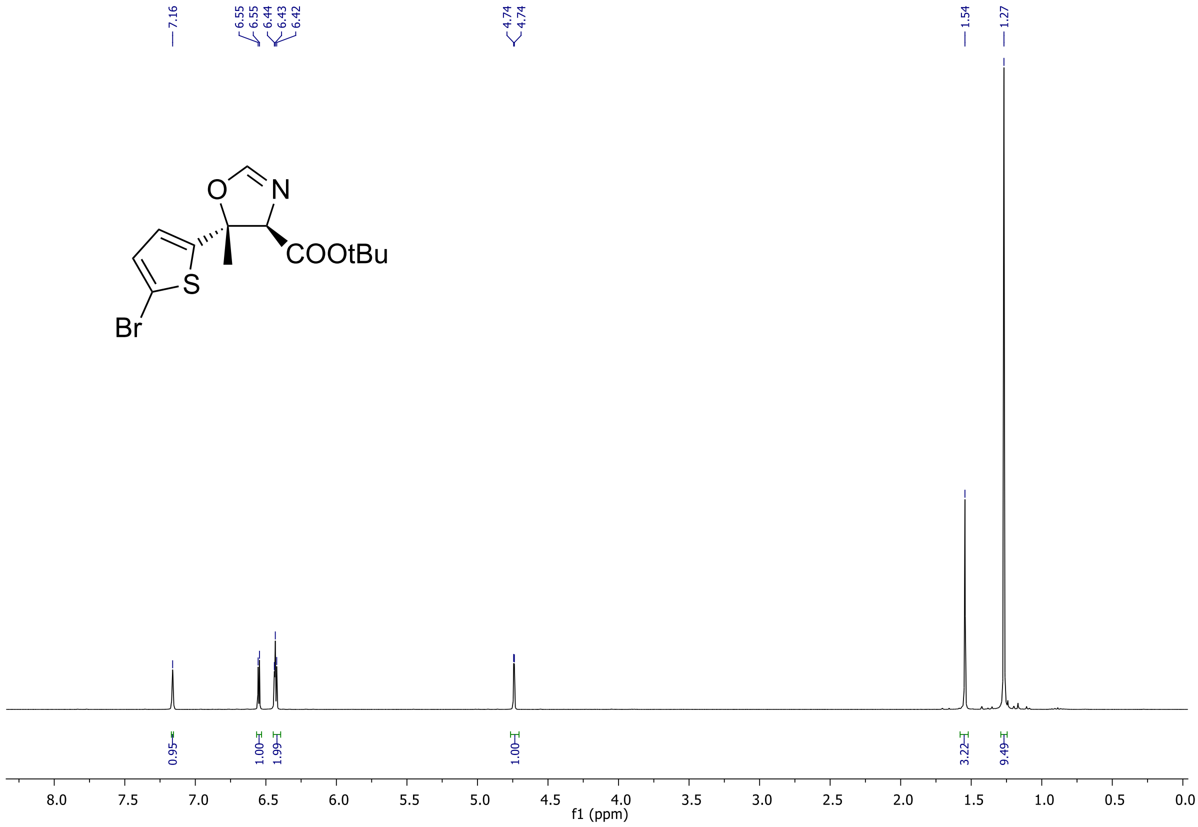

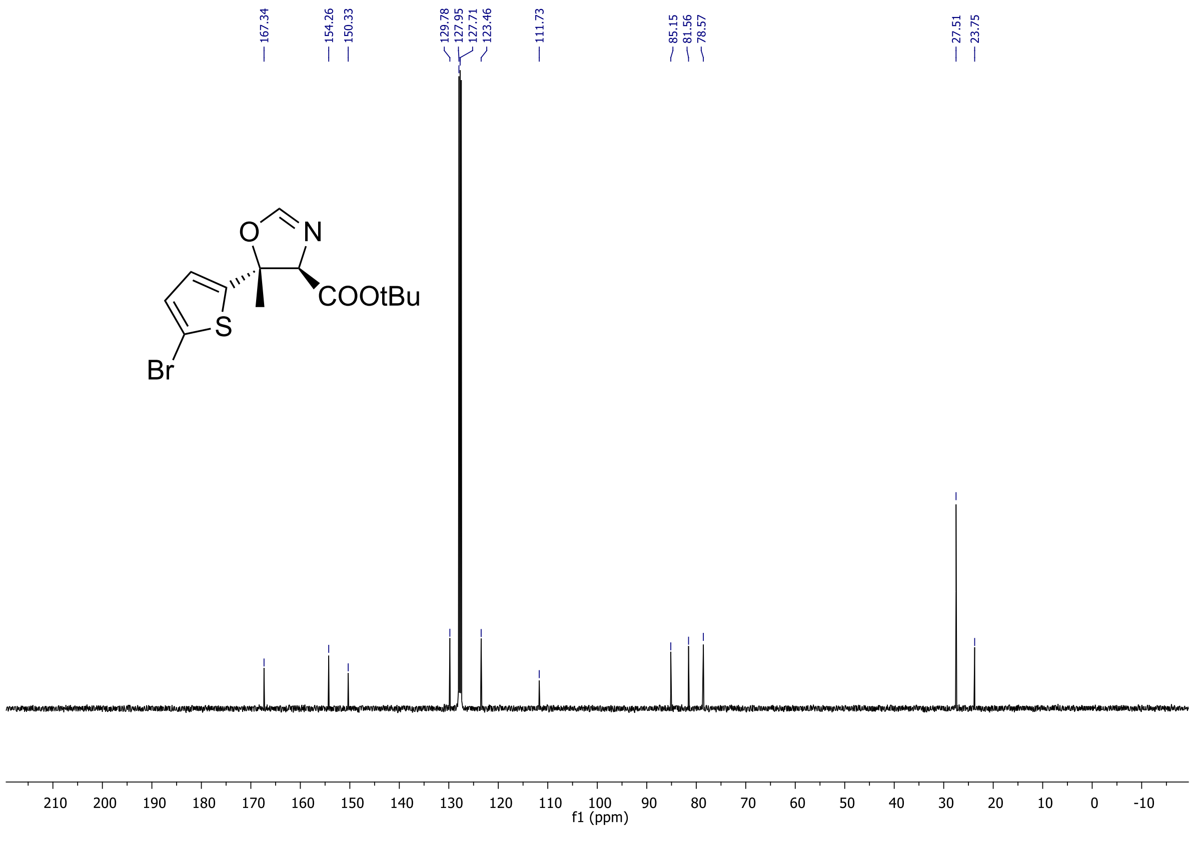

HPLC traces of racemic compound

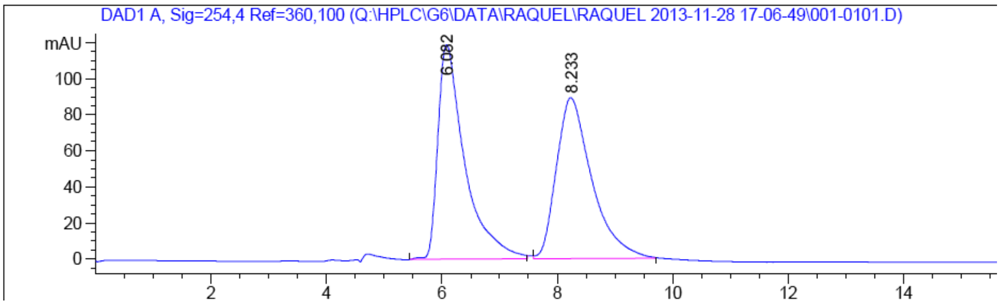

Signal 4: DAD1 D, Sig=230,16 Ref=360,100

Peak #	RetTime [min]	Type	Width [min]	Area [mAU*s]	Height [mAU]	Area %
1	6.197	VB	0.3424	3995.90552	168.66512	49.4806
2	8.418	BB	0.4214	4079.79028	142.75082	50.5194

HPLC traces of **4c**

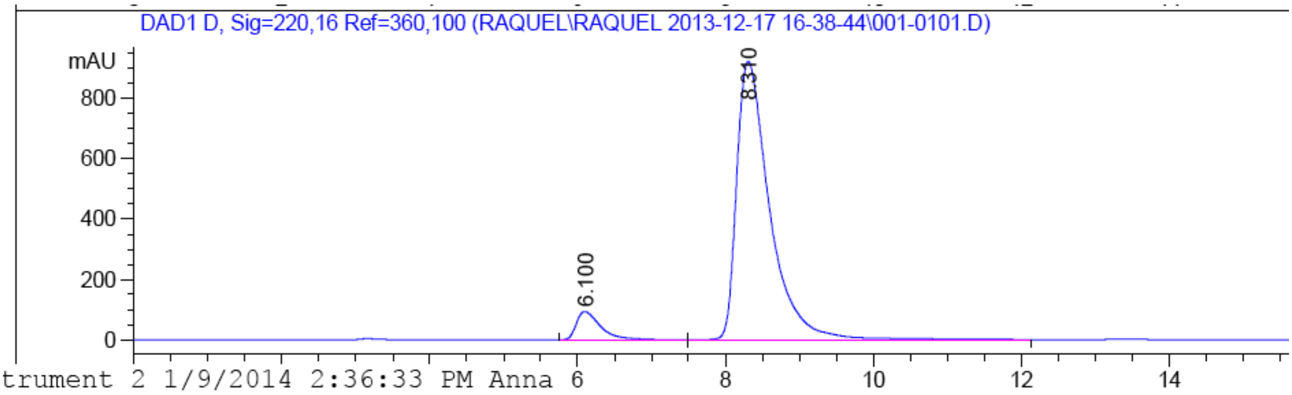

trument 2 1/9/2014 2:36:33 PM Anna

Peak #	RetTime [min]	Type	Width [min]	Area [mAU*s]	Height [mAU]	Area %
1	6.100	VV	0.3556	2183.53760	93.80459	7.0644
2	8.310	VB	0.4724	2.87256e4	919.58276	92.9356

Totals : 3.09091e4 1013.38735

4.4 ^{1}H and ^{13}C-NMR spectra and HPLC traces of compound 4d

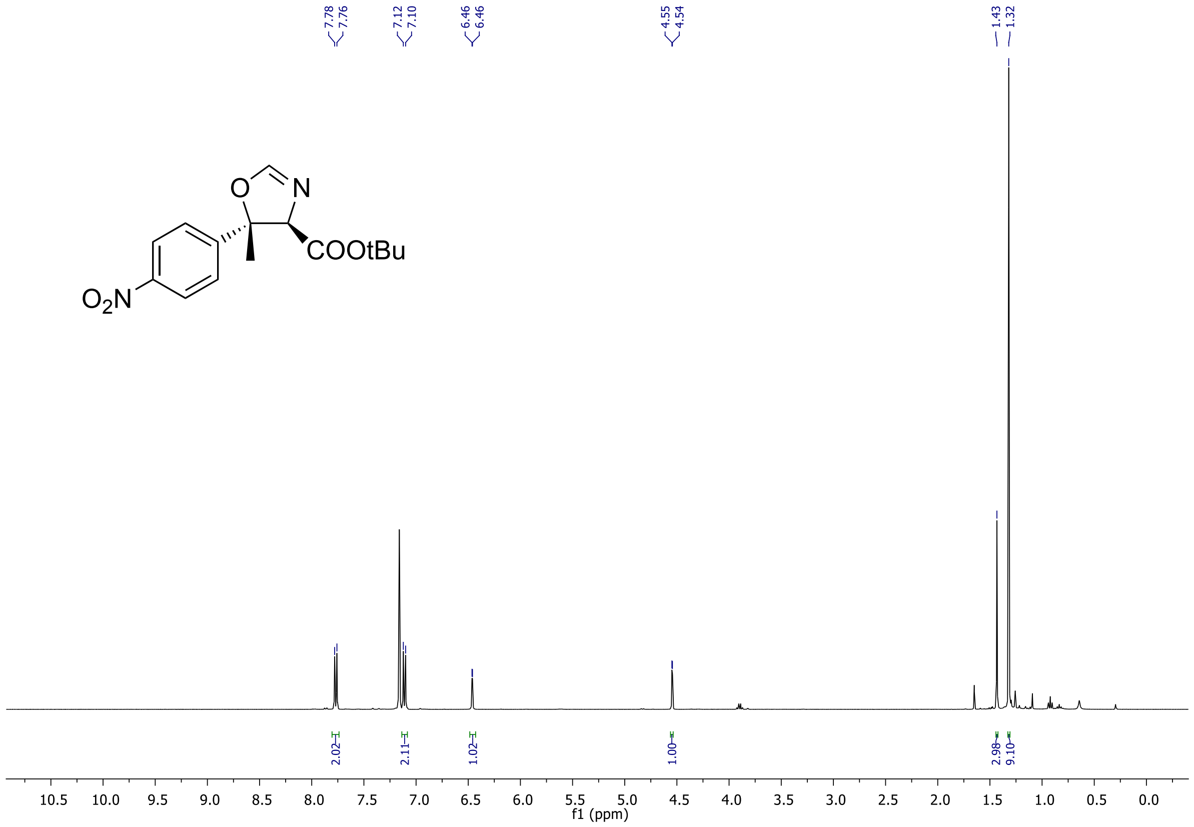

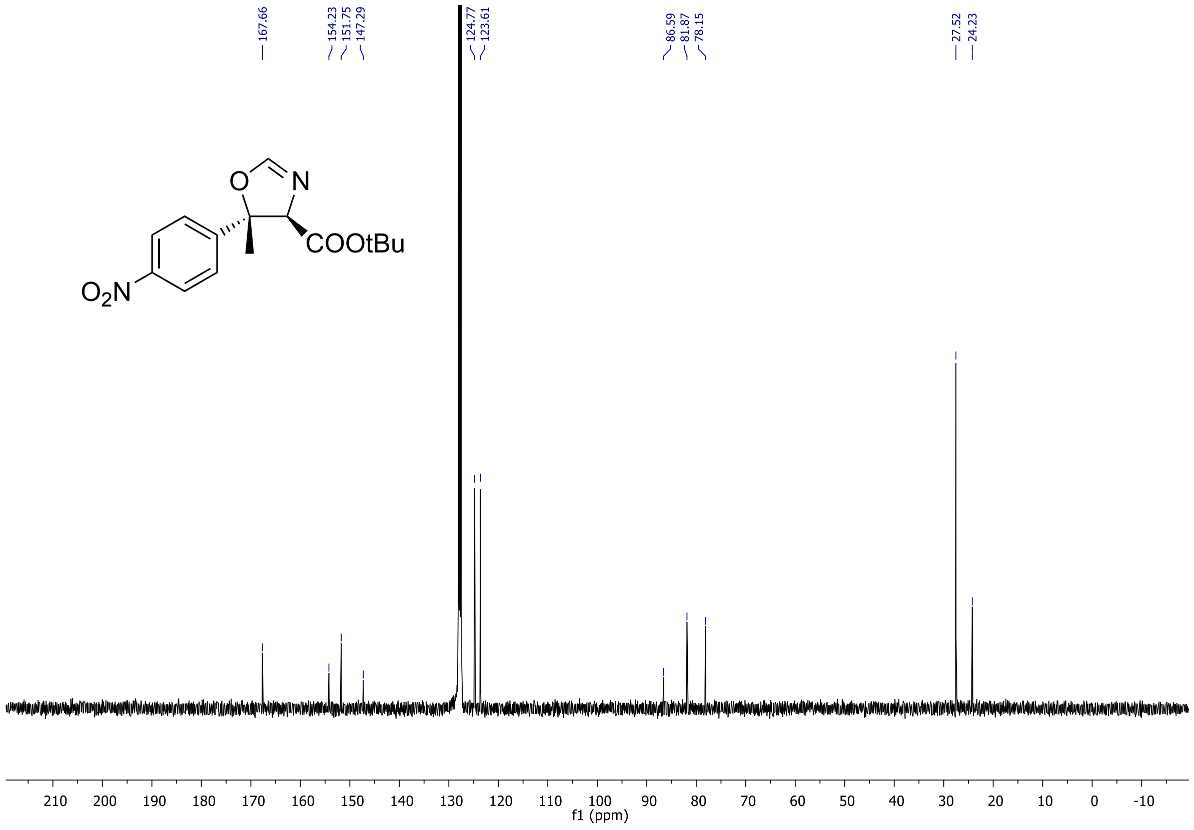

HPLC traces of racemic compound

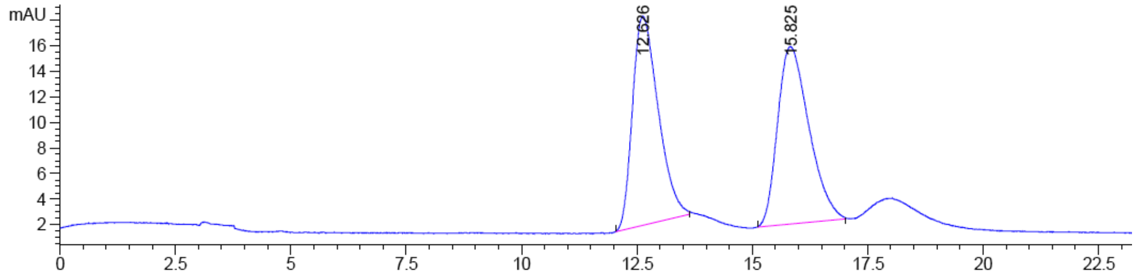

Peak #	RetTime [min]	Type	Width [min]	Area [mAU*s]	Height [mAU]	Area %
1	12.626	BB	0.5037	693.29431	17.94449	49.2240
2	15.825	BB	0.5656	715.15344	15.20797	50.7760

HPLC traces of **4d**

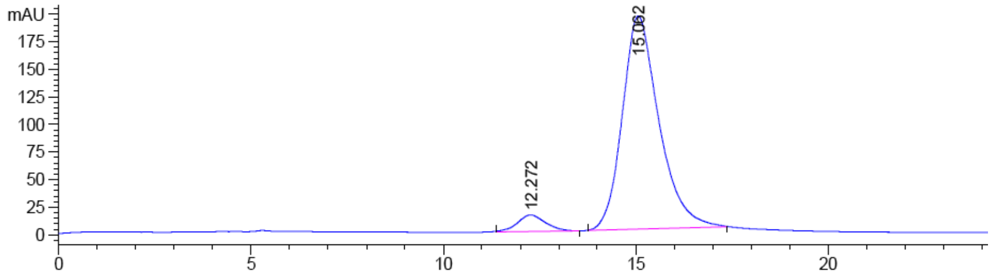

Signal 3: DAD1 C, Sig=210,8 Ref=360,100

Peak #	RetTime [min]	Type	Width [min]	Area [mAU*s]	Height [mAU]	Area %
1	12.276	BB	0.6423	622.91516	12.74217	5.3785
2	15.062	BB	0.9564	1.09587e4	166.38773	94.6215

4.5 ^{1}H and ^{13}C-NMR spectra and HPLC traces of compound 4e

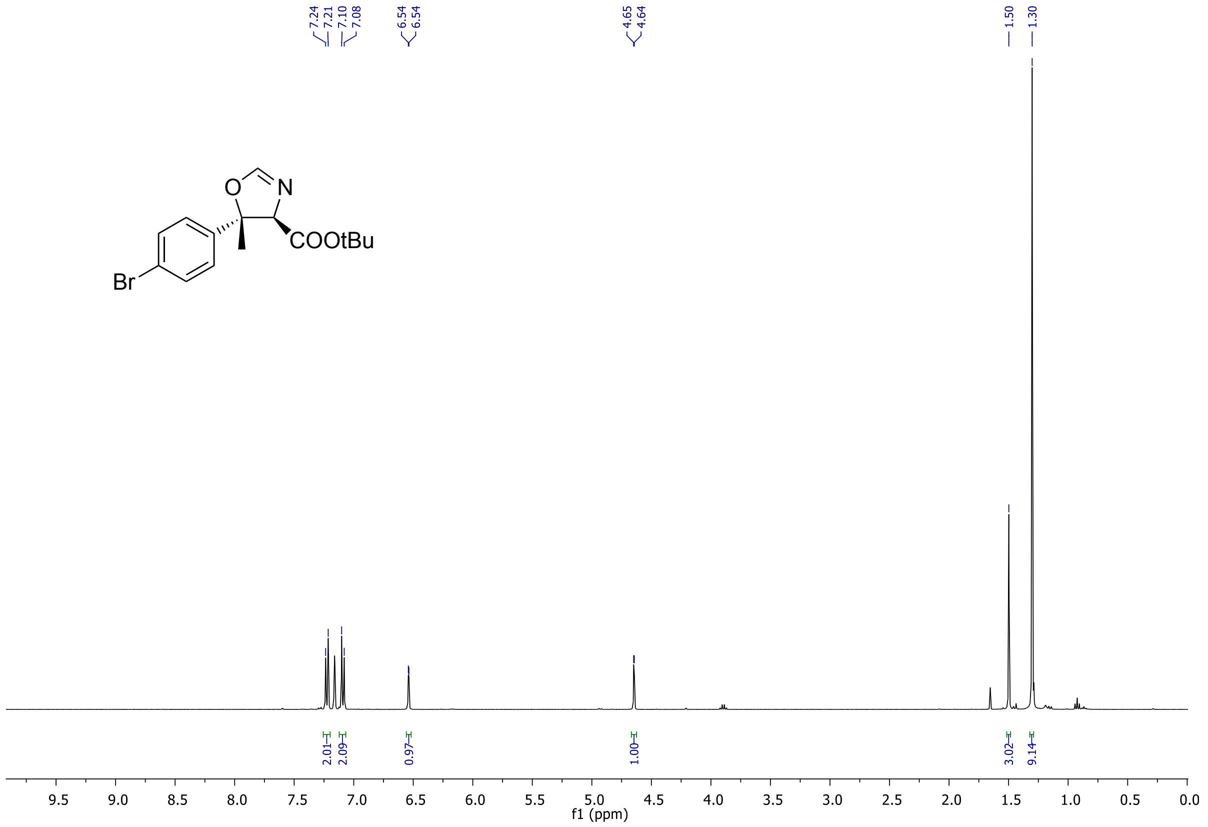

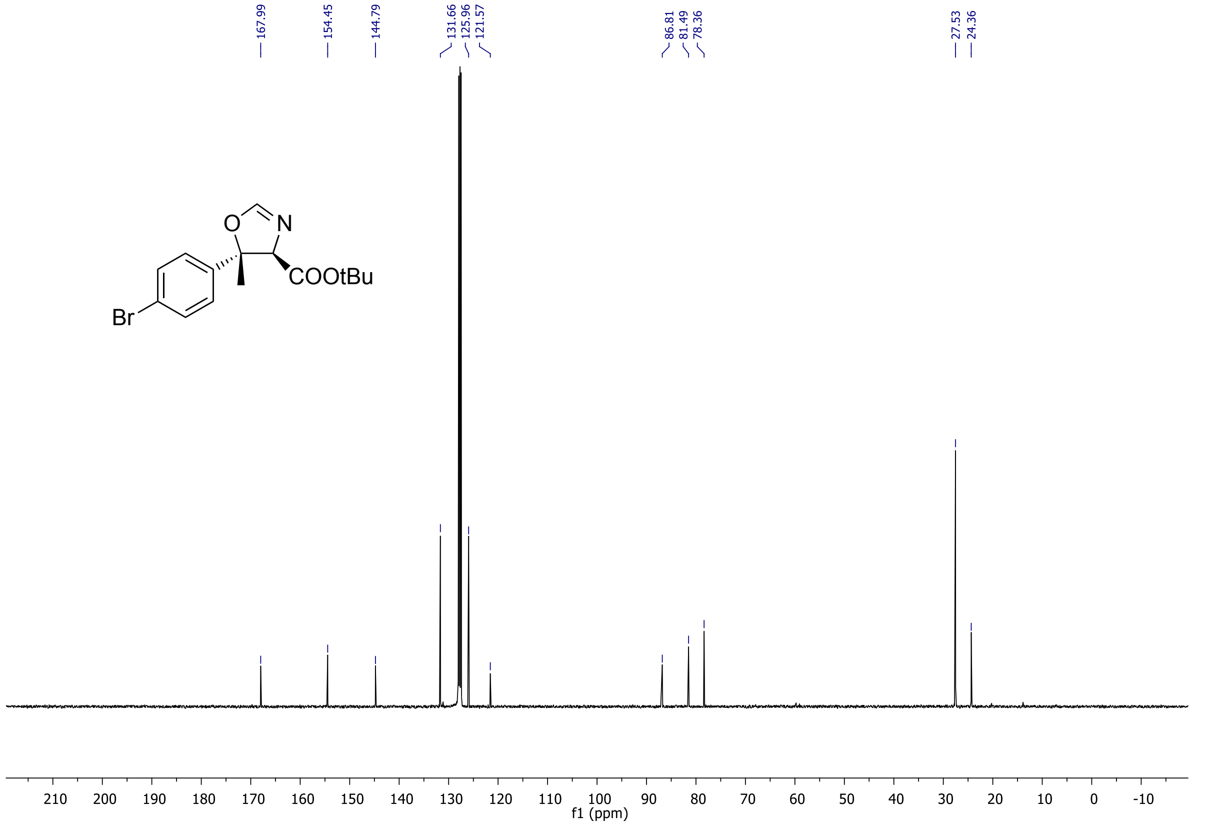

HPLC traces of racemic compound

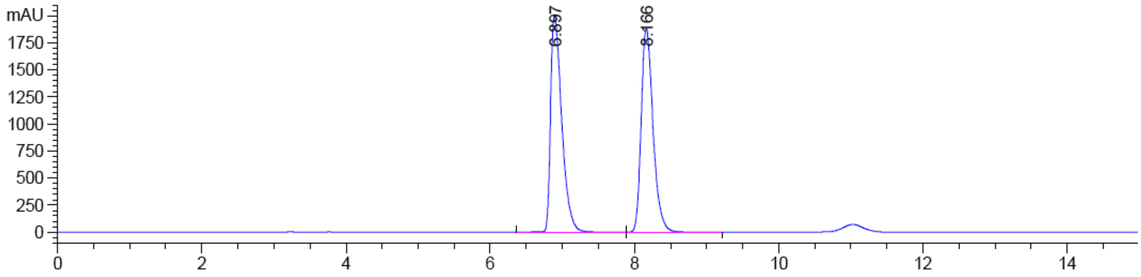

Peak #	RetTime [min]	Type	Width [min]	Area [mAU*s]	Height [mAU]	Area %
1	6.897	VV	0.1667	2.16165e4	1990.69995	50.0425
2	8.166	VB	0.1778	2.15798e4	1881.61841	49.9575

HPLC traces of **4e**

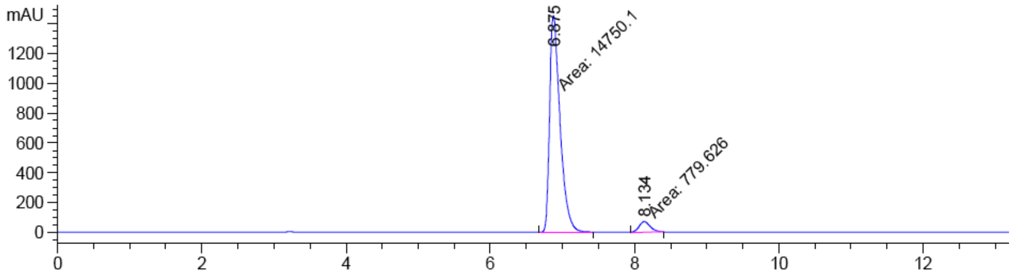

Peak #	RetTime [min]	Type	Width [min]	Area [mAU*s]	Height [mAU]	Area %
1	6.875	MM	0.1684	1.47501e4	1460.06018	94.9798
2	8.134	MM	0.1791	779.62573	72.53478	5.0202

4.6 ¹H and ¹³C-NMR spectra and HPLC traces of compound 4f

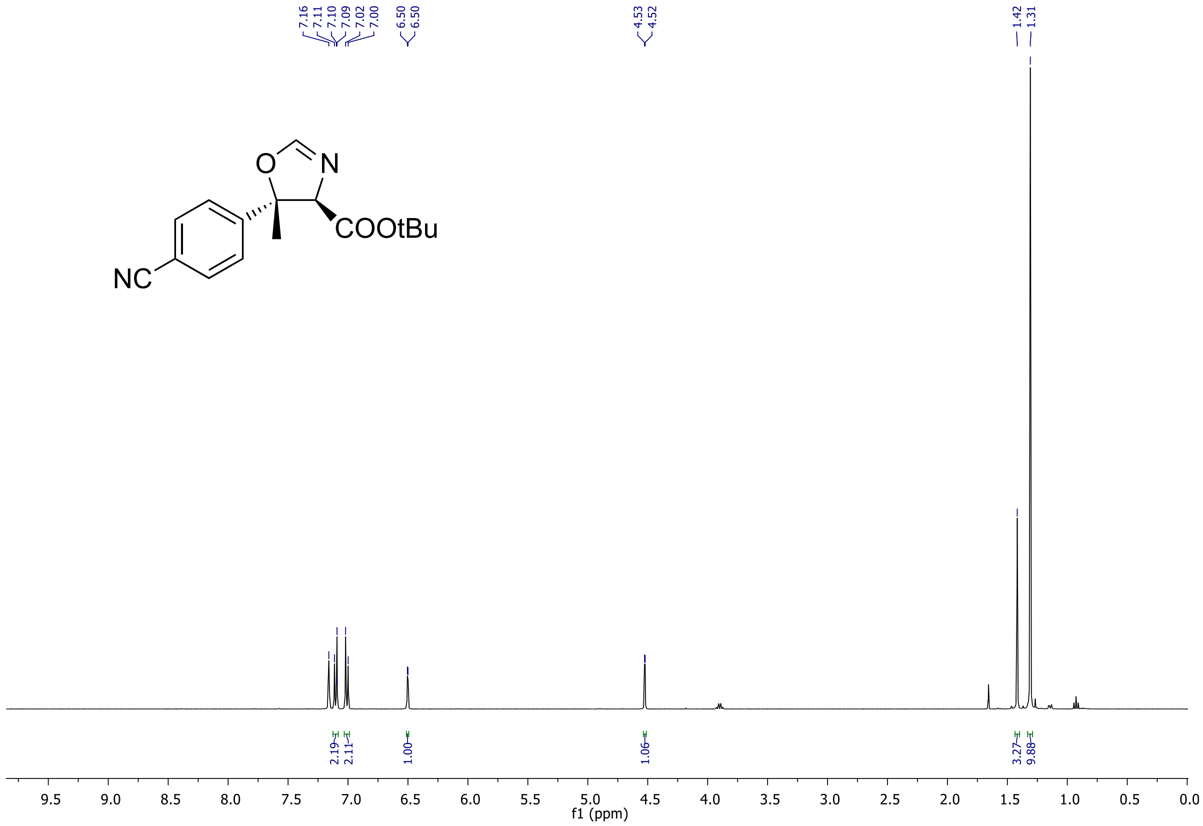

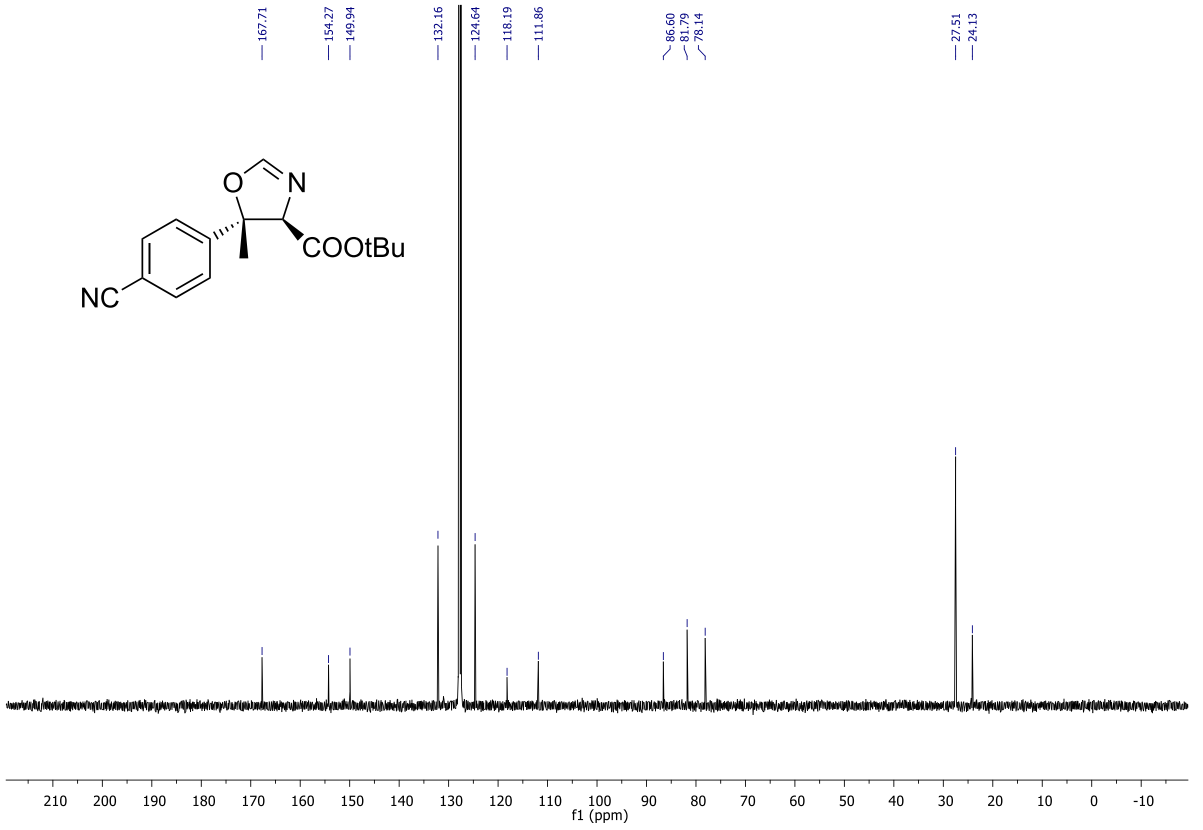

HPLC traces of racemic compound

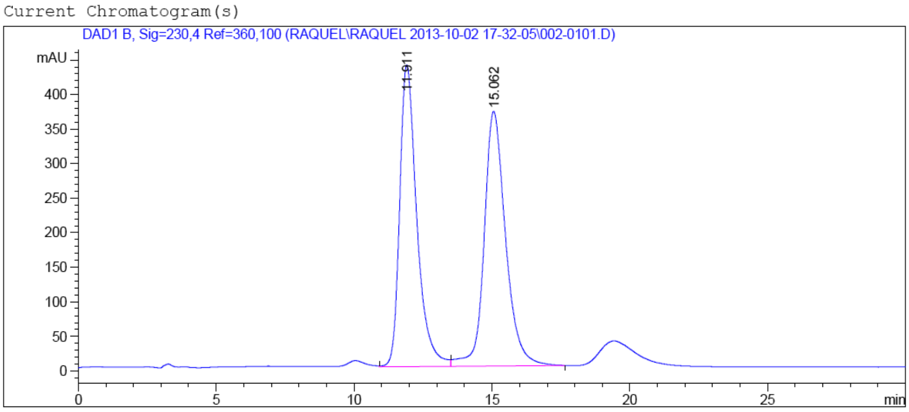

Signal 2: DAD1 B, Sig=230,4 Ref=360,100

Peak #	RetTime [min]	Type	Width [min]	Area [mAU*s]	Height [mAU]	Area %
1	11.911	VB	0.6518	1.88464e4	436.43103	48.5579
2	15.062	BB	0.8082	1.99659e4	368.47104	51.4421

Totals : 3.88123e4 804.90207

HPLC traces of **4f**

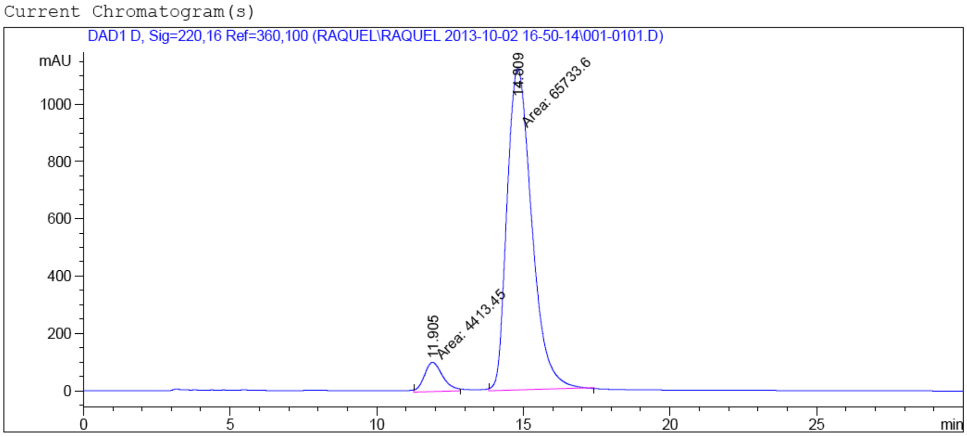

Signal 1: DAD1 A, Sig=254,4 Ref=off

Peak #	RetTime [min]	Type	Width [min]	Area [mAU*s]	Height [mAU]	Area %
1	11.908	BB	0.4533	103.90916	2.71035	4.4185
2	14.837	BB	0.8107	2247.79614	34.98069	95.5815

Totals : 2351.70530 37.69103

4.7 ^{1}H and ^{13}C-NMR spectra and HPLC traces of compound 4g

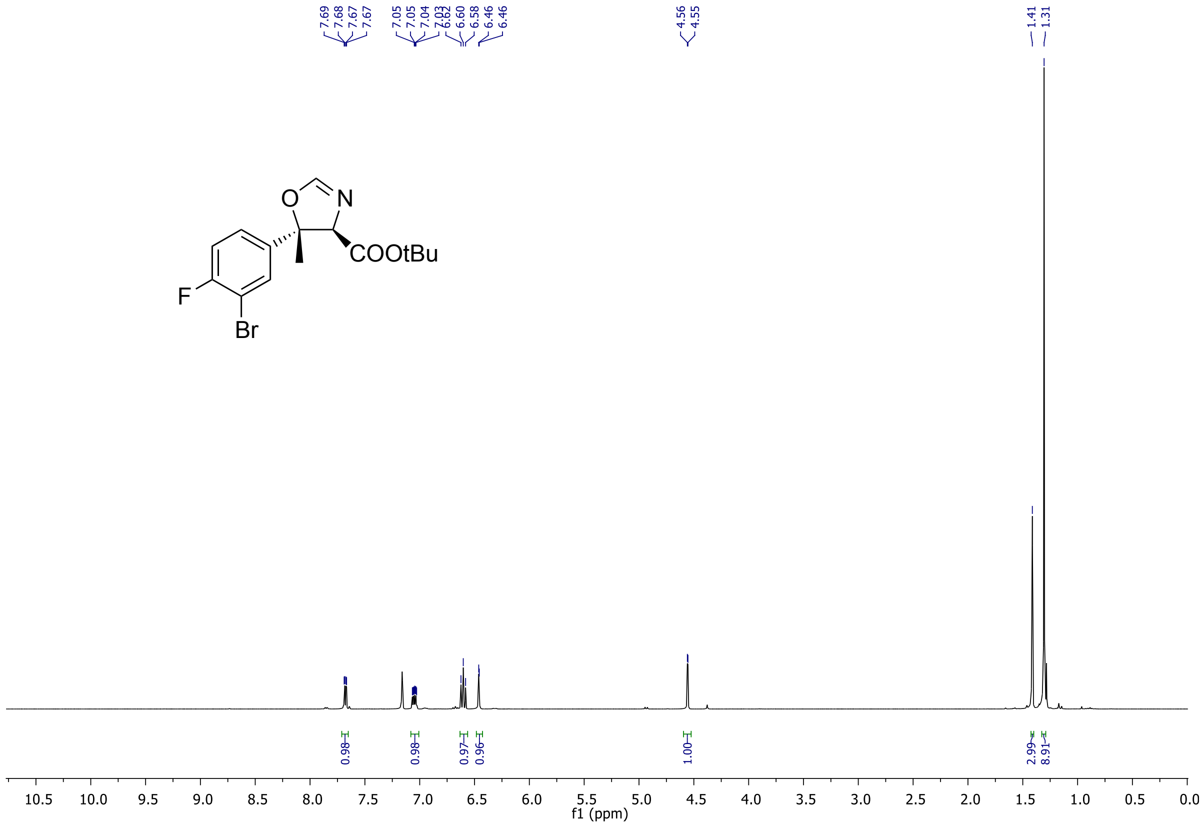

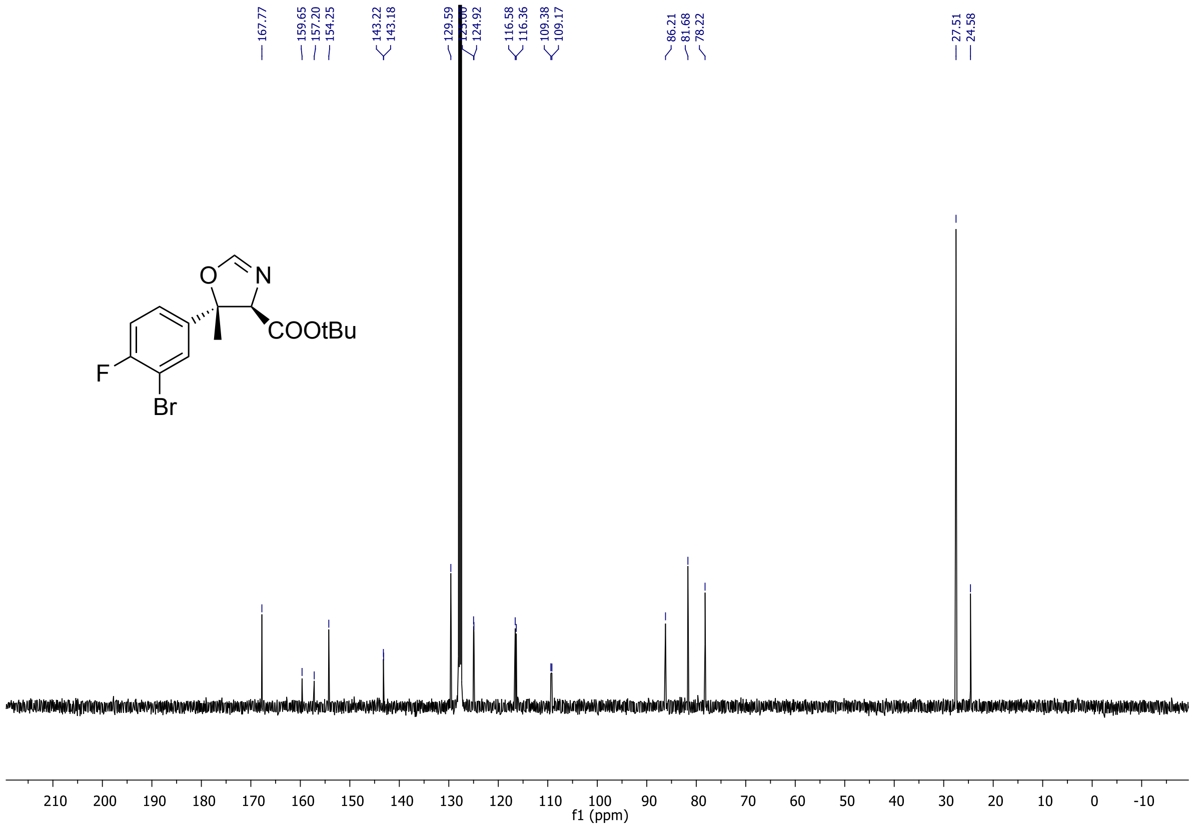

HPLC traces of racemic compound

HPLC traces of racemic compound

Current Chromatogram(s)

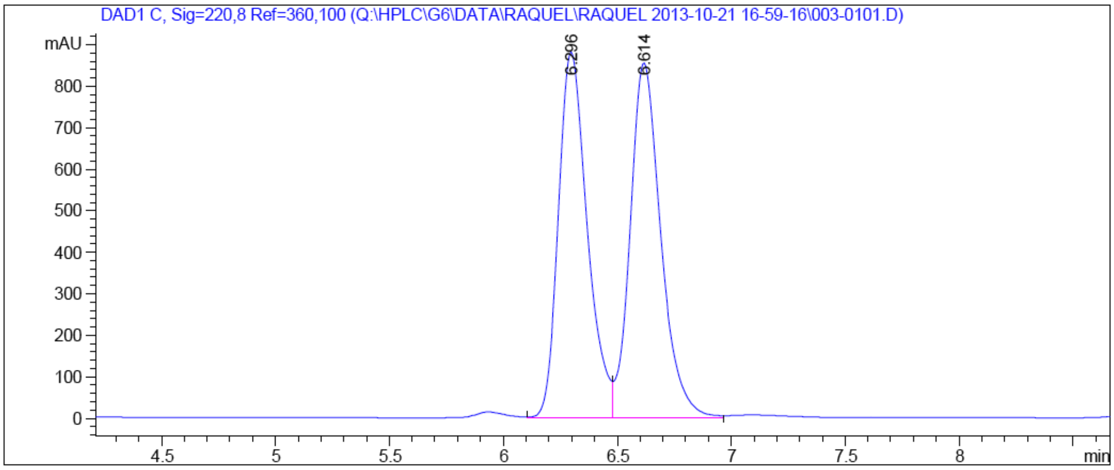

Signal 3: DAD1 C, Sig=220,8 Ref=360,100

Peak #	RetTime [min]	Type	Width [min]	Area [mAU*s]	Height [mAU]	Area %
1	6.296	VV	0.1347	7754.74414	881.13116	49.2245
2	6.614	VV	0.1411	7999.07861	855.15680	50.7755

Totals : 1.57538e4 1736.28796

HPLC traces of **4g**

Current Chromatogram(s)

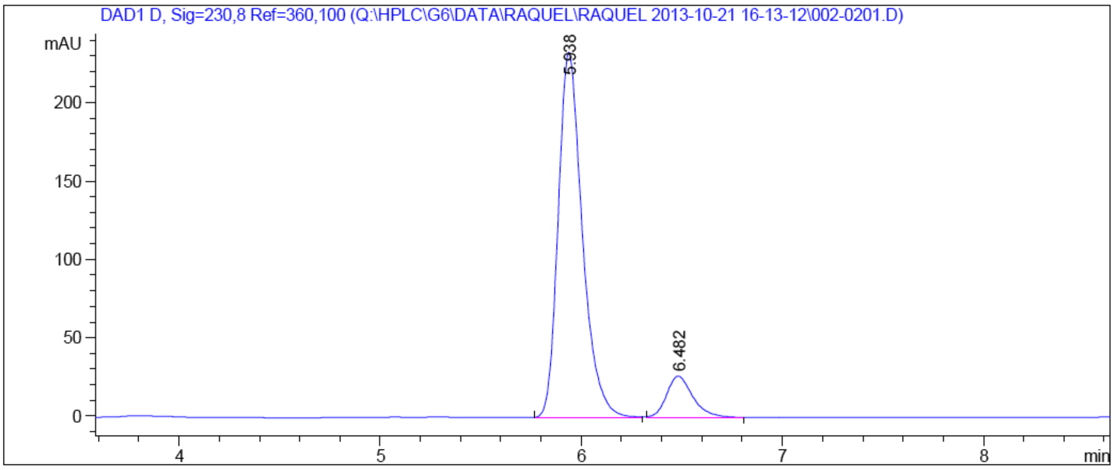

Signal 4: DAD1 D, Sig=230,8 Ref=360,100

Peak #	RetTime [min]	Type	Width [min]	Area [mAU*s]	Height [mAU]	Area %
1	5.938	BB	0.1273	1945.37500	233.21146	89.0379
2	6.482	BB	0.1378	239.50926	26.40556	10.9621

4.8 ^{1}H and ^{13}C-NMR spectra and HPLC traces of compound 4h

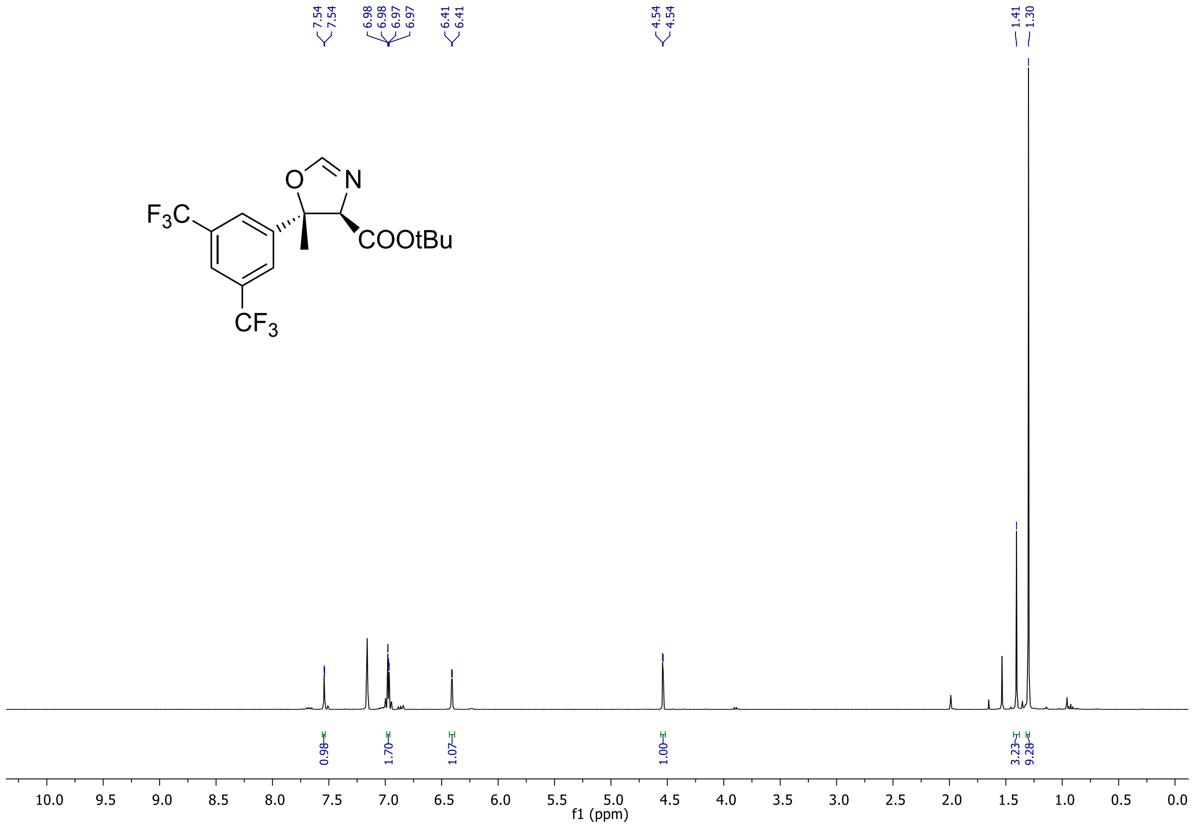

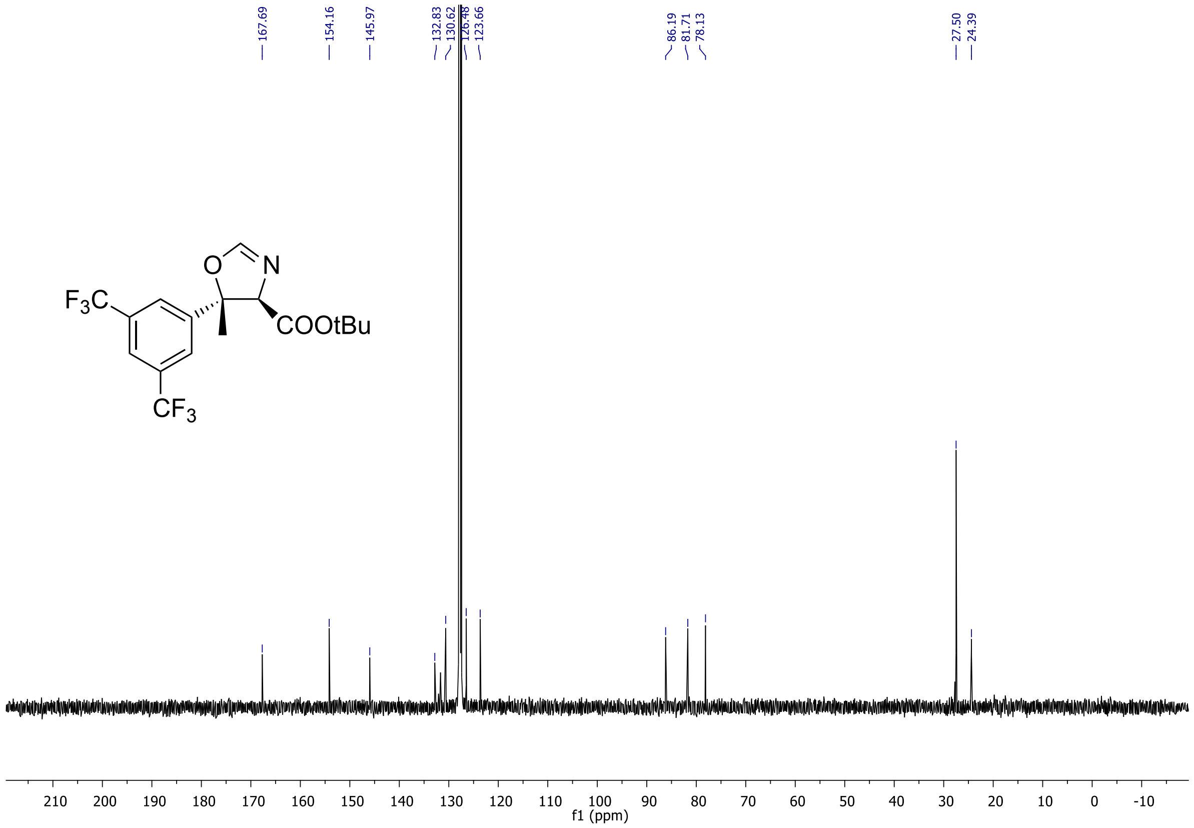

HPLC traces of racemic compound

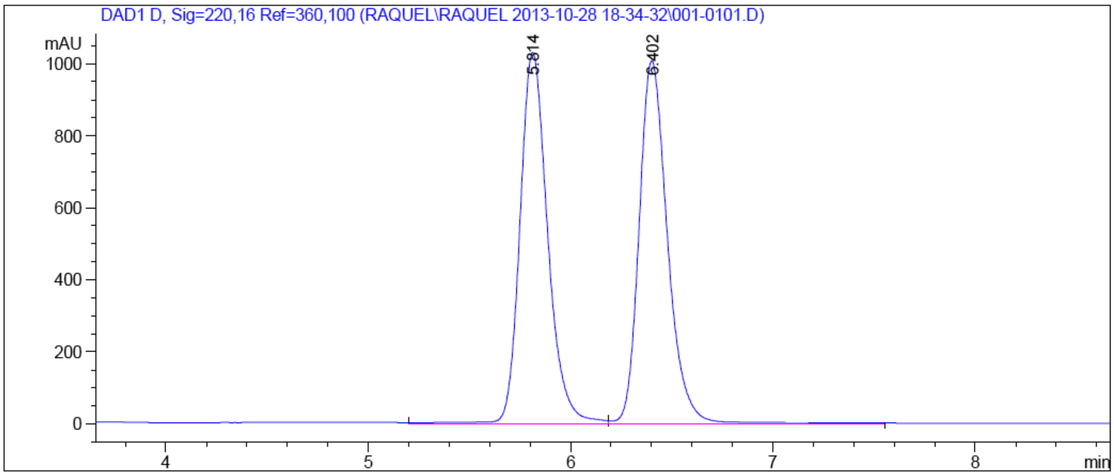

Peak #	RetTime [min]	Type	Width [min]	Area [mAU*s]	Height [mAU]	Area %
1	5.814	VV	0.1510	9761.08789	1025.51343	49.9528
2	6.402	VB	0.1537	9779.53516	1003.34247	50.0472

Totals : 1.95406e4 2028.85590

HPLC traces of **4h**

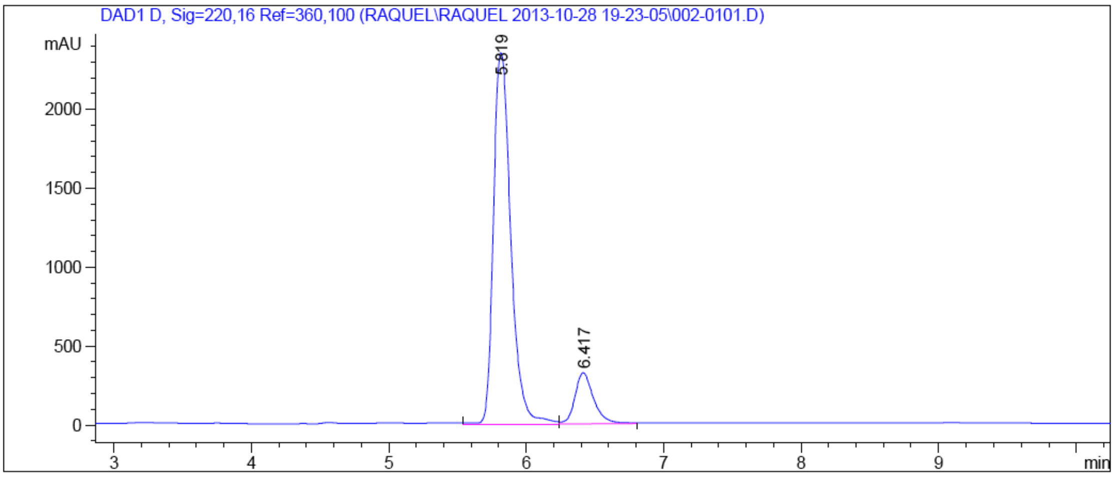

Peak #	RetTime [min]	Type	Width [min]	Area [mAU*s]	Height [mAU]	Area %
1	5.817	BB	0.1214	256.38623	32.01791	87.9653
2	6.414	BB	0.1364	35.07665	3.88100	12.0347

Totals : 291.46288 35.89891

4.9 ^{1}H and ^{13}C-NMR spectra and HPLC traces of compound 4i

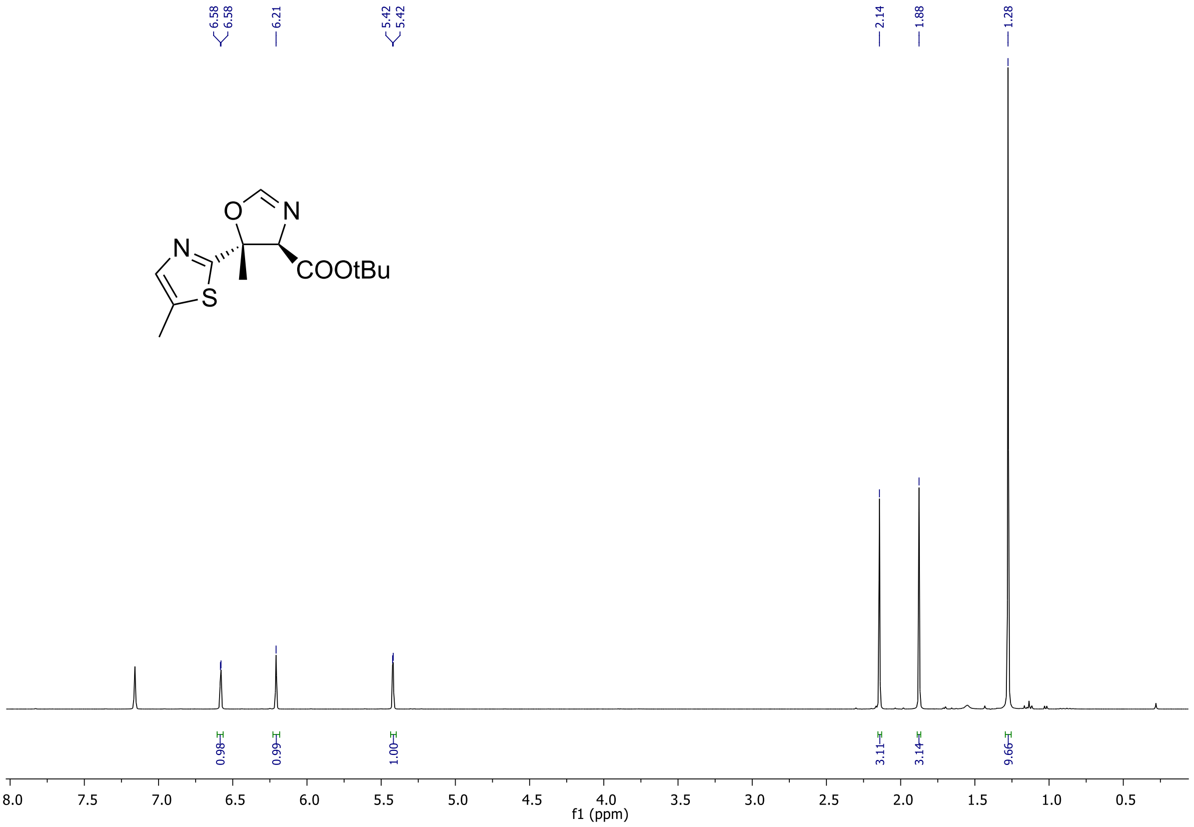

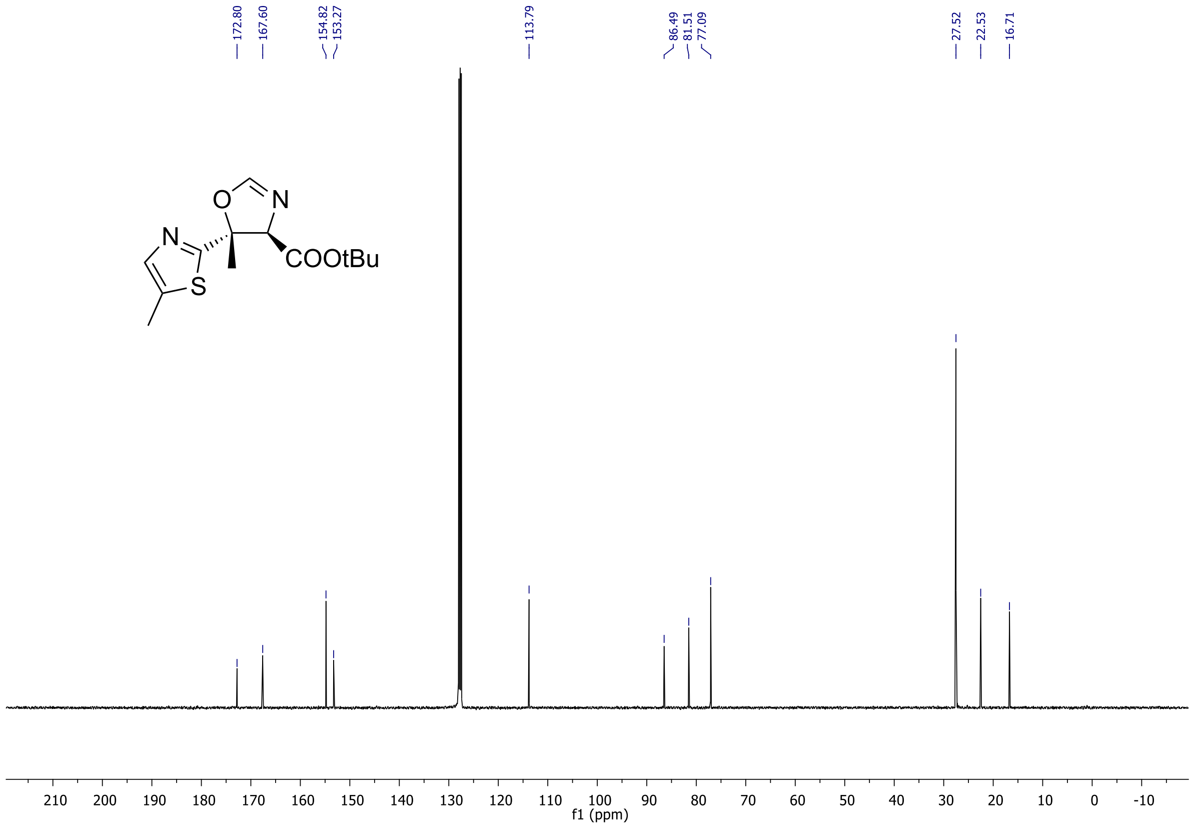

HPLC traces of racemic compound

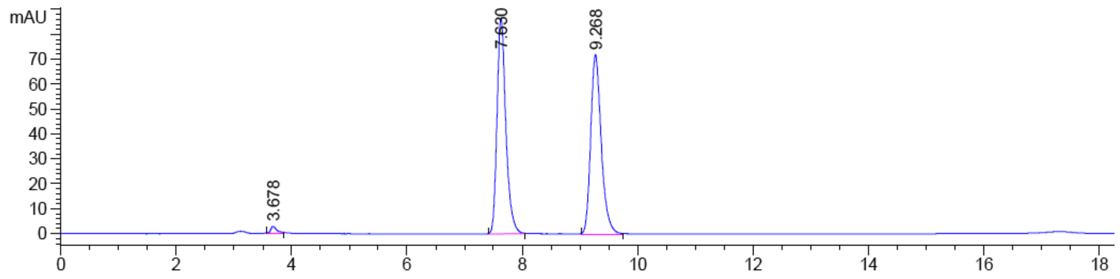

Peak #	RetTime [min]	Type	Width [min]	Area [mAU*s]	Height [mAU]	Area %
1	7.630	BB	0.1623	1141.33044	107.12650	50.2818
2	9.268	BB	0.1930	1128.53552	88.98963	49.7182

HPLC traces of **4i**

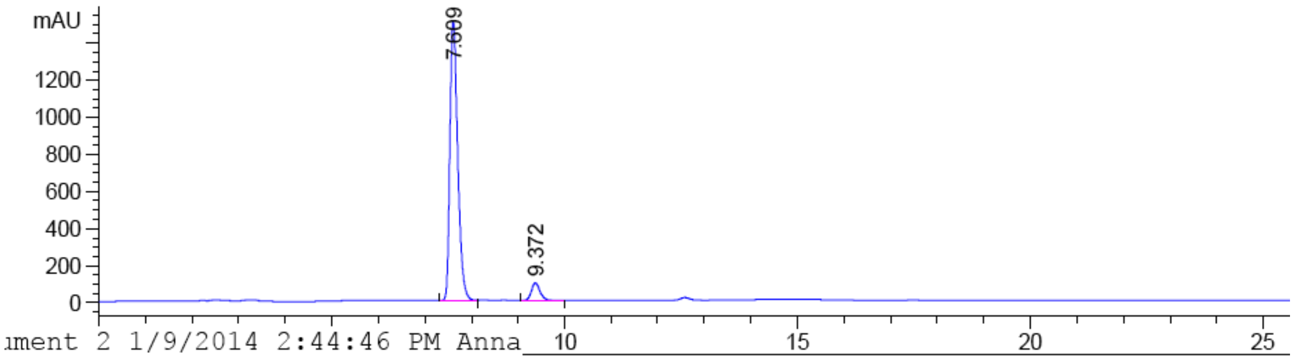

Peak #	RetTime [min]	Type	Width [min]	Area [mAU*s]	Height [mAU]	Area %
1	7.609	VV	0.1848	1.76938e4	1509.44238	93.2254
2	9.372	VB	0.2054	1285.78613	95.33325	6.7746

Totals : 1.89796e4 1604.77563

4.10 ^{1}H and ^{13}C-NMR spectra and HPLC traces of compound 4j

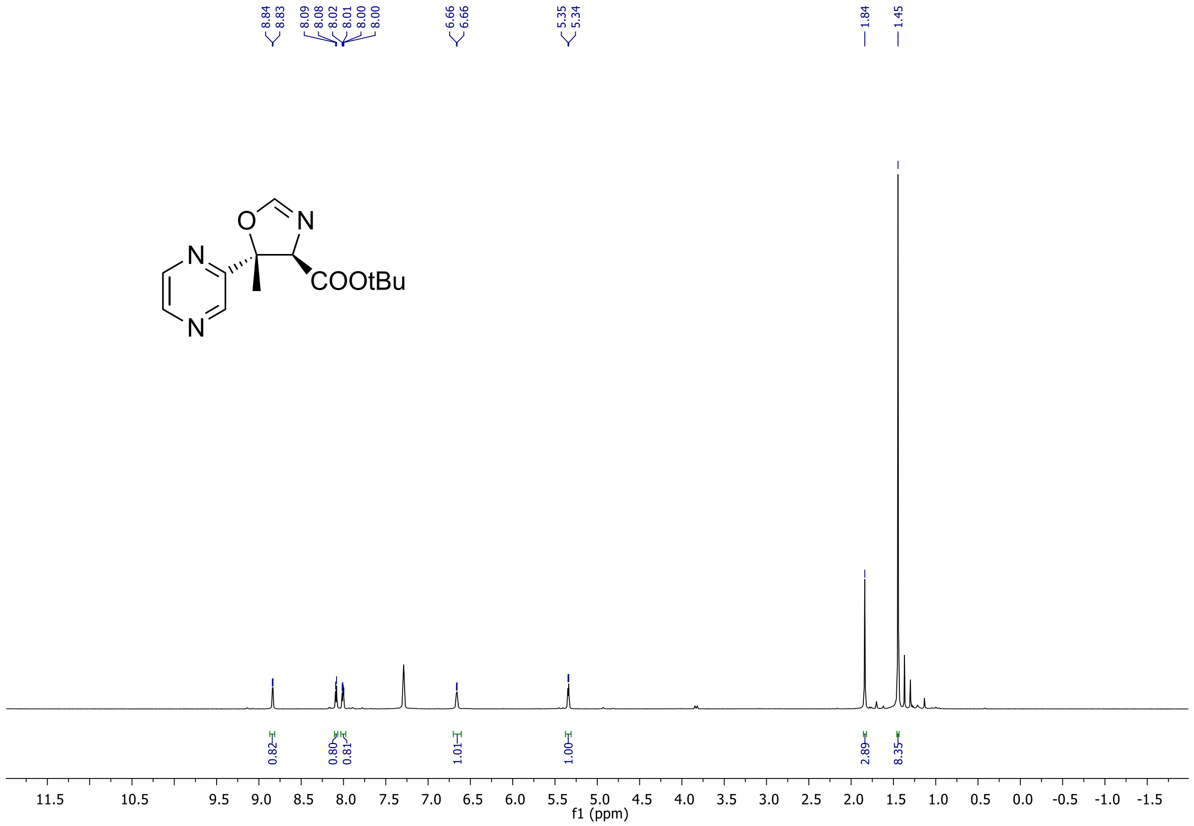

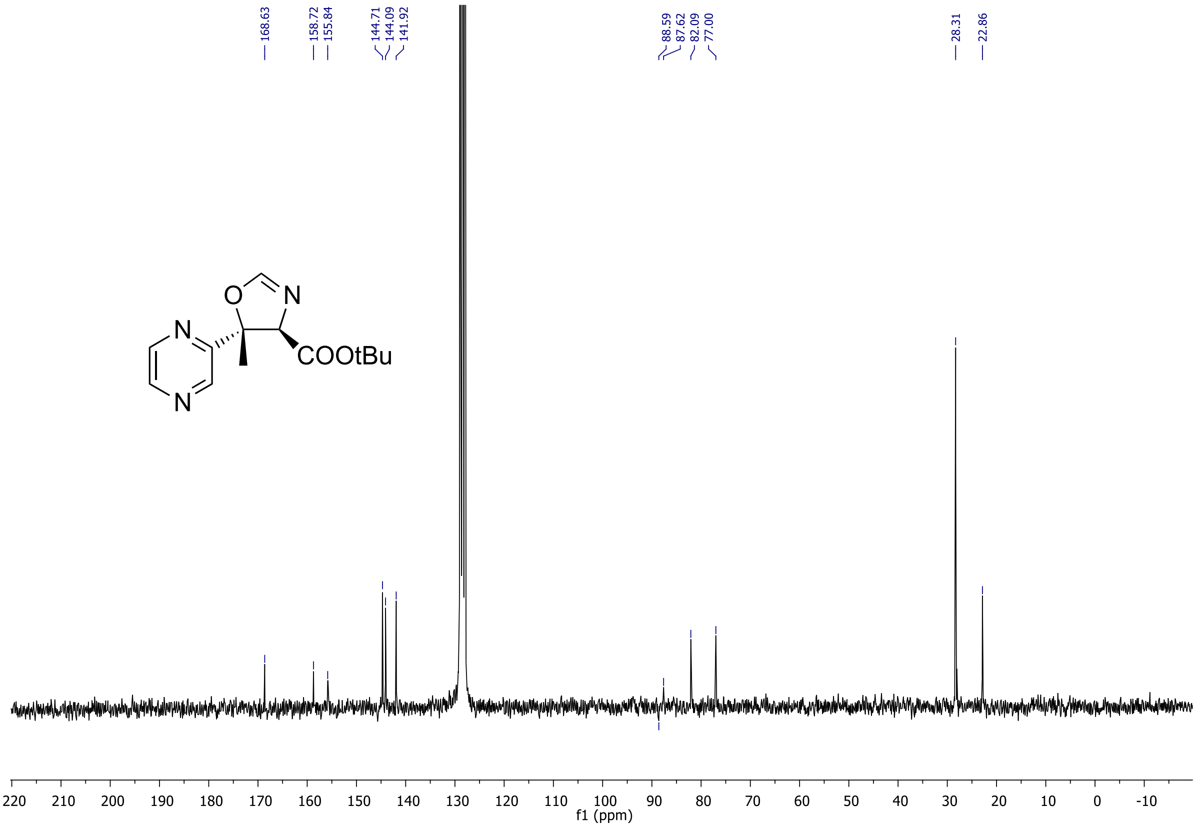

HPLC traces of racemic compound

Additional Info : Peak(s) manually integrated

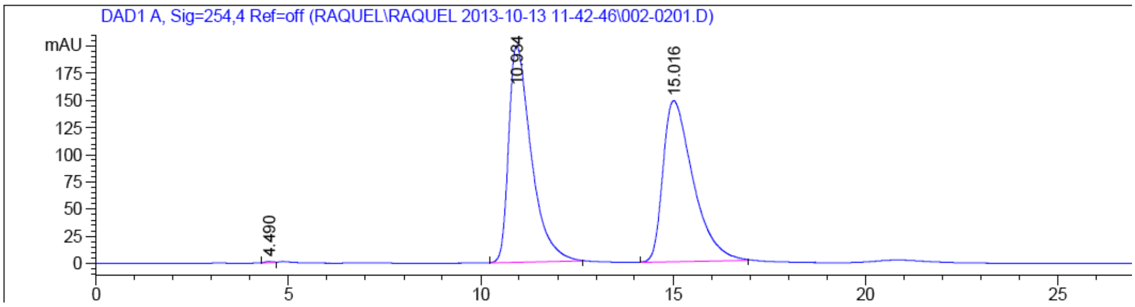

Peak #	RetTime [min]	Type	Width [min]	Area [mAU*s]	Height [mAU]	Area %
1	10.934	MM	0.6850	1564.26111	38.06161	49.6516
2	15.014	MM	0.9278	1586.21387	28.49492	50.3484

Totals : 3150.47498 66.55653

HPLC traces of **4j**

Additional Info : Peak(s) manually integrated

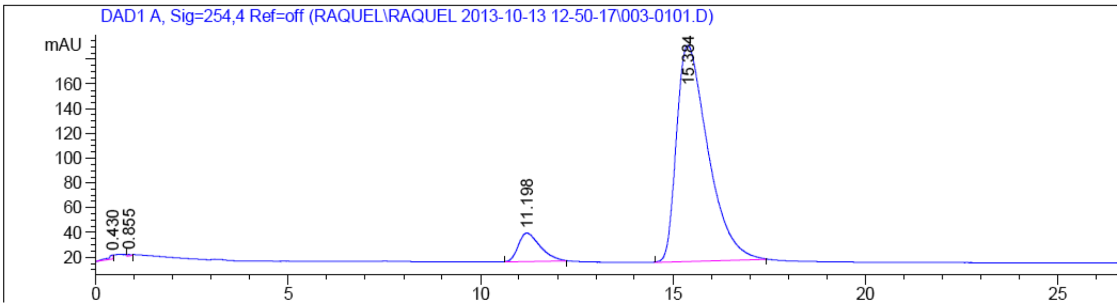

Signal 5: DAD1 E, Sig=240,4 Ref=360,100

Peak #	RetTime [min]	Type	Width [min]	Area [mAU*s]	Height [mAU]	Area %
1	11.203	MM	0.6948	188.61079	4.52434	8.7935
2	15.389	MM	0.9723	1956.27539	33.53420	91.2065

Totals : 2144.88618 38.05854

4.11 ^{1}H and ^{13}C-NMR spectra and HPLC traces of compound 4k

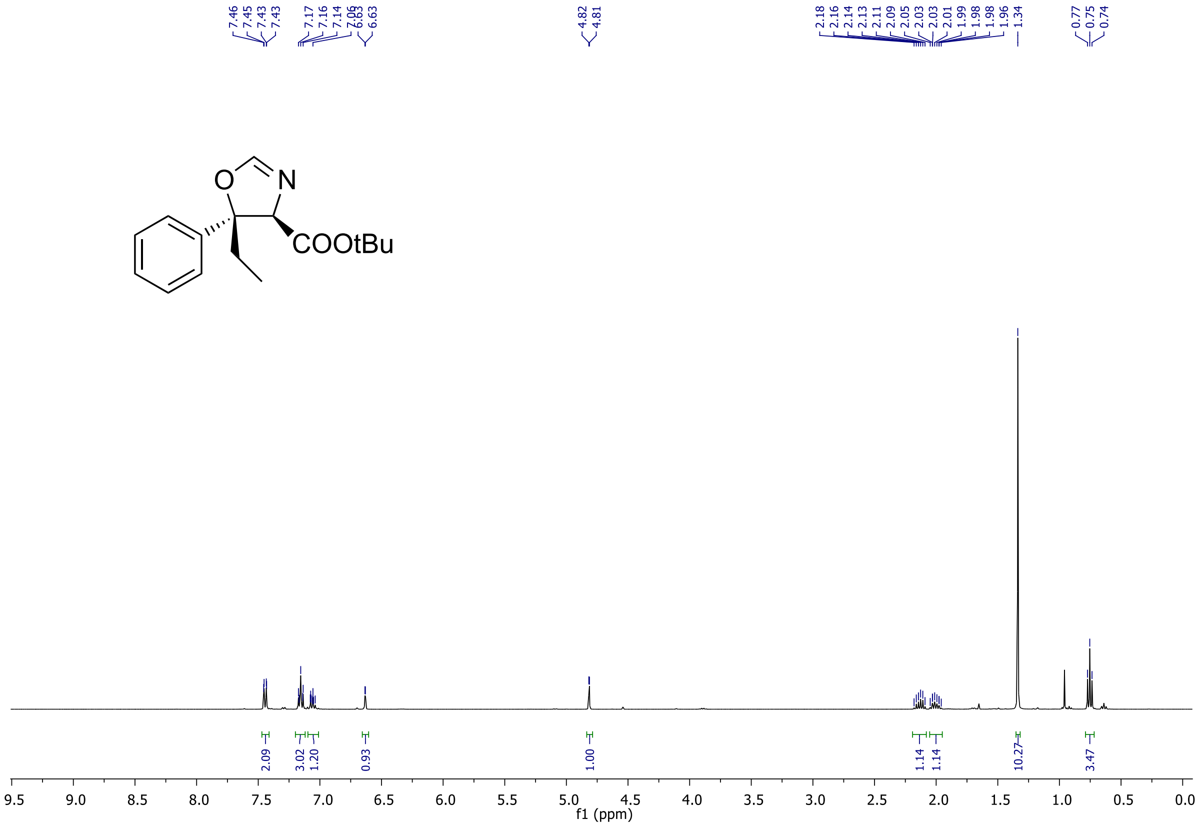

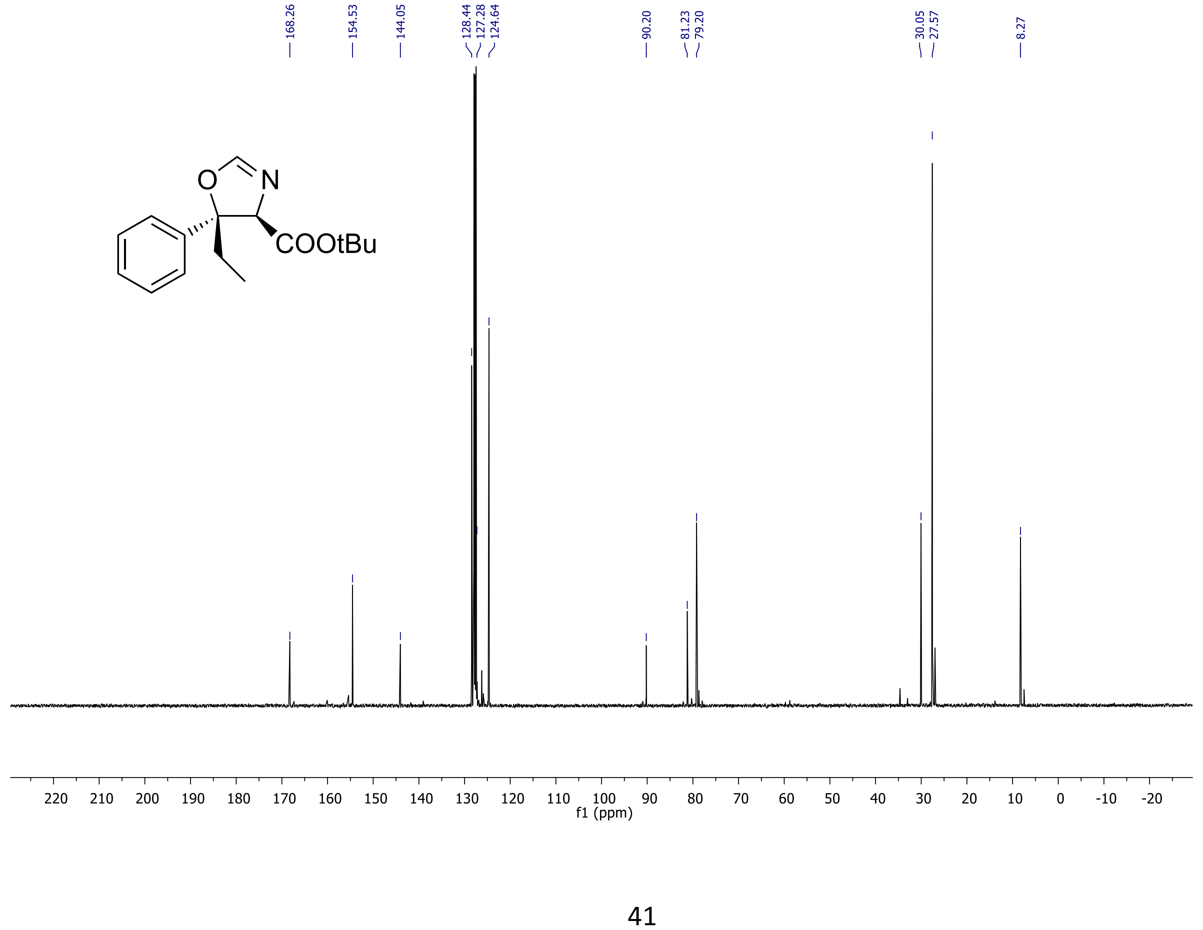

HPLC traces of racemic compound

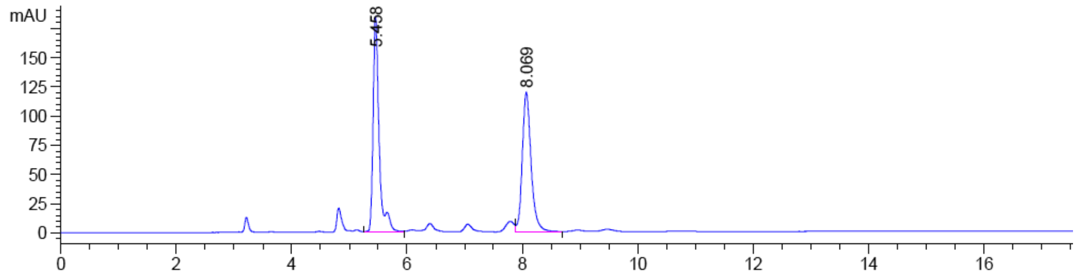

Peak #	RetTime [min]	Type	Width [min]	Area [mAU*s]	Height [mAU]	Area %
1	5.458	VV	0.1136	1347.77429	183.79715	51.2769
2	8.069	VB	0.1651	1280.64832	119.47324	48.7231

HPLC traces of **4k**

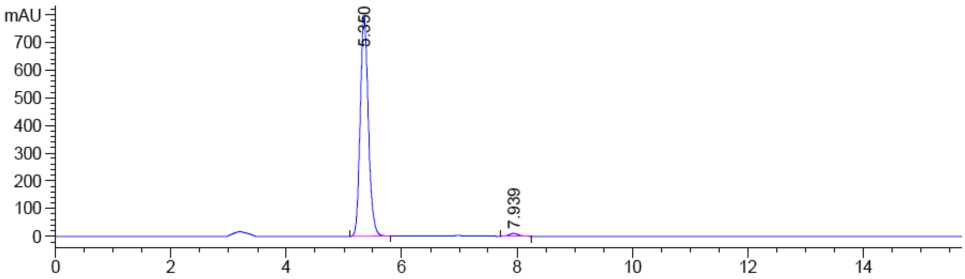

Peak #	RetTime [min]	Type	Width [min]	Area [mAU*s]	Height [mAU]	Area %
1	5.350	BB	0.1498	7733.61230	792.59088	98.6202
2	7.939	BB	0.1760	108.20496	9.63514	1.3798

HPLC traces of enantiomer of **4k**

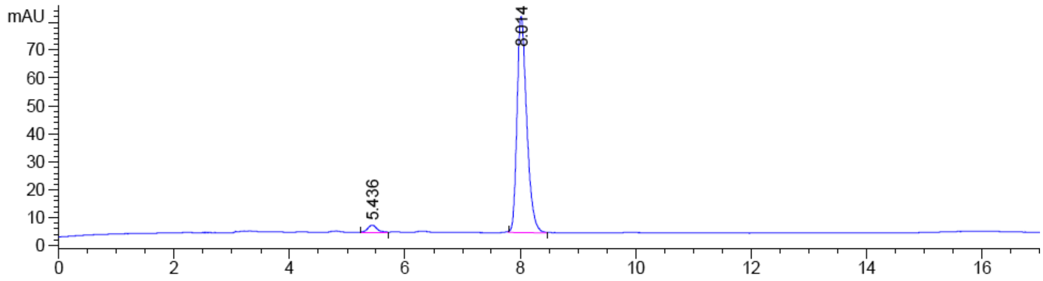

Peak #	RetTime [min]	Type	Width [min]	Area [mAU*s]	Height [mAU]	Area %
1	5.436	BB	0.1576	27.62828	2.56562	2.9586
2	8.014	BB	0.1799	906.18811	77.20967	97.0414

4.12 ^{1}H and ^{13}C-NMR spectra and HPLC traces of compound 4l

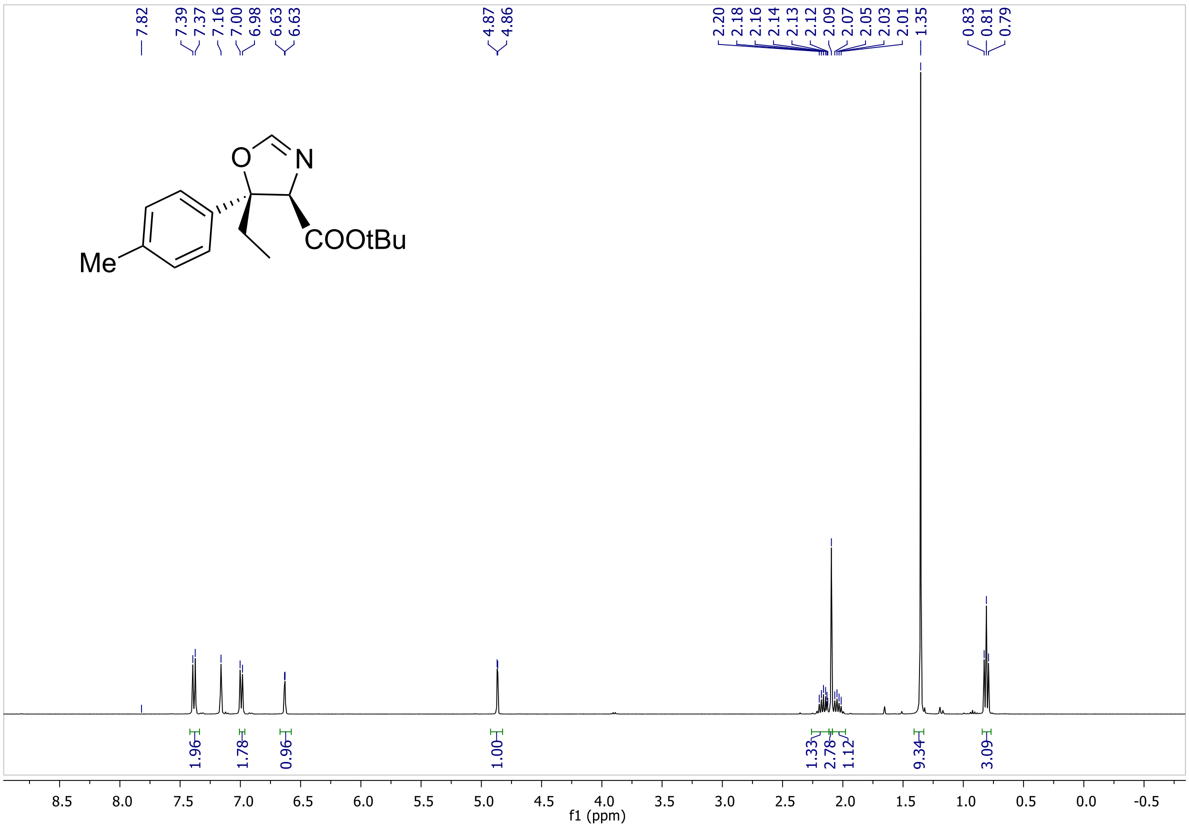

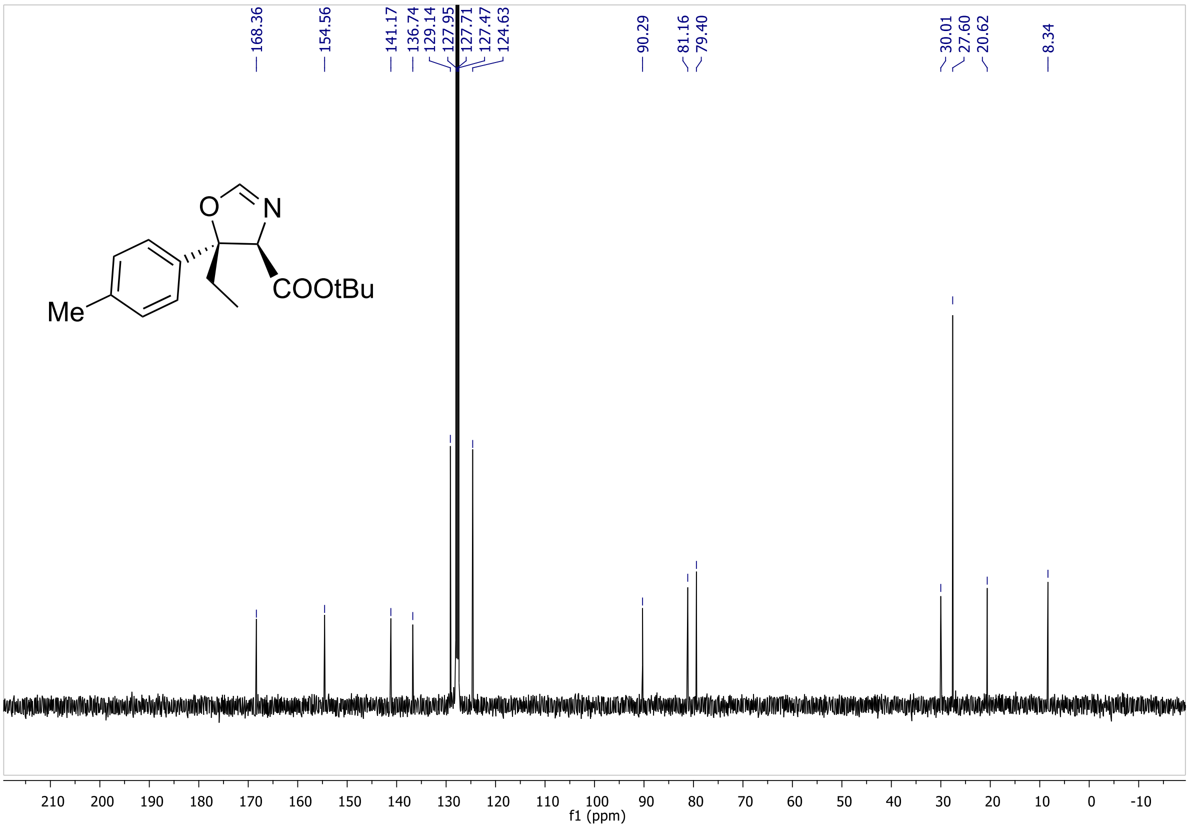

HPLC traces of **4l**

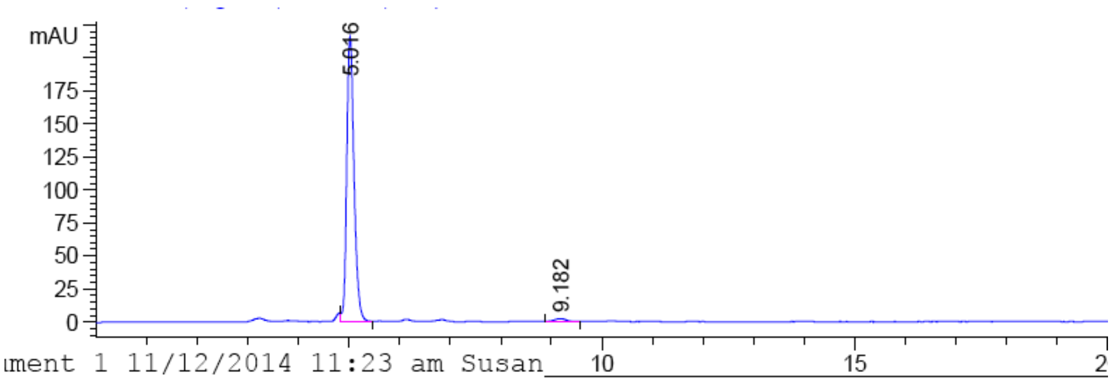

Peak #	RetTime [min]	Type	Width [min]	Area [mAU*s]	Height [mAU]	Area %
1	5.016	VB	0.1558	2176.78711	215.60901	98.3320
2	9.182	BB	0.2305	36.92415	2.33460	1.6680

HPLC traces of the enantiomer of **4l**

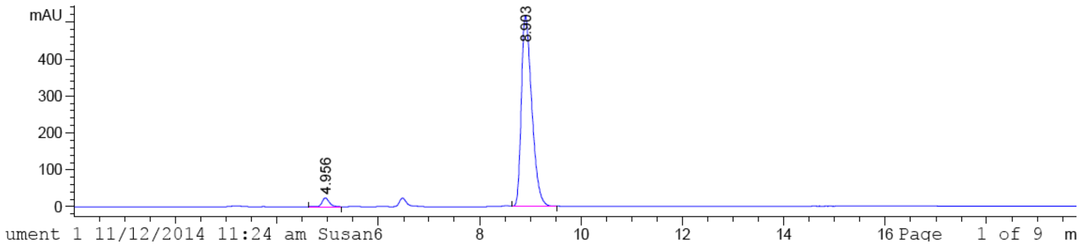

Peak #	RetTime [min]	Type	Width [min]	Area [mAU*s]	Height [mAU]	Area %
1	4.956	BB	0.1553	245.42758	23.99260	3.2051
2	8.903	VB	0.2191	7412.03564	517.47772	96.7949

HPCL of mixture of both enantiomers

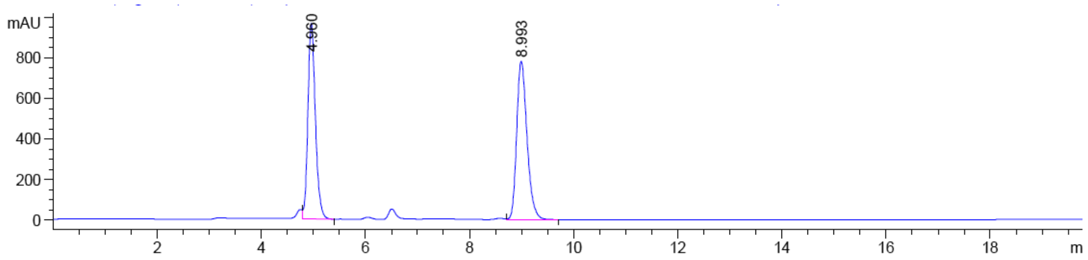

Peak #	RetTime [min]	Type	Width [min]	Area [mAU*s]	Height [mAU]	Area %
1	4.960	VV	0.1547	9606.81641	960.66986	47.2613
2	8.993	VB	0.2103	1.07202e4	780.04425	52.7387

4.13 ^{1}H and ^{13}C-NMR spectra and HPLC traces of compound 4m

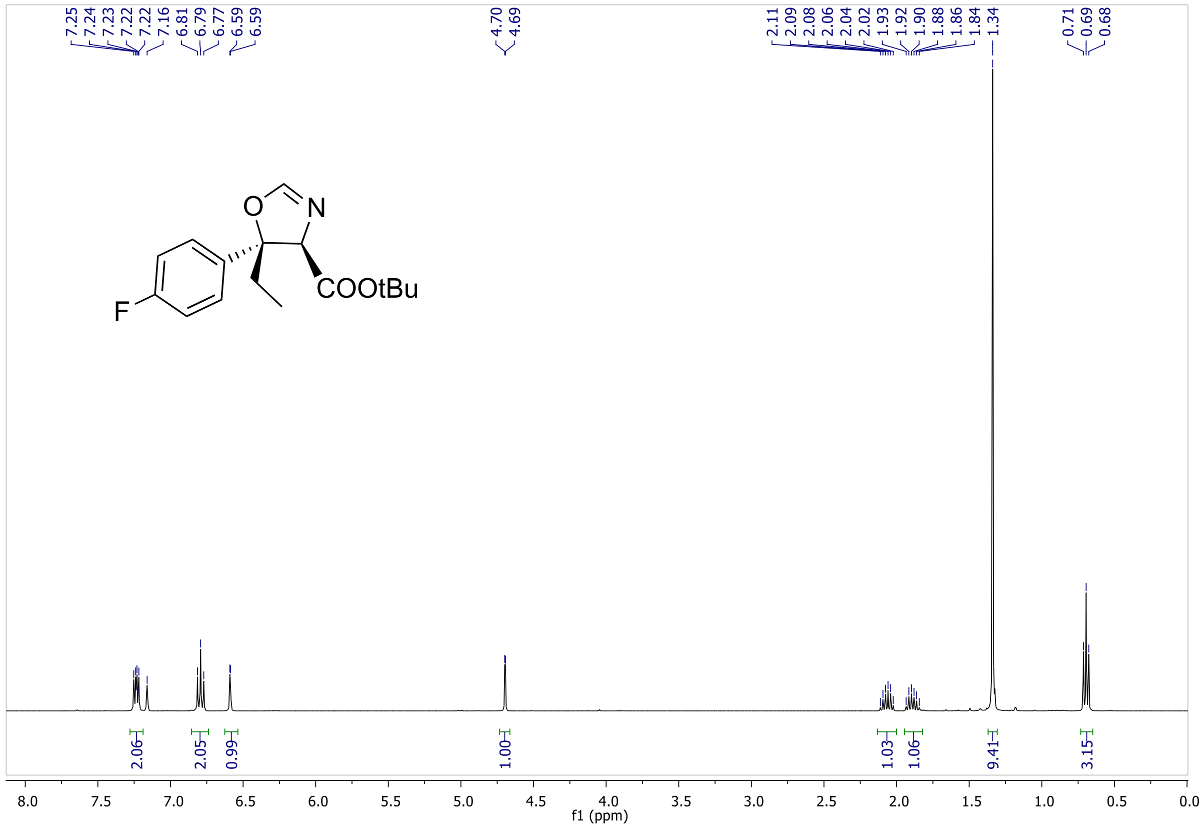

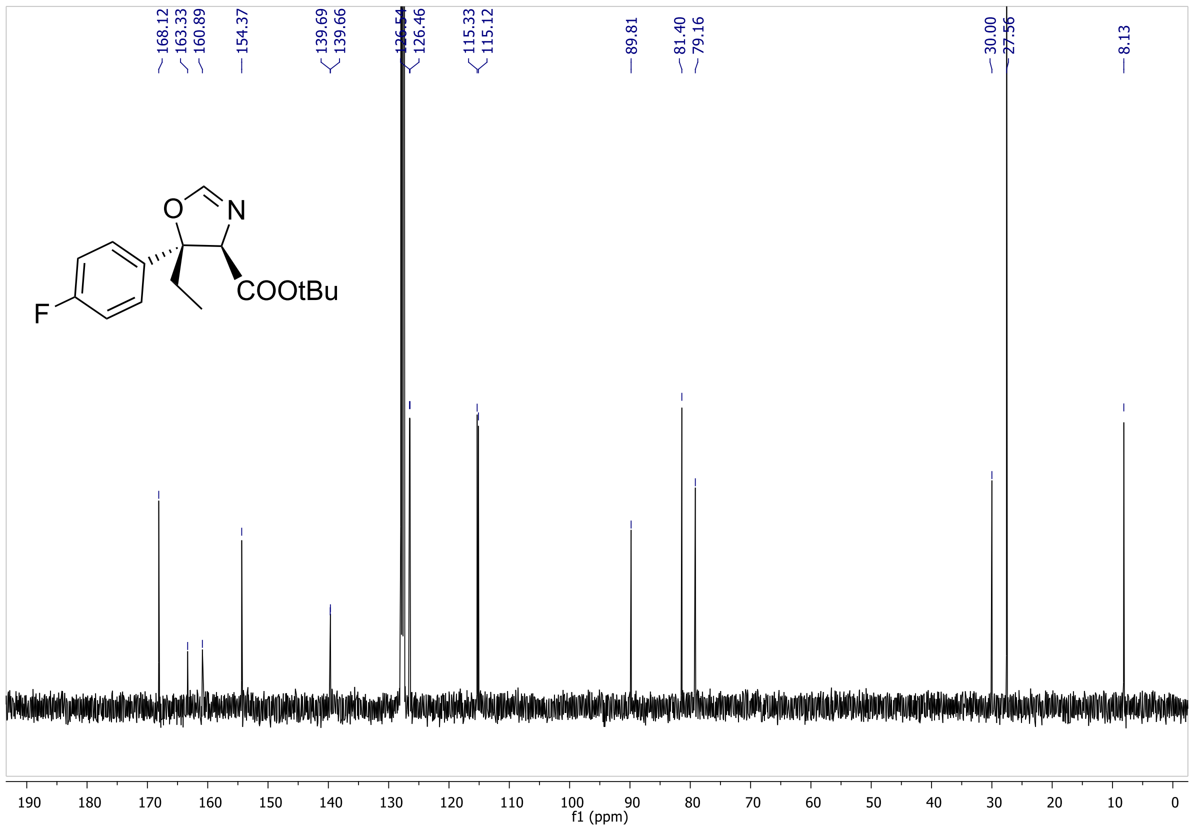

HPLC traces of racemic compound

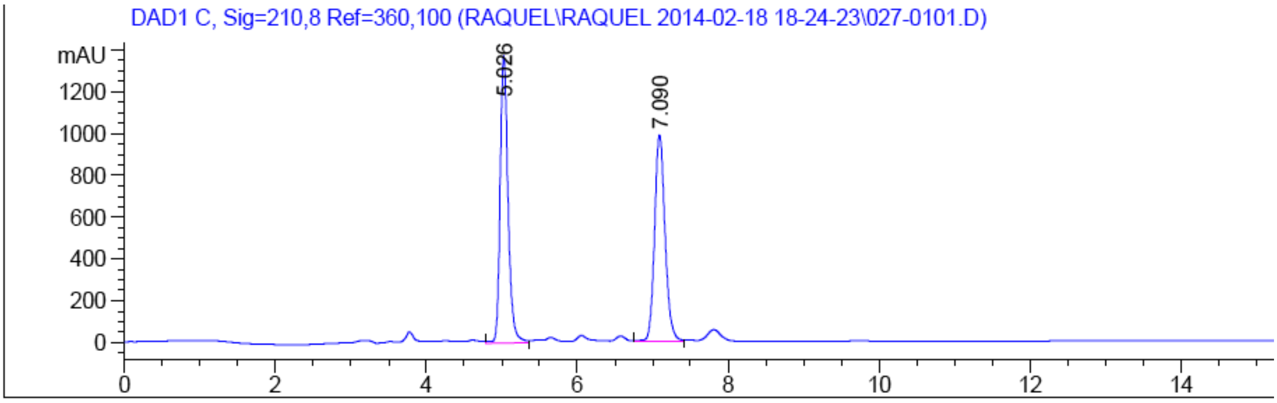

Peak #	RetTime [min]	Type	Width [min]	Area [mAU*s]	Height [mAU]	Area %
1	5.026	VV	0.1113	1.00346e4	1372.18091	50.8881
2	7.090	VV	0.1500	9684.30469	991.10419	49.1119

Totals : 1.97189e4 2363.28510

HPLC traces of **4m**

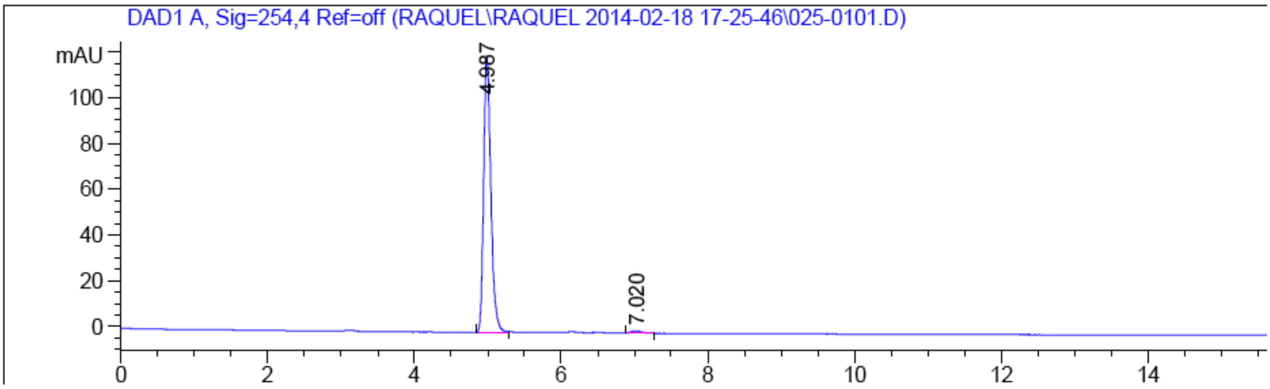

Peak #	RetTime [min]	Type	Width [min]	Area [mAU*s]	Height [mAU]	Area %
1	4.987	BB	0.1043	818.36011	120.46494	98.8065
2	7.020	BB	0.1407	9.88481	1.03221	1.1935

4.14 ^{1}H and ^{13}C-NMR spectra and HPLC traces of compound 4n

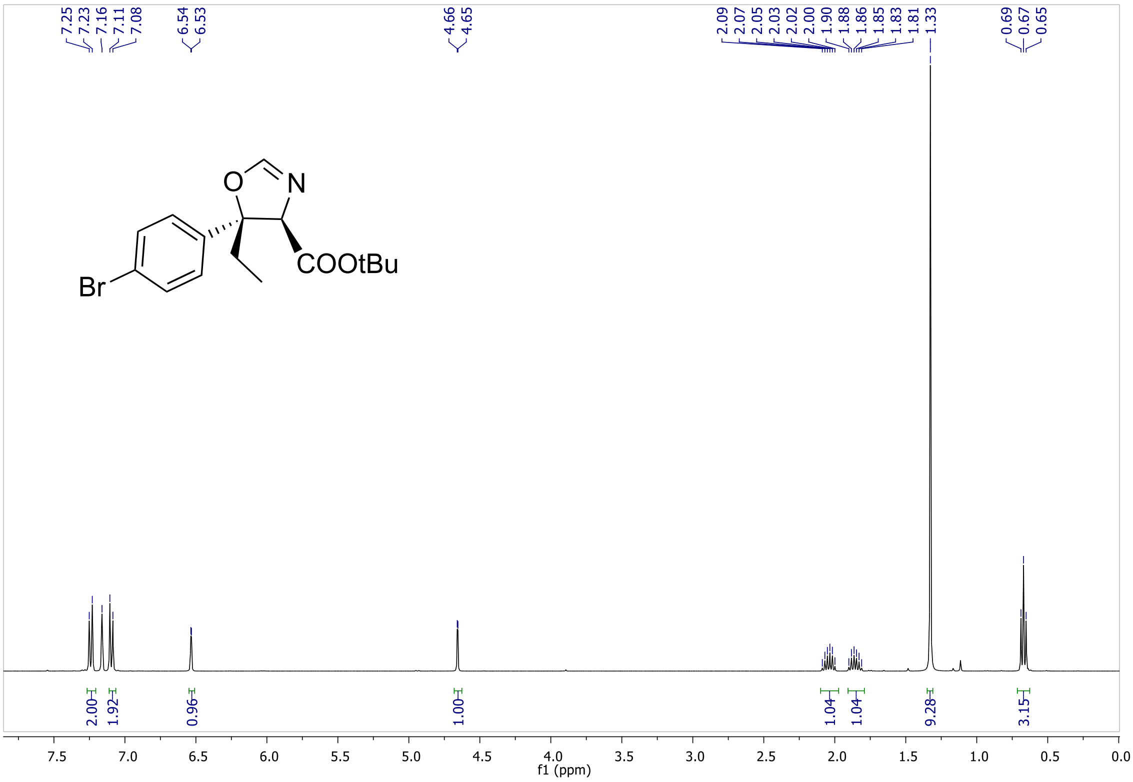

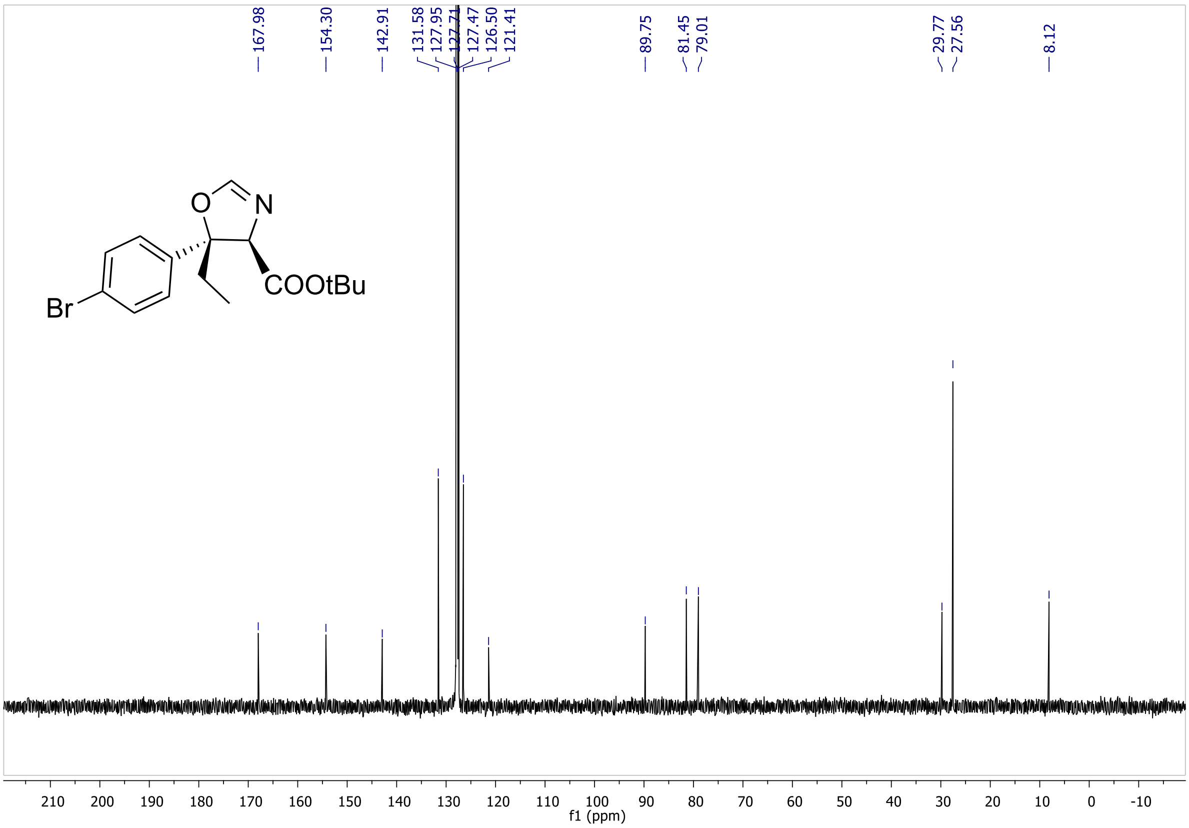

HPLC traces of racemic compound

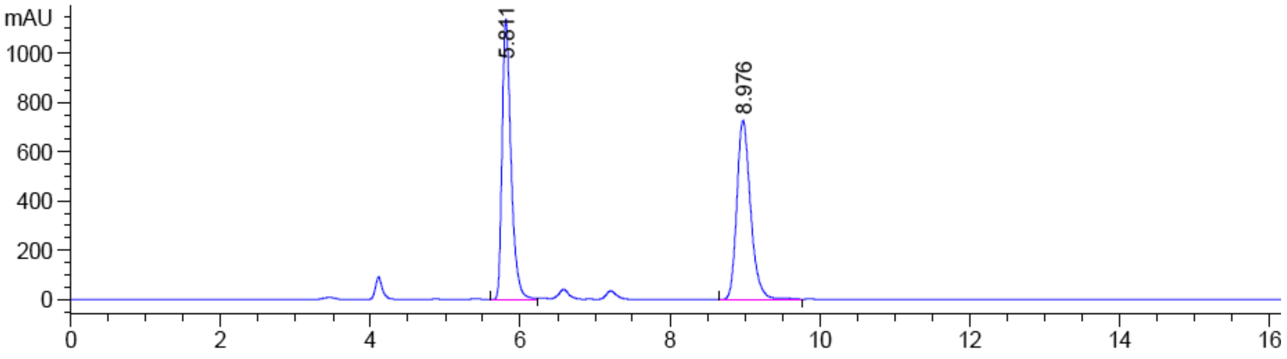

Peak #	RetTime [min]	Type	Width [min]	Area [mAU*s]	Height [mAU]	Area %
1	5.811	VV	0.1285	9434.42578	1140.90820	50.2442
2	8.976	VV	0.1979	9342.73047	727.04376	49.7558

HPLC traces of **4n**

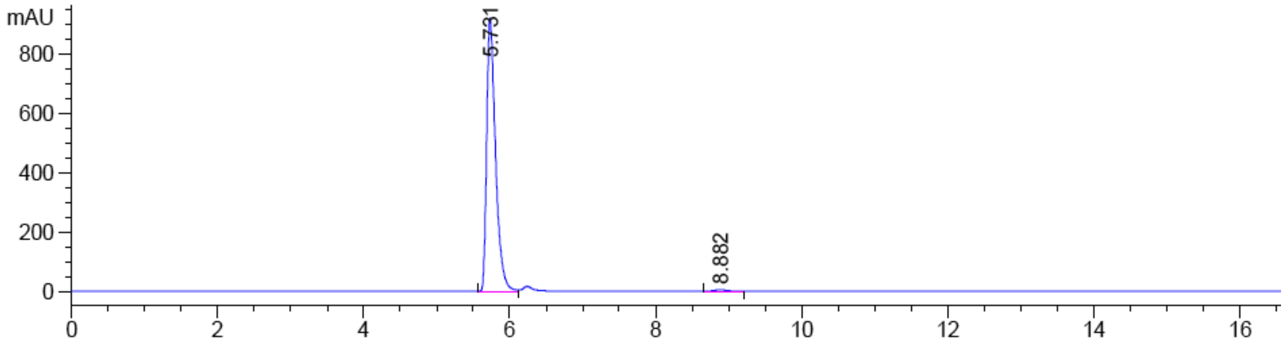

Peak #	RetTime [min]	Type	Width [min]	Area [mAU*s]	Height [mAU]	Area %
1	5.731	BV	0.1274	7774.39209	921.44147	99.3077
2	8.882	BB	0.1735	54.19931	4.53205	0.6923

4.15 ^{1}H and ^{13}C-NMR spectra and HPLC traces of compound 4o

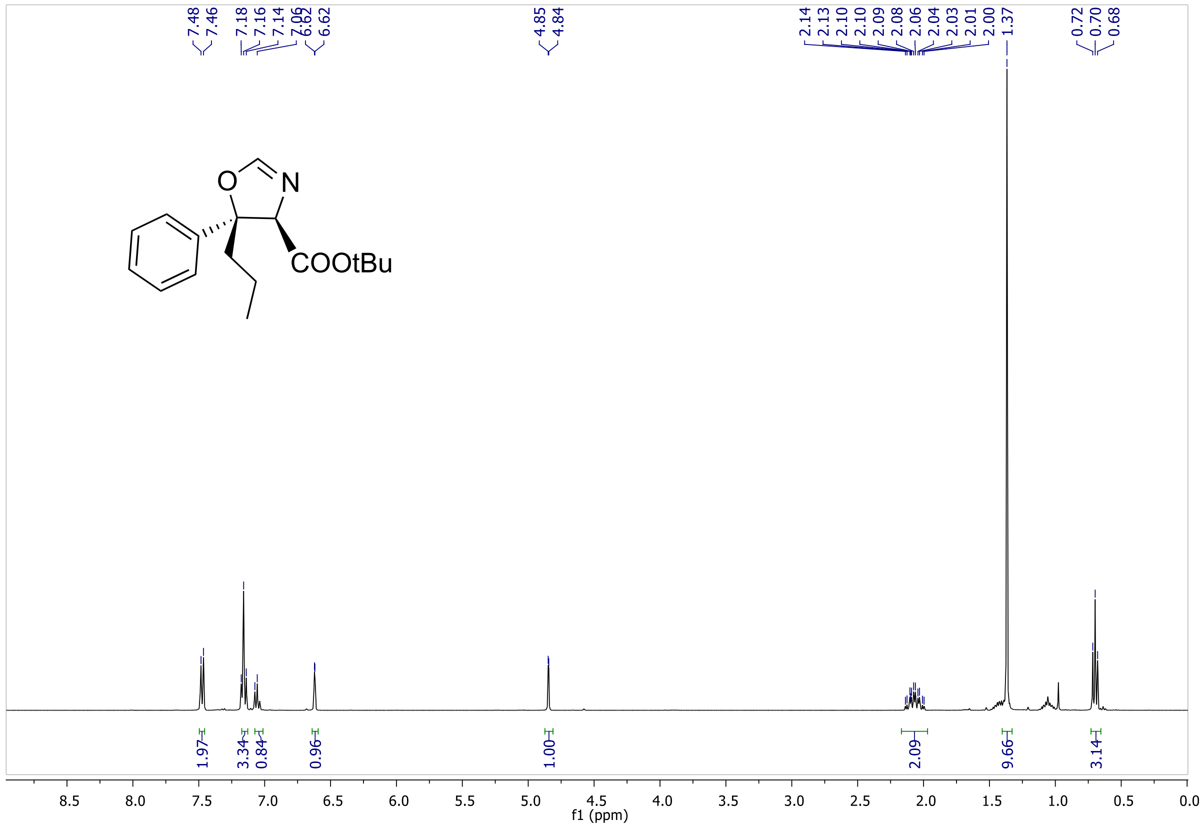

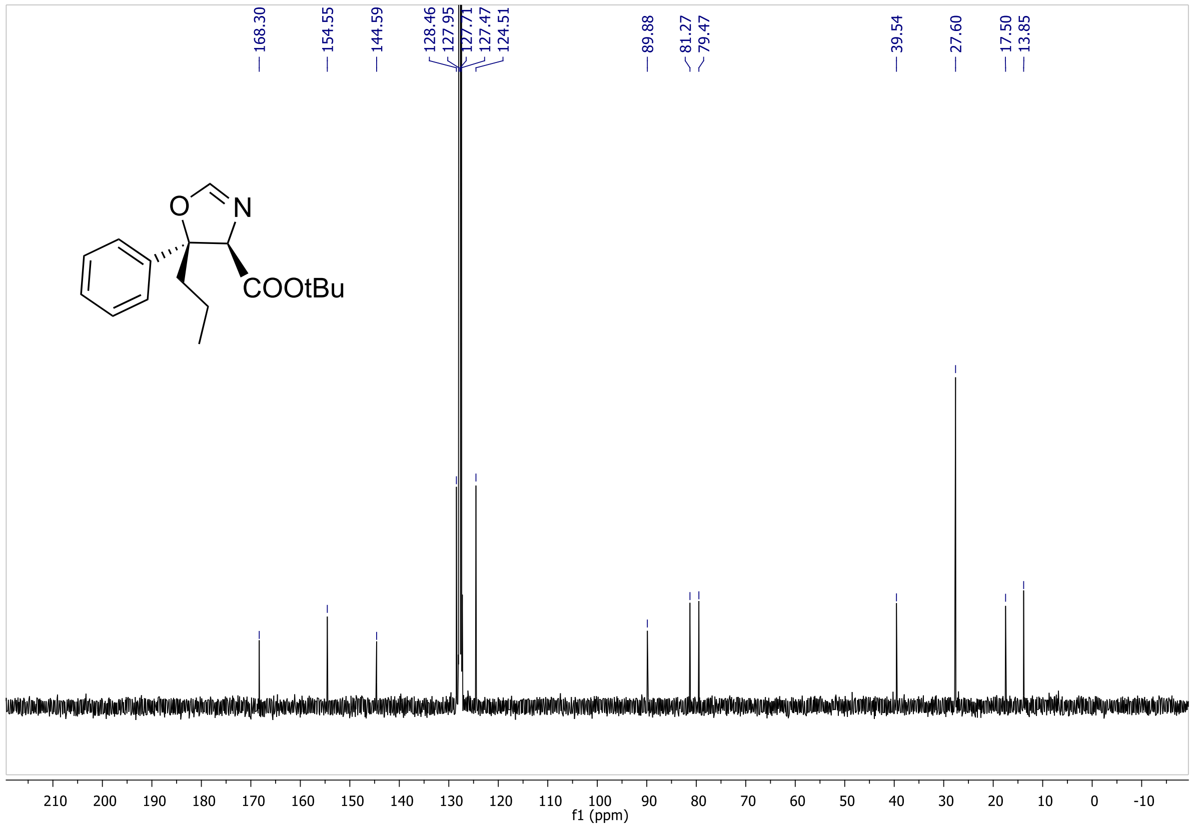

HPLC traces of racemic compound

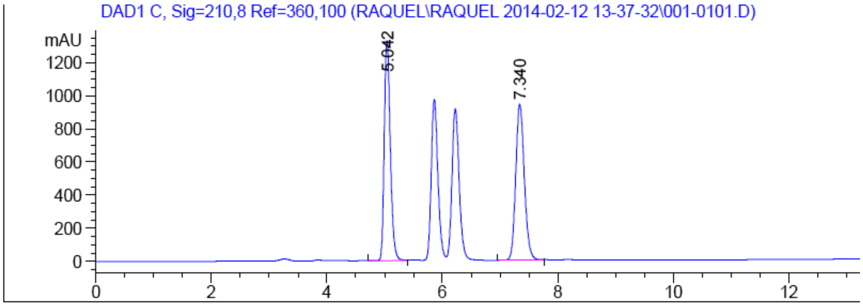

Peak #	RetTime [min]	Type	Width [min]	Area [mAU*s]	Height [mAU]	Area %
1	5.042	VV	0.1128	9642.98633	1326.78284	49.5509
2	7.340	BB	0.1618	9817.77734	940.26056	50.4491

HPLC traces of **4o**

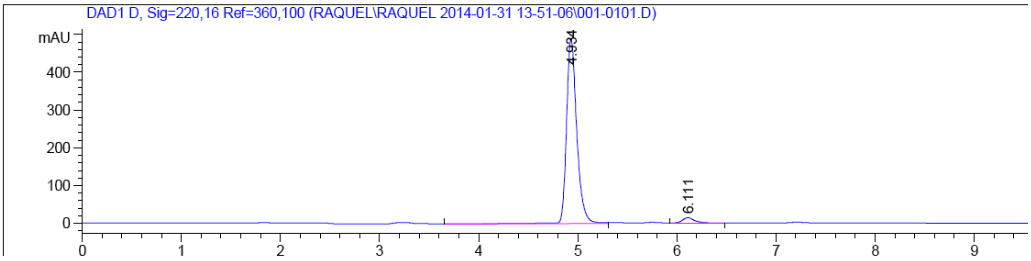

Peak #	RetTime [min]	Type	Width [min]	Area [mAU*s]	Height [mAU]	Area %
1	4.931	BB	0.1074	174.25977	24.98550	100.0000

HPLC traces of the enantiomer of **4o**

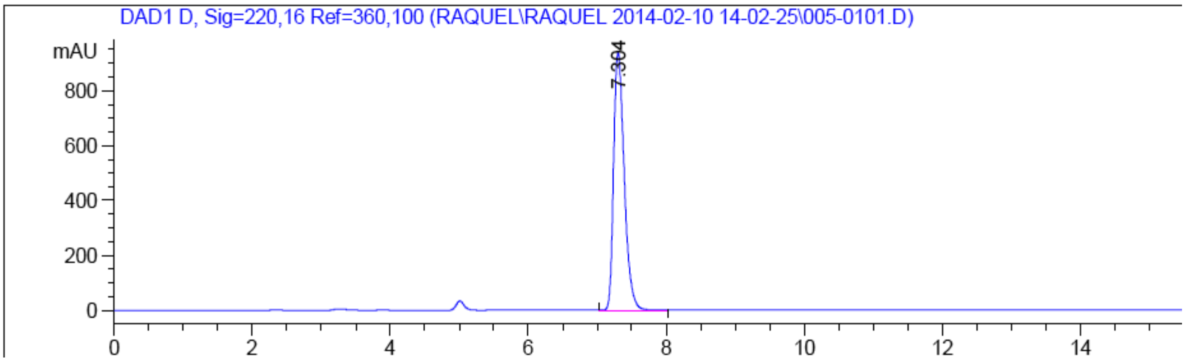

Peak #	RetTime [min]	Type	Width [min]	Area [mAU*s]	Height [mAU]	Area %
1	7.304	BV	0.1646	1.00548e4	941.15955	100.0000

HPLC traces of mixture of both enantiomers

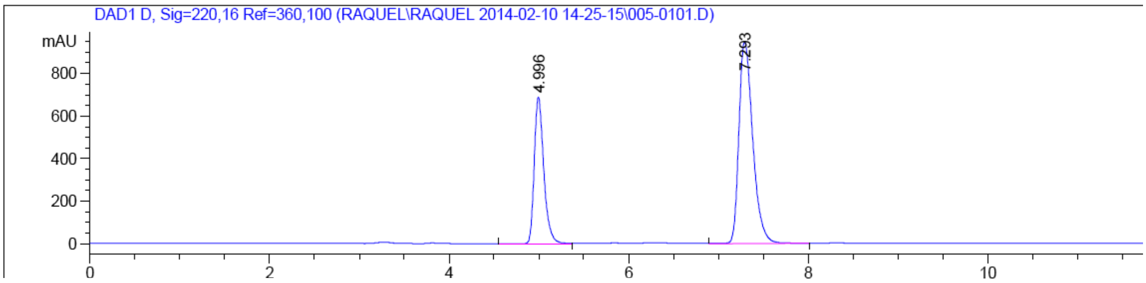

Peak #	RetTime [min]	Type	Width [min]	Area [mAU*s]	Height [mAU]	Area %
1	4.996	BV	0.1184	5099.17529	688.59570	33.3876
2	7.293	VV	0.1661	1.01735e4	941.35156	66.6124

4.16 ^{1}H and ^{13}C-NMR spectra and HPLC traces of compound 4p

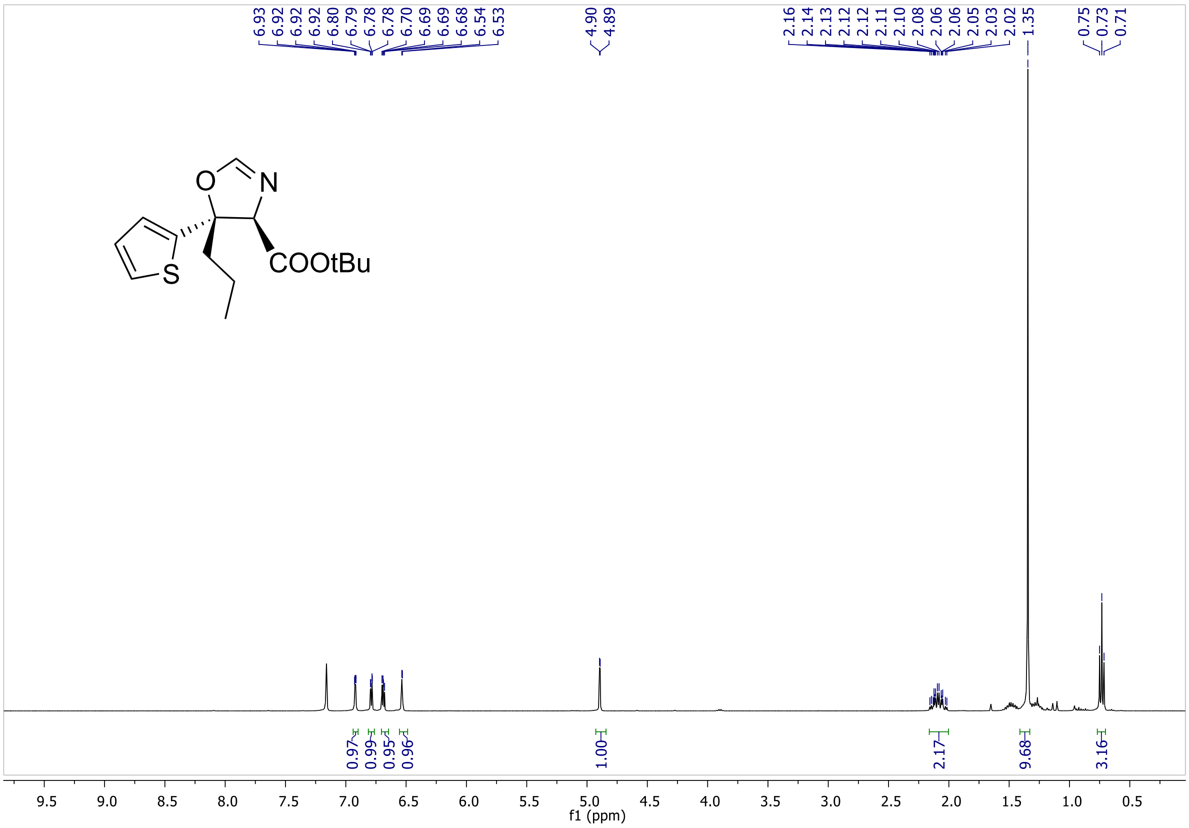

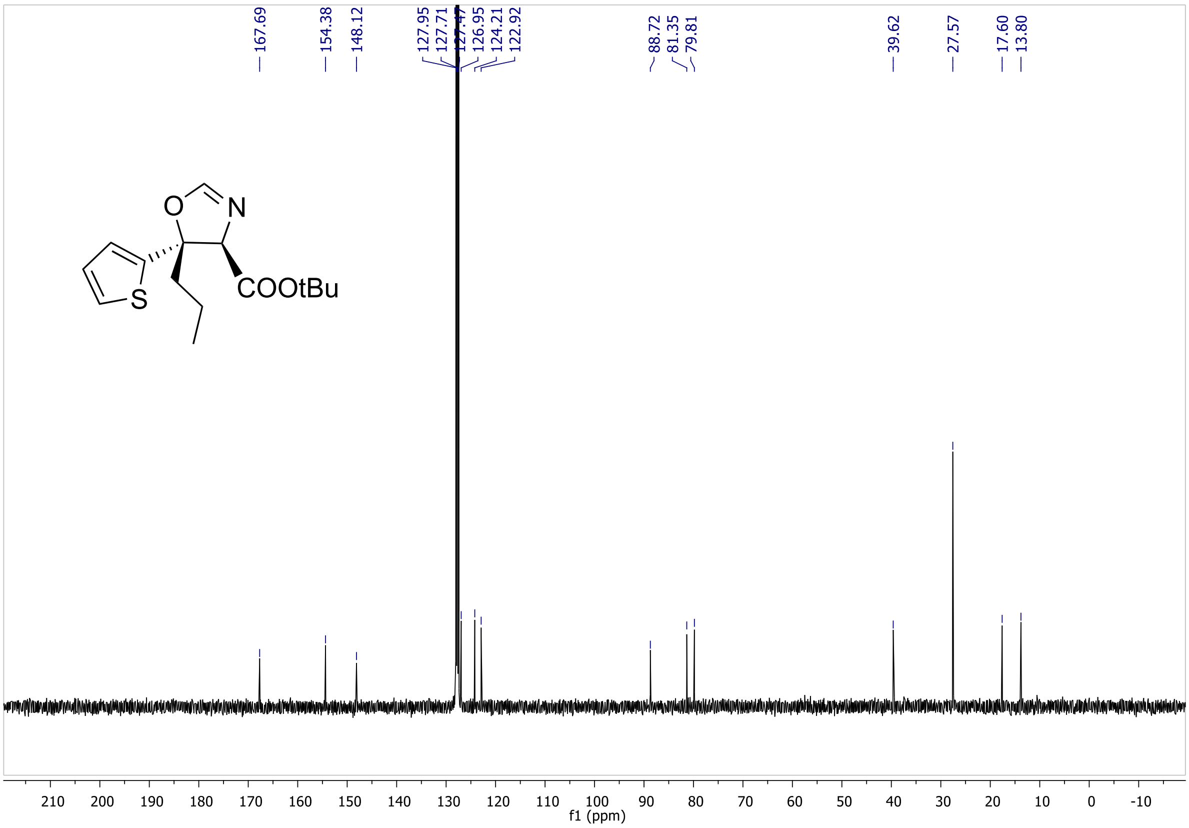

HPLC traces of racemic compound

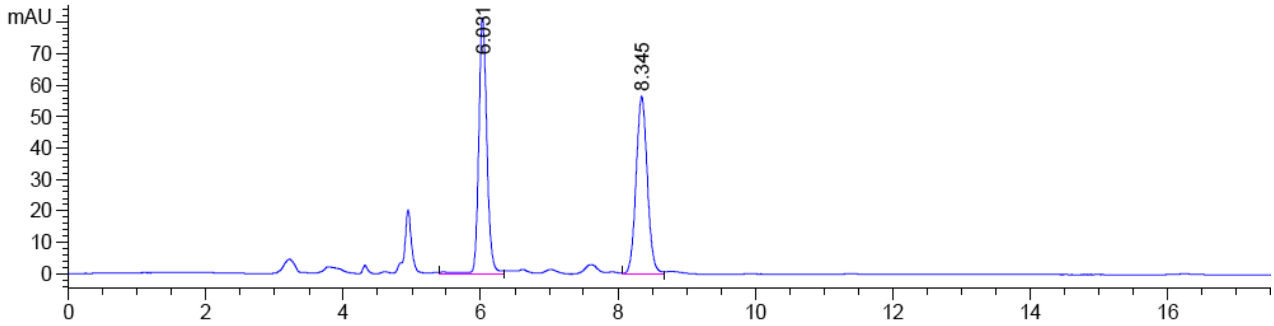

Peak #	RetTime [min]	Type	Width [min]	Area [mAU*s]	Height [mAU]	Area %
1	6.031	BB	0.1246	387.05649	47.71211	51.7795
2	8.345	VB	0.1757	360.45273	31.92039	48.2205

HPLC traces of **4p**

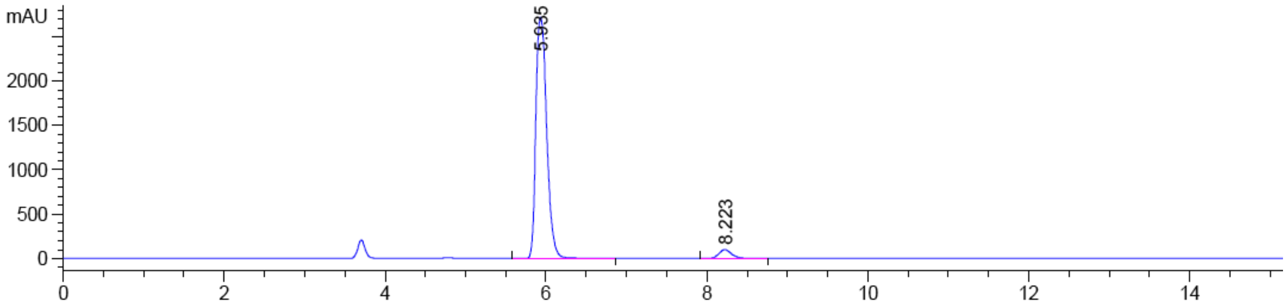

Peak #	RetTime [min]	Type	Width [min]	Area [mAU*s]	Height [mAU]	Area %
1	5.932	BB	0.1267	1.60698e4	1938.75146	96.3445
2	8.222	BB	0.1673	609.71246	55.86193	3.6555

4.17 ^{1}H and ^{13}C-NMR spectra and HPLC traces of compound 4q

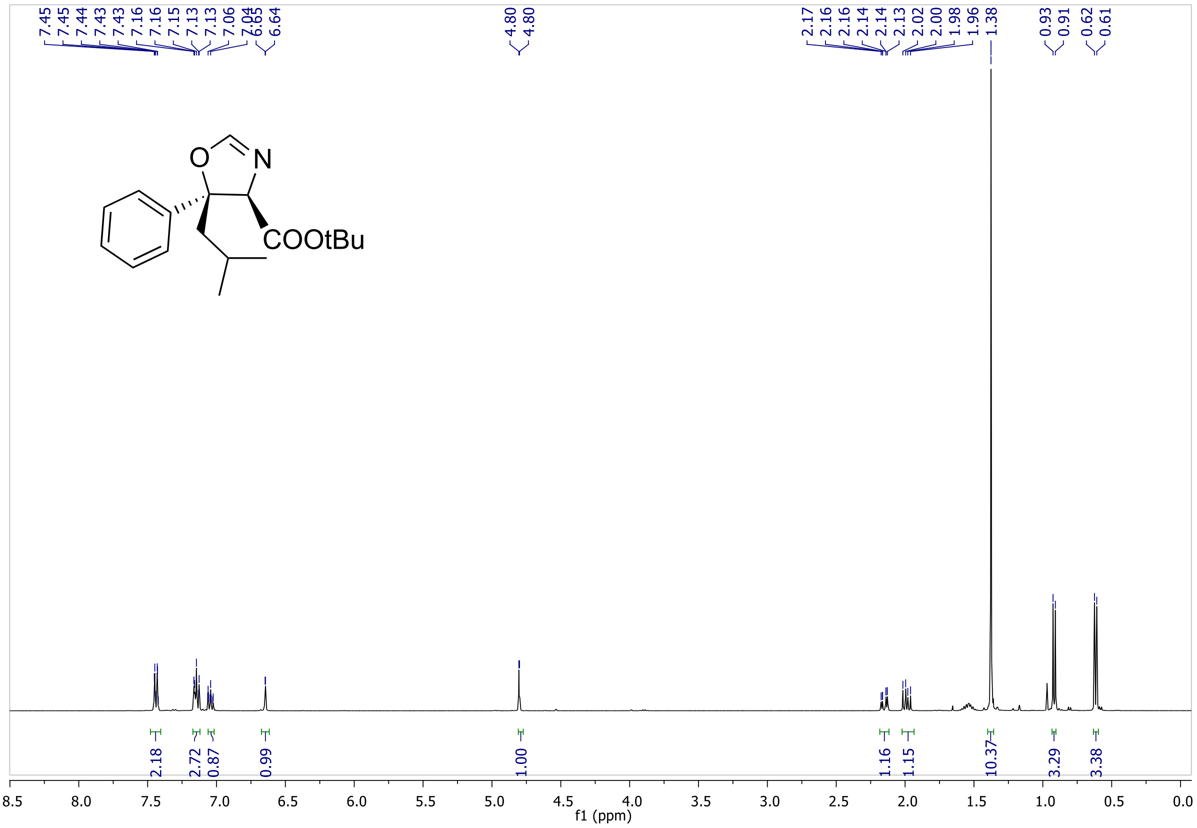

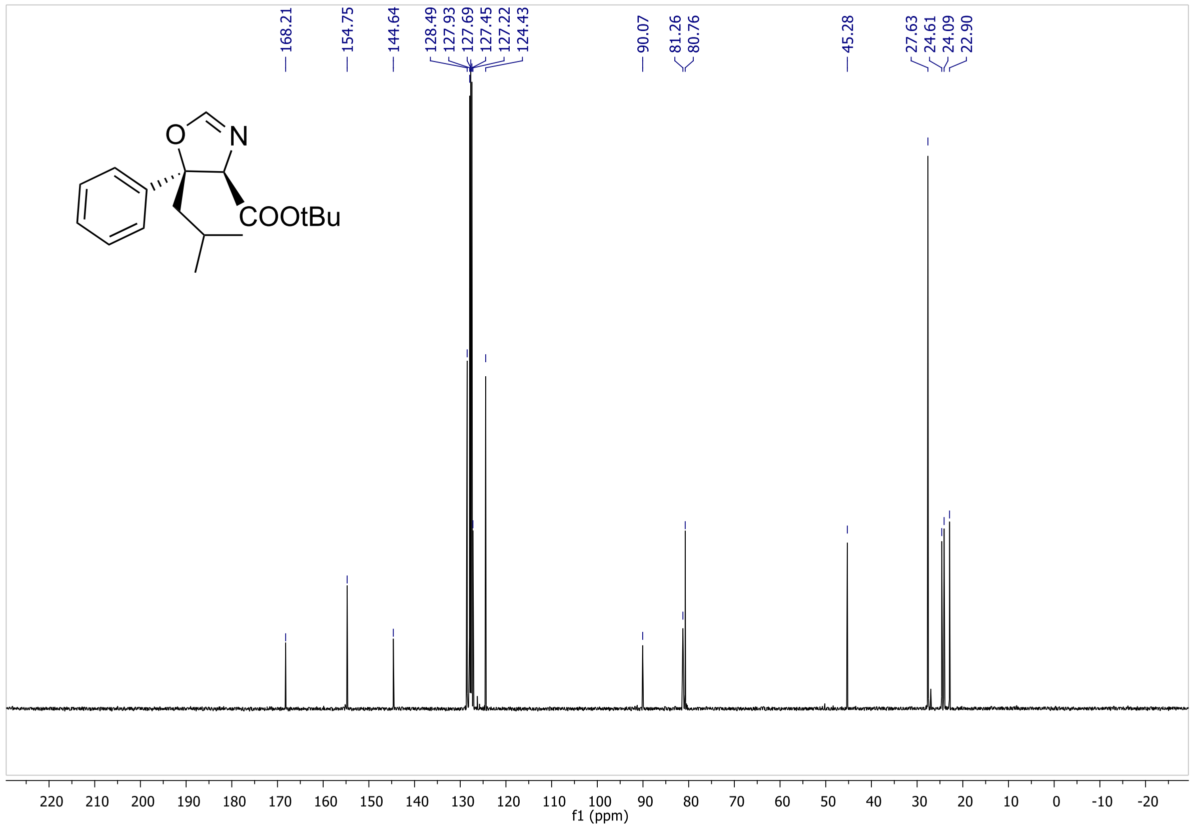

HPLC traces of **4q**

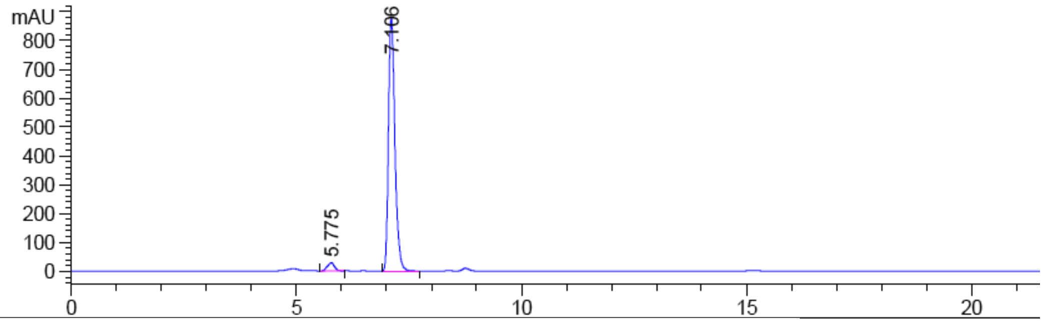

Peak #	RetTime [min]	Type	Width [min]	Area [mAU*s]	Height [mAU]	Area %
1	5.775	VB	0.1645	308.13486	28.41267	3.4005
2	7.106	BB	0.1534	8753.33301	877.46674	96.5995

HPLC traces of the enantiomer of **4q**

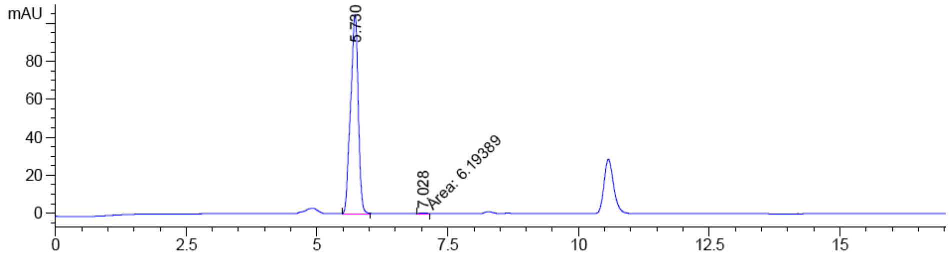

Peak #	RetTime [min]	Type	Width [min]	Area [mAU*s]	Height [mAU]	Area %
1	5.730	BB	0.1498	1067.13123	104.80763	99.4229
2	7.028	MM	0.1750	6.19389	5.89914e-1	0.5771

HPLC traces of mixture of both enantiomers

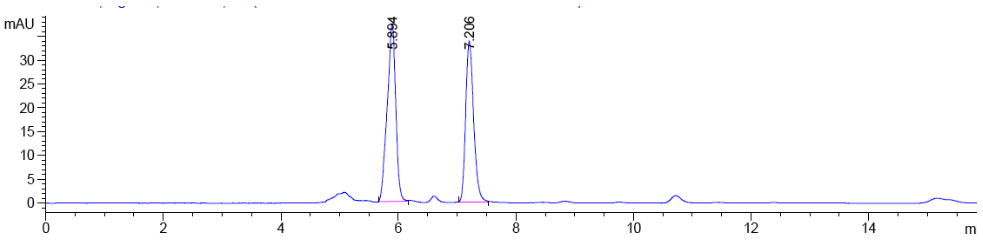

Peak #	RetTime [min]	Type	Width [min]	Area [mAU*s]	Height [mAU]	Area %
1	5.894	BB	0.1482	376.91049	37.19103	54.1739
2	7.206	BB	0.1457	318.83081	33.92261	45.8261

4.18 ^{1}H and ^{13}C-NMR spectra and HPLC traces of compound 4r

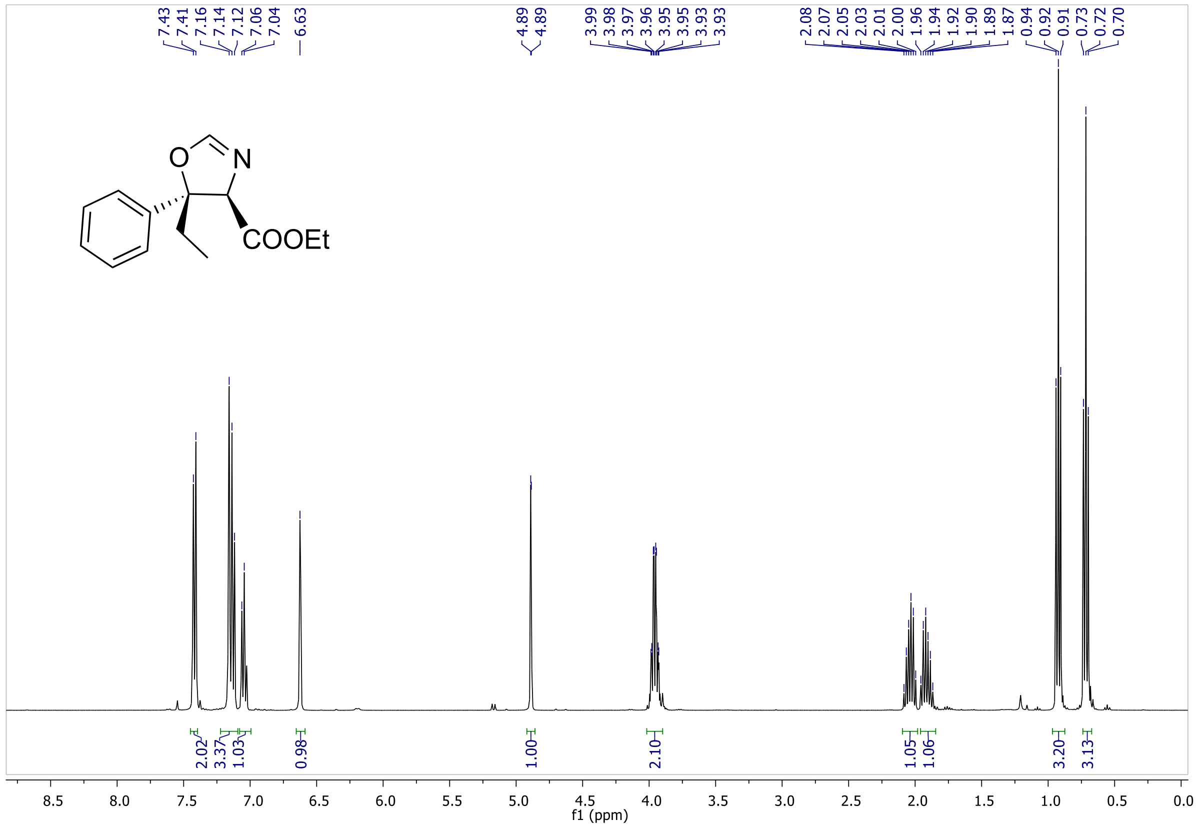

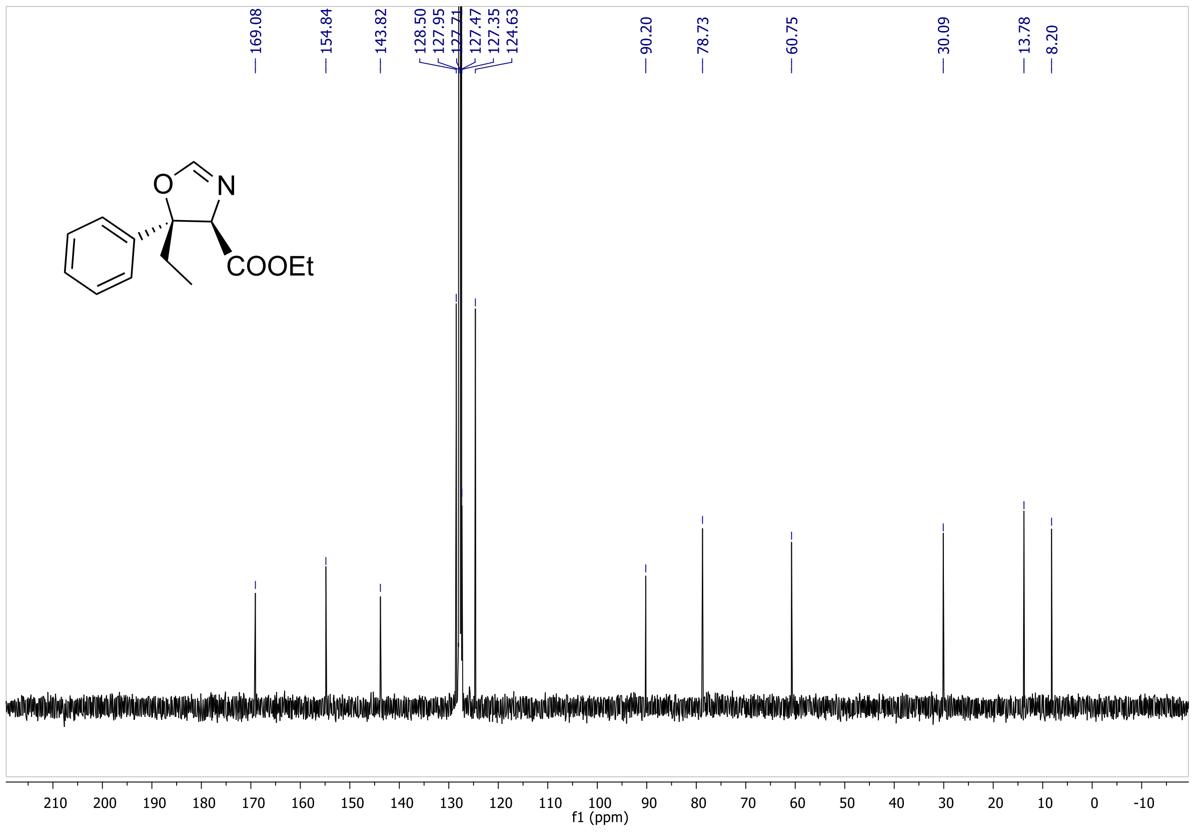

HPLC traces of **4r**

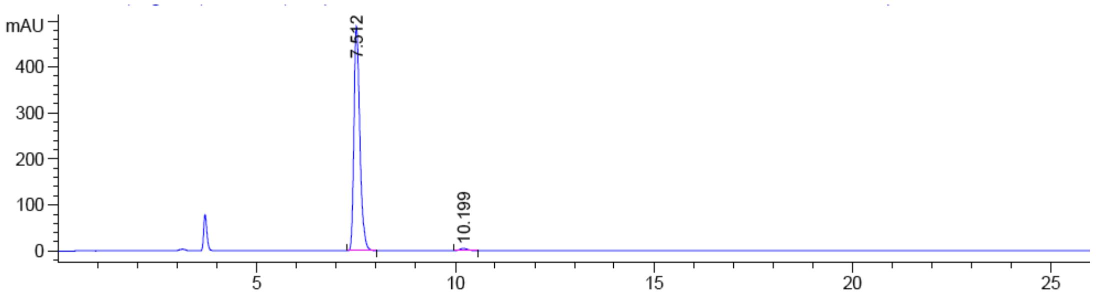

Peak #	RetTime [min]	Type	Width [min]	Area [mAU*s]	Height [mAU]	Area %
1	7.512	VB	0.1592	5067.86621	487.94693	98.5910
2	10.199	BB	0.2217	72.42654	4.97817	1.4090

HPLC traces of the enantiomer of **4r**

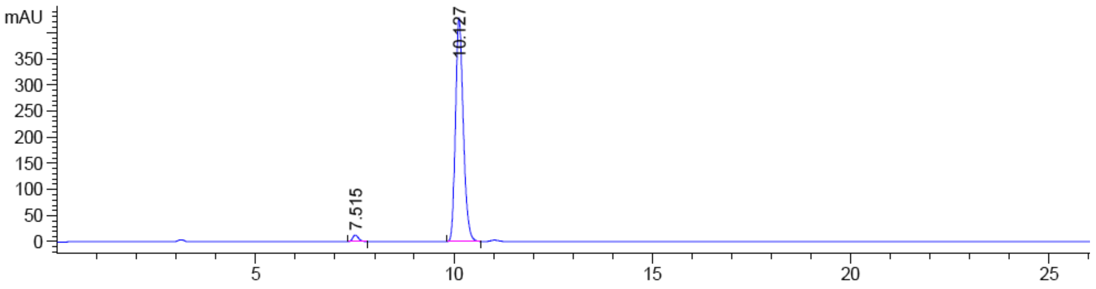

Peak #	RetTime [min]	Type	Width [min]	Area [mAU*s]	Height [mAU]	Area %
1	7.515	BB	0.1581	131.48410	12.76887	2.2005
2	10.127	BB	0.2092	5843.82031	428.22617	97.7995

HPLC traces of mixture of both enantiomers

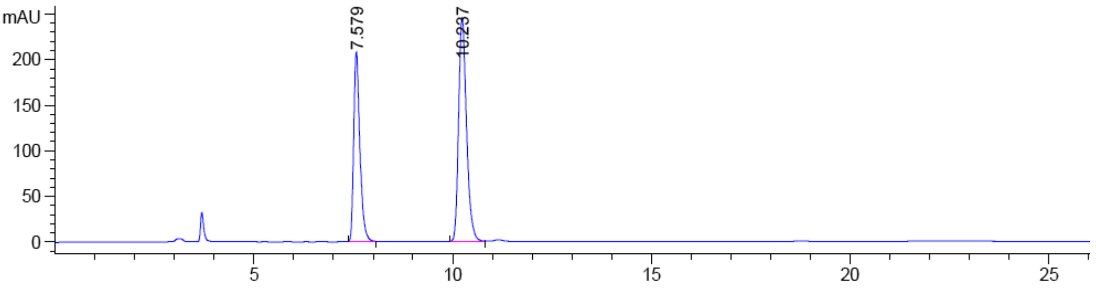

Peak #	RetTime [min]	Type	Width [min]	Area [mAU*s]	Height [mAU]	Area %
1	7.579	BB	0.1608	2225.79517	207.99393	39.3794
2	10.237	BB	0.2129	3426.38330	245.46373	60.6206

4.19 ^{1}H and ^{13}C-NMR spectra and HPLC traces of compound 4s

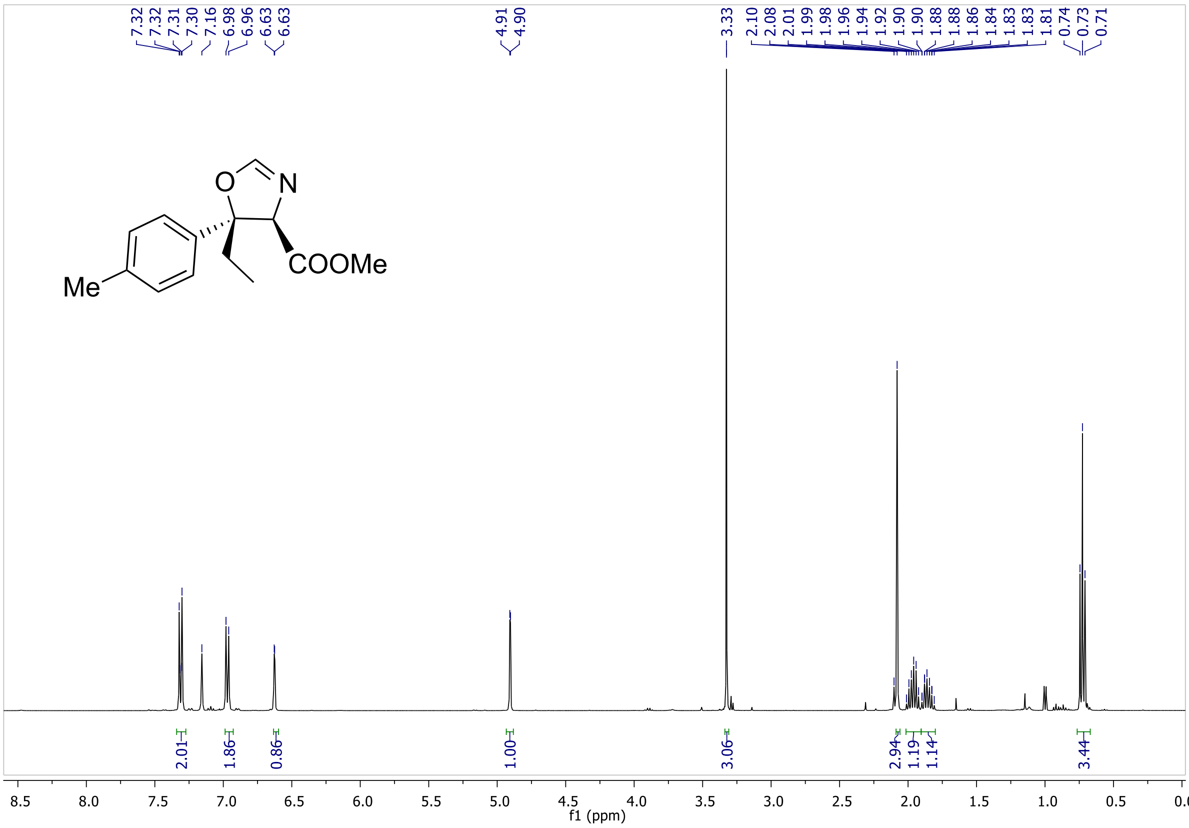

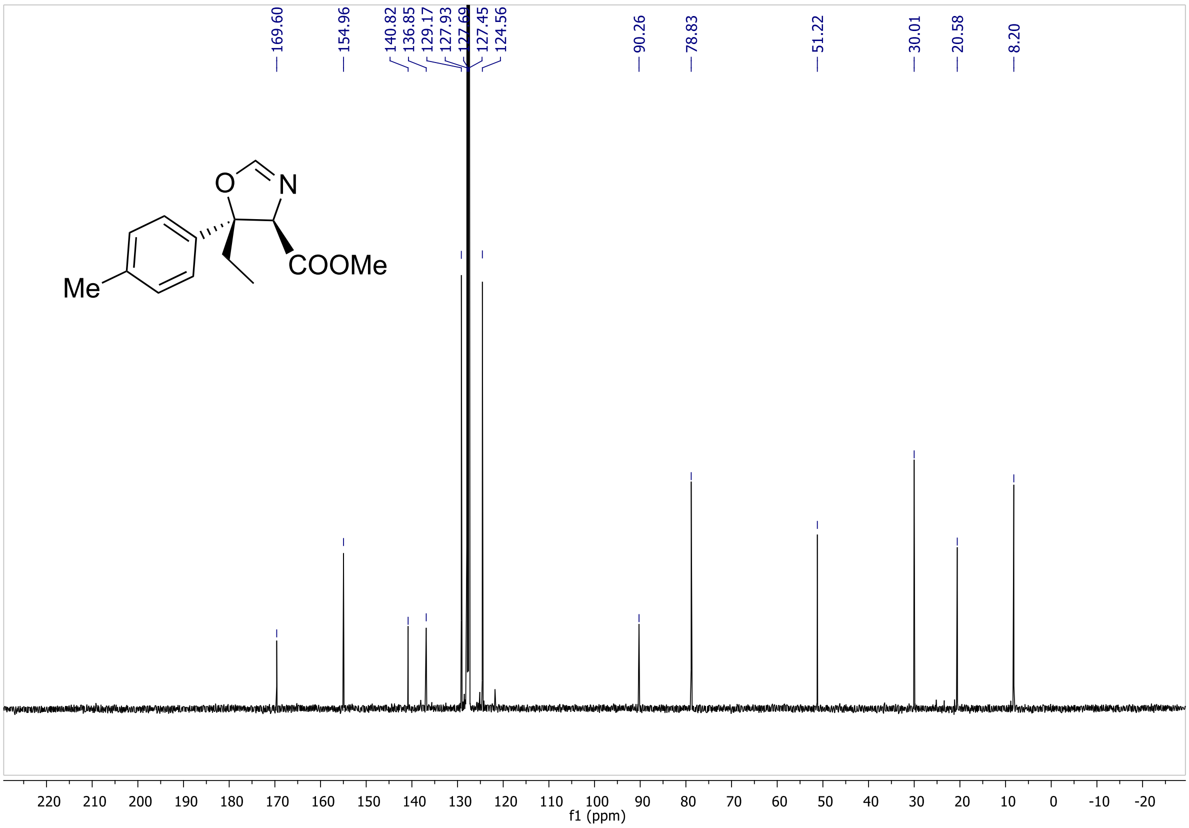

HPLC traces of racemic compound

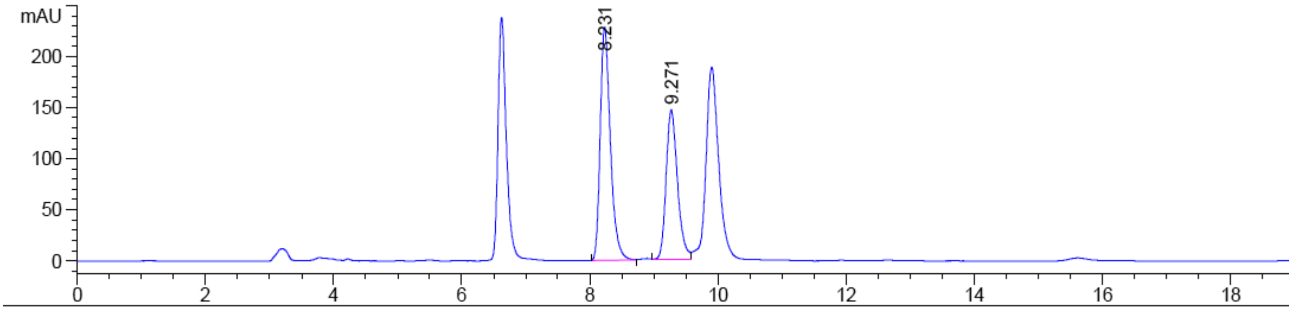

Peak #	RetTime [min]	Type	Width [min]	Area [mAU*s]	Height [mAU]	Area %
1	8.233	VV	0.1741	1089.13306	97.63374	59.1131
2	9.272	VV	0.1916	753.32410	59.55931	40.8869

HPLC traces of **4s**

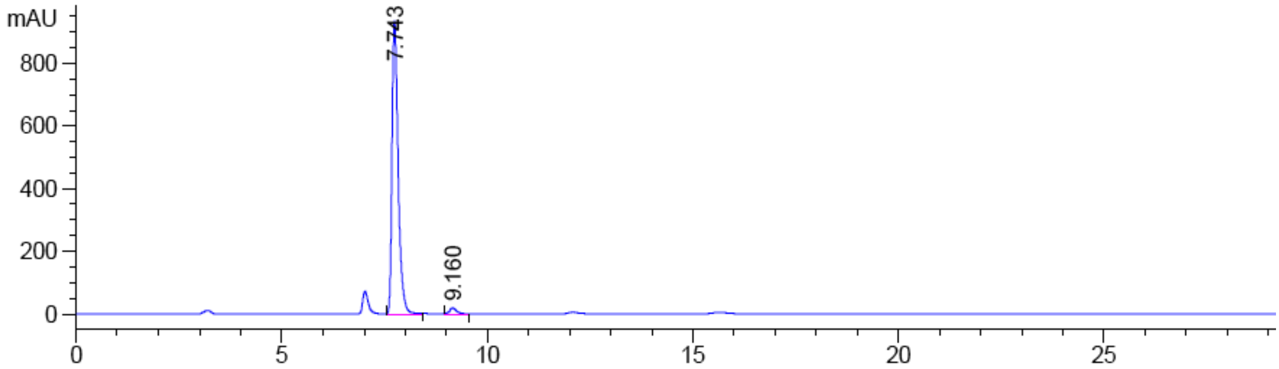

Peak #	RetTime [min]	Type	Width [min]	Area [mAU*s]	Height [mAU]	Area %
1	7.743	BB	0.1591	9795.77246	935.92224	97.8341
2	9.160	BB	0.1817	216.85867	18.11472	2.1659

4.20 ^{1}H and ^{13}C-NMR spectra and HPLC traces of compound 4t

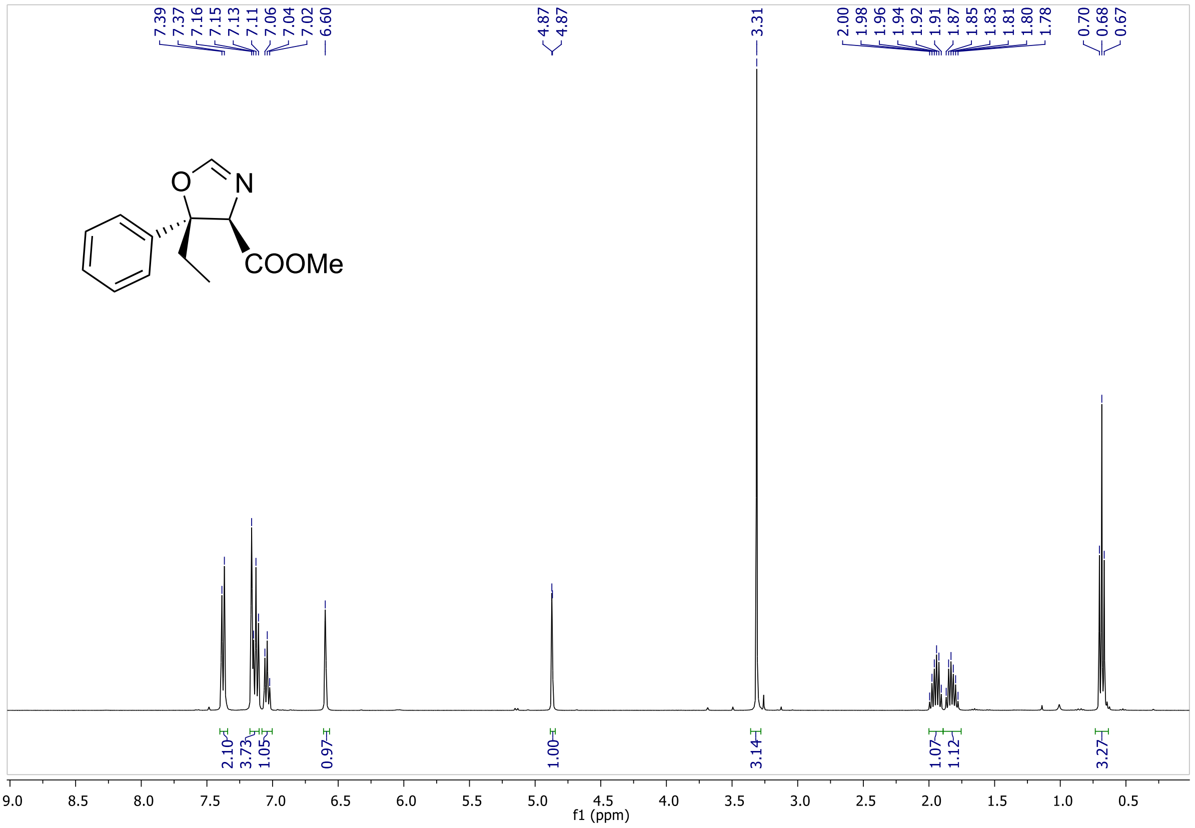

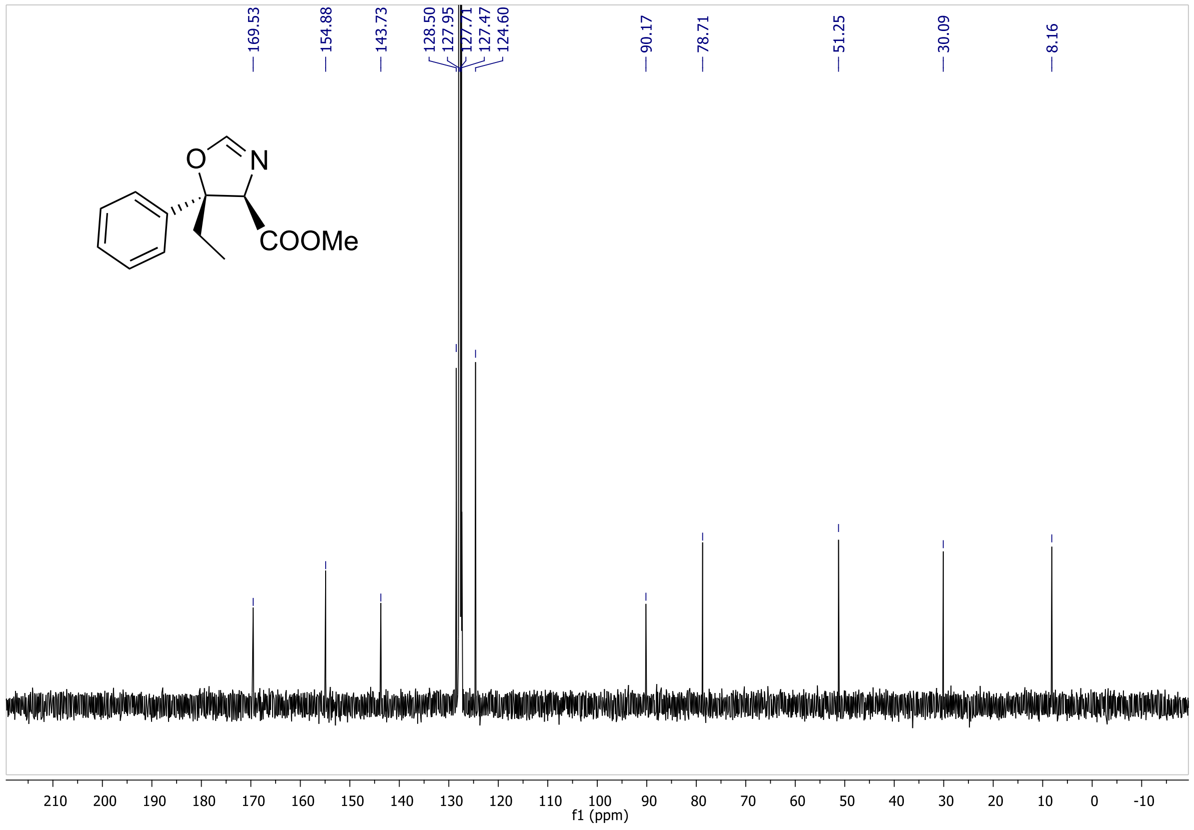

HPLC traces of **4t**

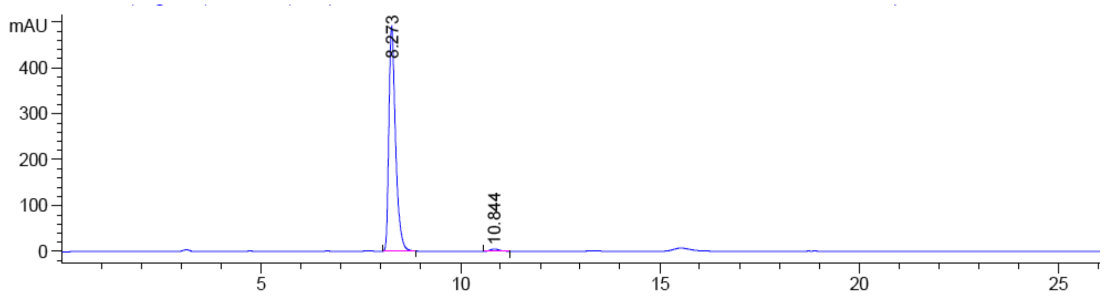

Peak #	RetTime [min]	Type	Width [min]	Area [mAU*s]	Height [mAU]	Area %
1	8.273	BB	0.1777	5690.38281	489.13629	98.7867
2	10.844	BB	0.2238	69.89135	4.80098	1.2133

HPLC traces of the enantiomer of **4t**

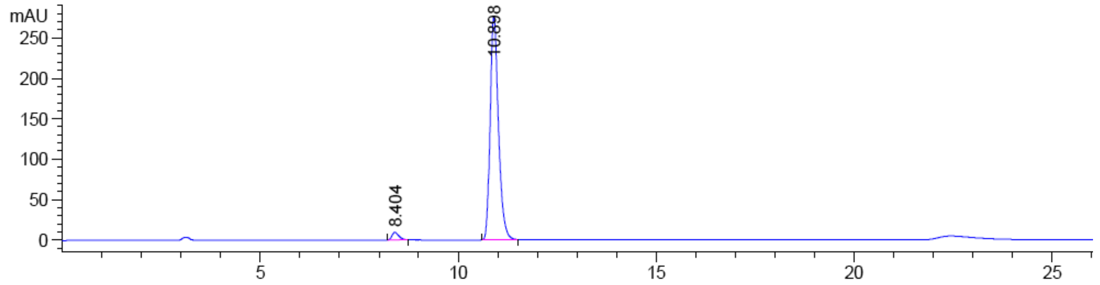

Peak #	RetTime [min]	Type	Width [min]	Area [mAU*s]	Height [mAU]	Area %
1	8.404	BB	0.1747	108.29958	9.37741	2.6268
2	10.898	BB	0.2216	4014.64526	276.17184	97.3732

HPLC traces of mixture of both enantiomers

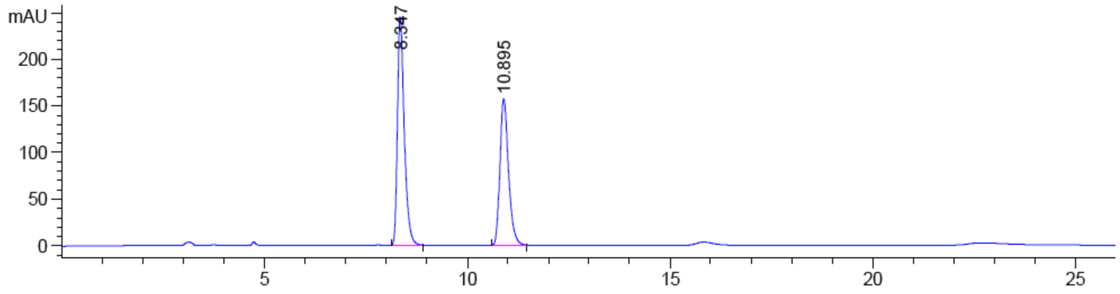

Peak #	RetTime [min]	Type	Width [min]	Area [mAU*s]	Height [mAU]	Area %
1	8.347	BB	0.1762	165.15140	14.35936	54.9991
2	10.896	BB	0.2251	135.12869	9.21488	45.0009

4.21 ^{1}H and ^{13}C-NMR spectra and HPLC traces of compound 4u

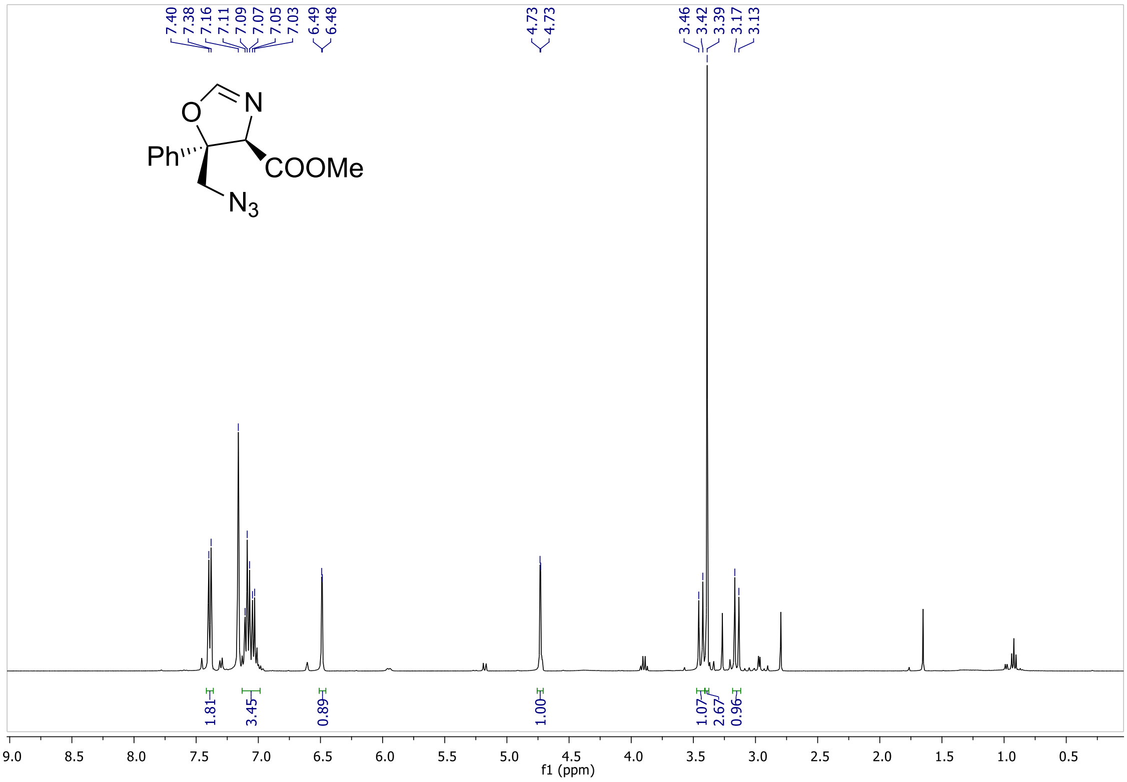

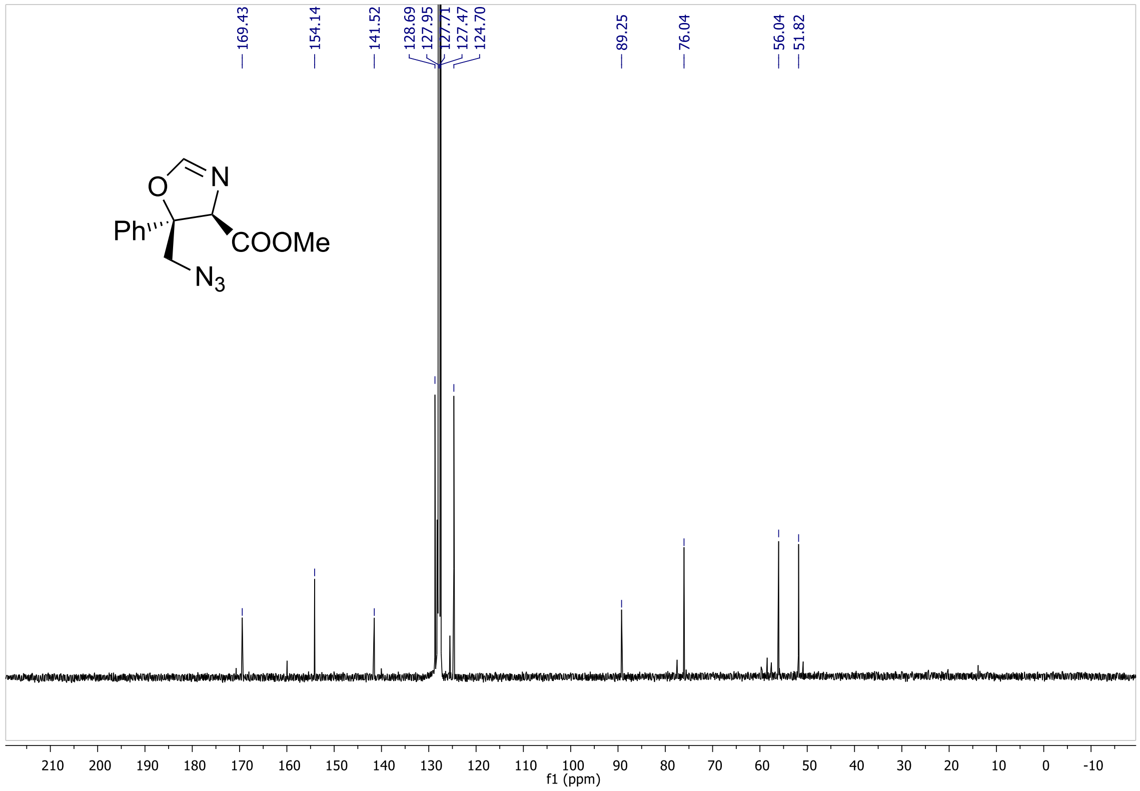

HPLC traces of racemic compound

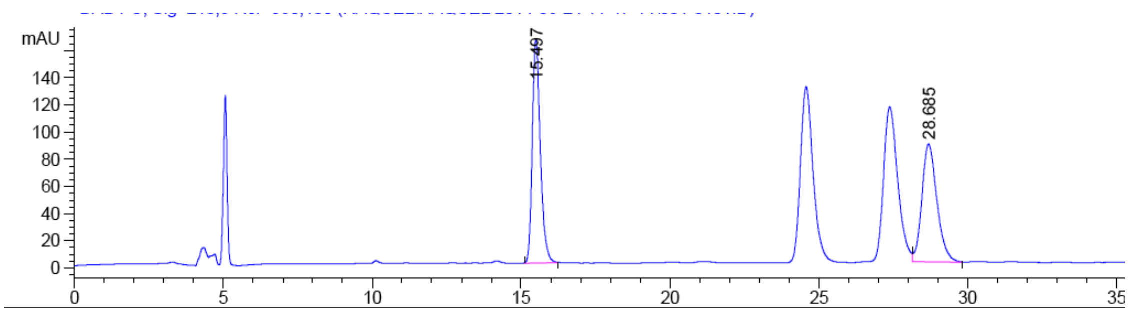

Peak #	RetTime [min]	Type	Width [min]	Area [mAU*s]	Height [mAU]	Area %
1	15.497	BB	0.2845	3086.36938	164.79169	50.3756
2	28.685	VB	0.5328	3040.34692	86.75761	49.6244

HPLC traces of **4u**

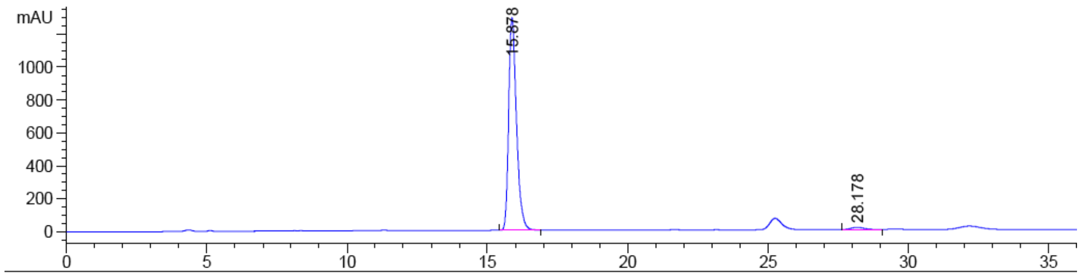

Peak #	RetTime [min]	Type	Width [min]	Area [mAU*s]	Height [mAU]	Area %
1	15.878	BB	0.2961	2.49750e4	1288.90234	98.4232
2	28.178	BB	0.4804	400.12769	12.81127	1.5768

HPLC traces of the enantiomer of **4u**

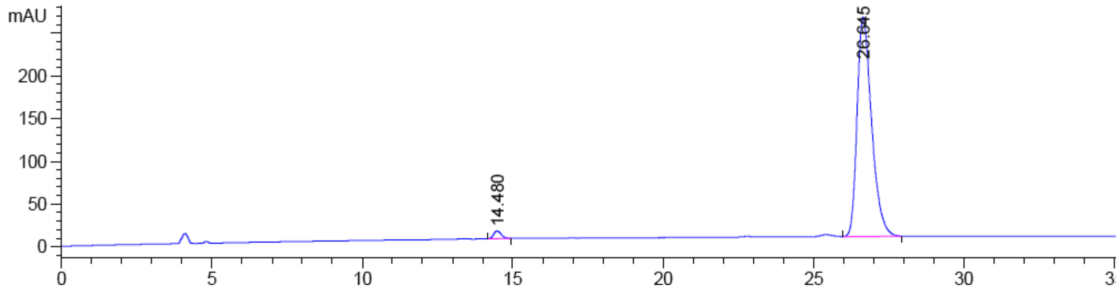

Peak #	RetTime [min]	Type	Width [min]	Area [mAU*s]	Height [mAU]	Area %
1	14.481	MM	0.3569	88.31874	4.12465	2.4299
2	26.645	VB	0.5111	3546.35156	105.79105	97.5701

4.22 ^{1}H and ^{13}C-NMR spectra and HPLC traces of compound 4v

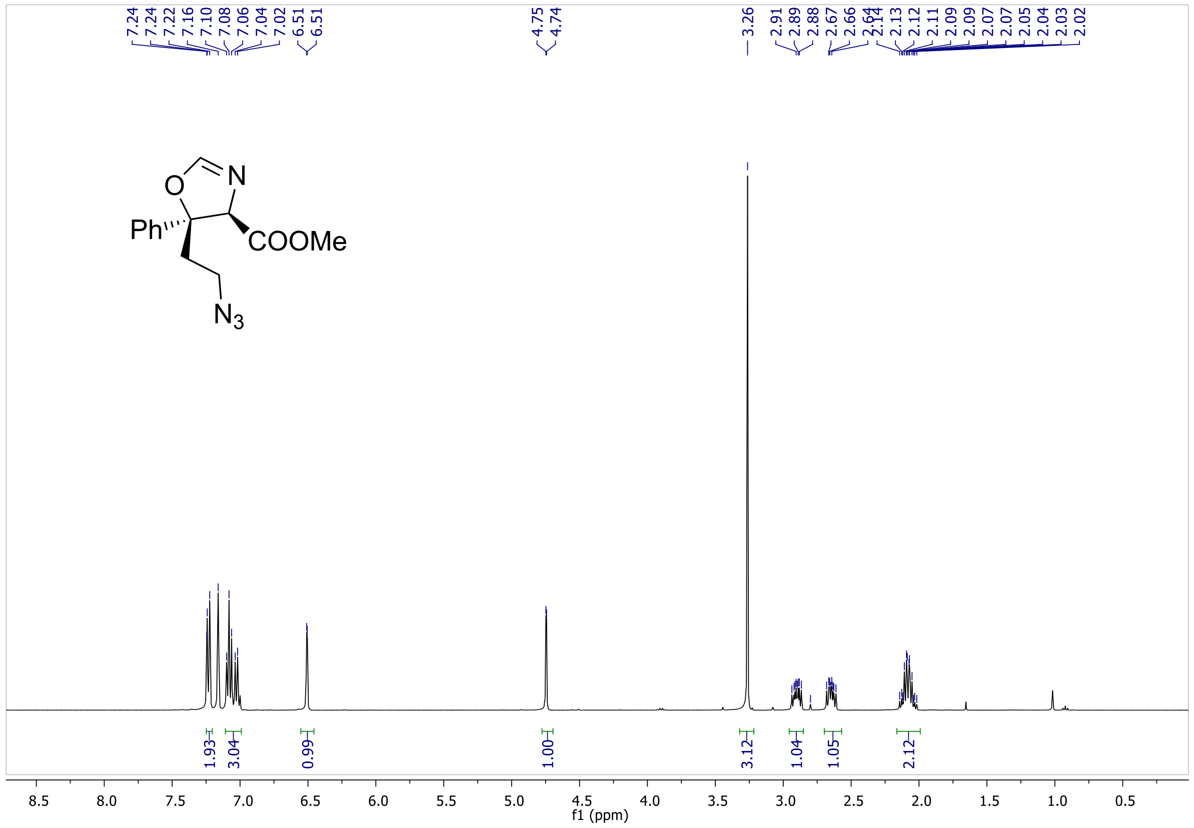

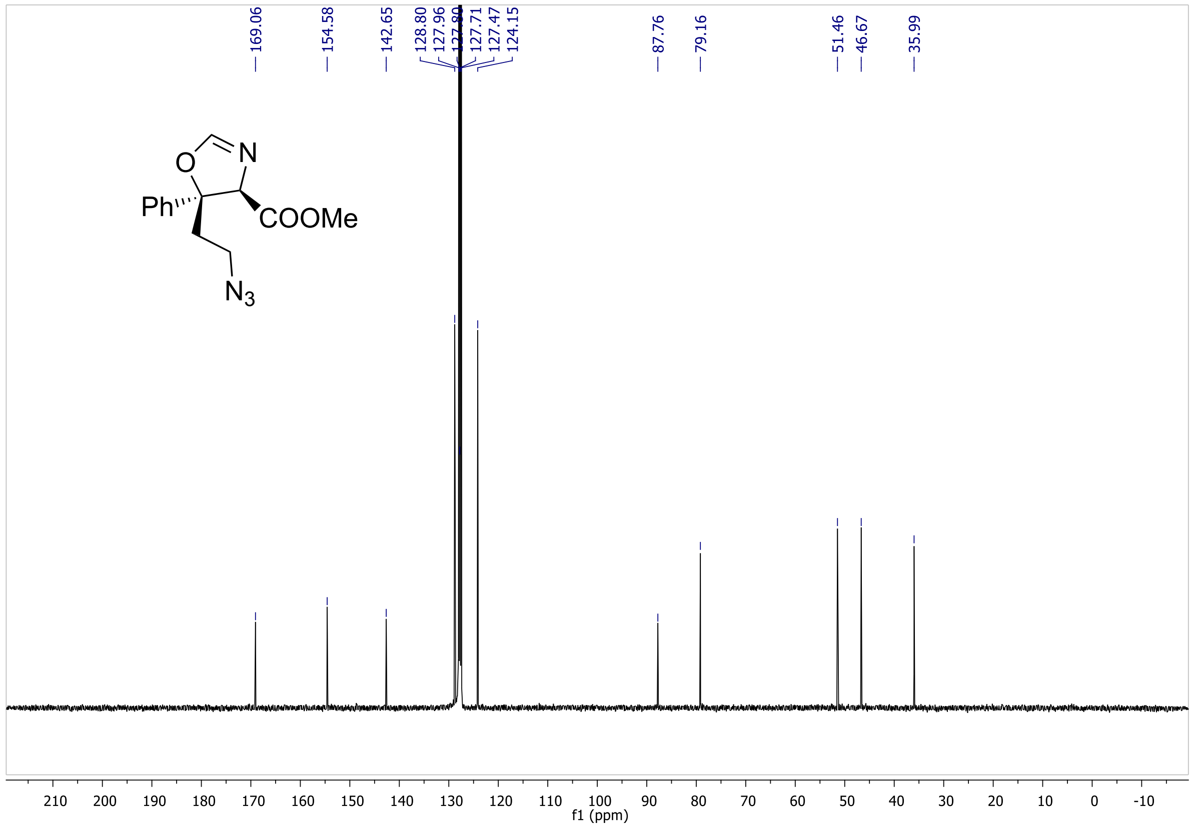

HPLC traces of of **4v**

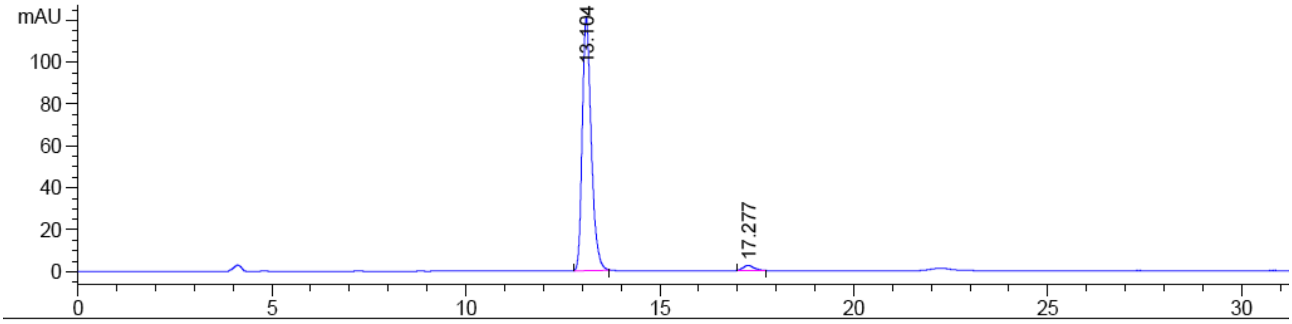

Peak #	RetTime [min]	Type	Width [min]	Area [mAU*s]	Height [mAU]	Area %
1	13.104	BB	0.2448	1946.58569	120.91148	97.3868
2	17.277	BB	0.2977	52.23353	2.52107	2.6132

HPLC traces of the enantiomer of **4v**

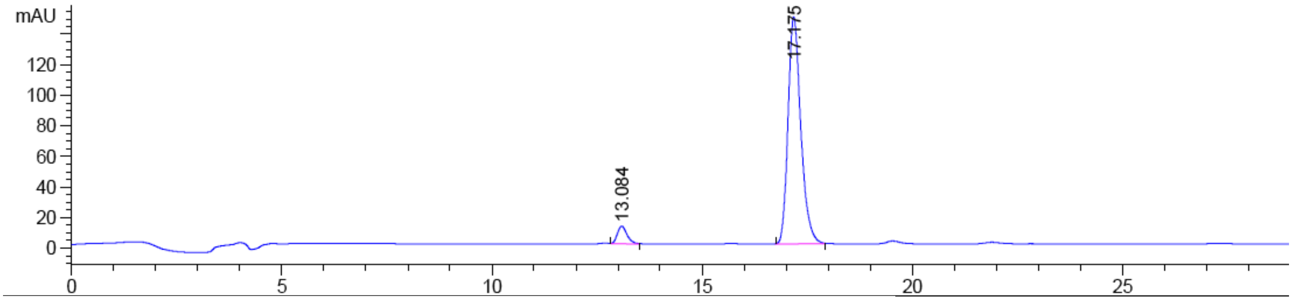

Peak #	RetTime [min]	Type	Width [min]	Area [mAU*s]	Height [mAU]	Area %
1	13.084	BB	0.2410	180.05991	11.41935	5.4079
2	17.175	BB	0.3230	3149.48975	148.73798	94.5921

HPLC traces of mixture of both enantiomers

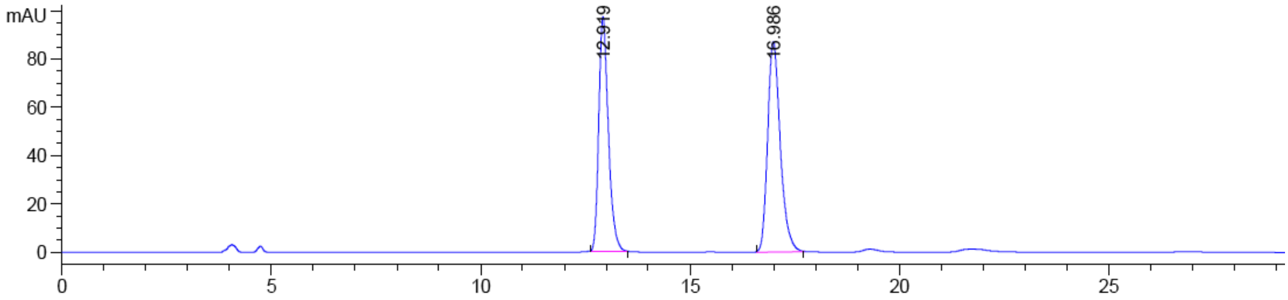

Peak #	RetTime [min]	Type	Width [min]	Area [mAU*s]	Height [mAU]	Area %
1	12.921	BB	0.2330	64.08192	4.22376	44.4948
2	16.982	BB	0.2893	79.93909	3.91295	55.5052

4.23 ^{1}H and ^{13}C-NMR spectra and HPLC traces of compound 5a

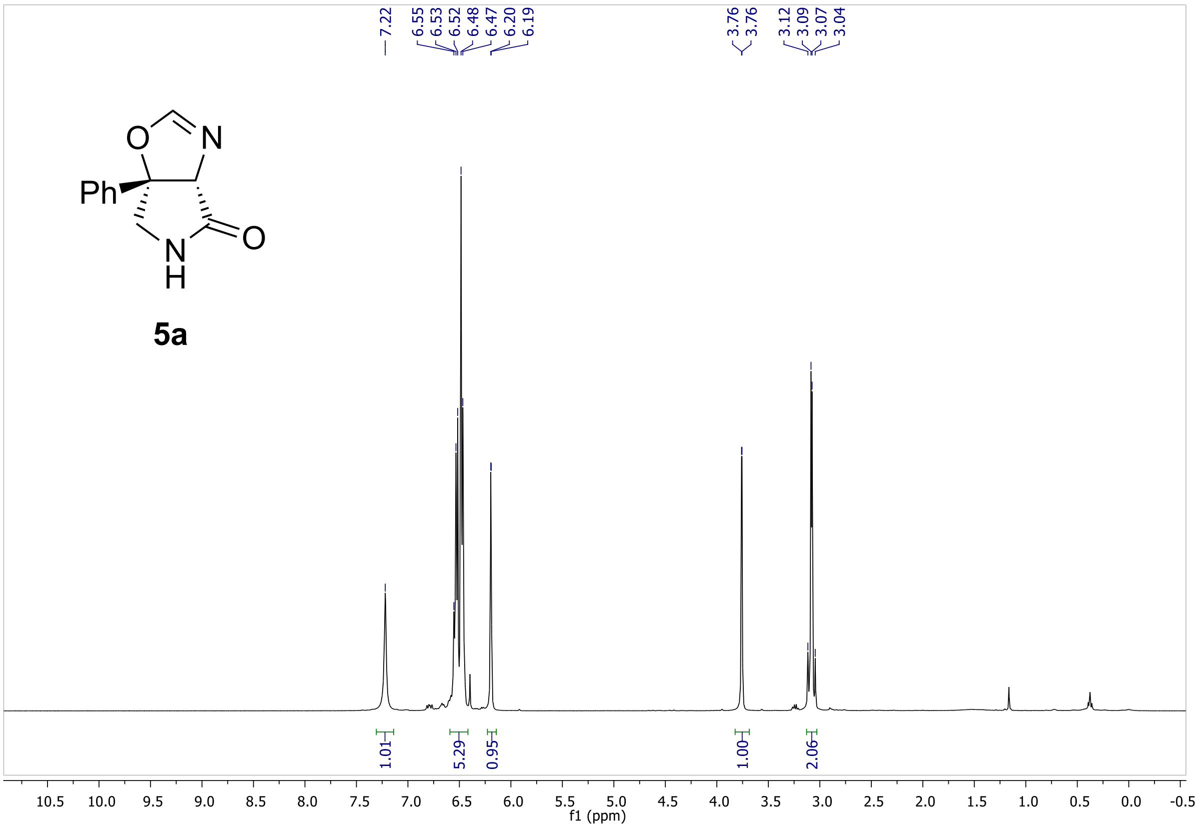

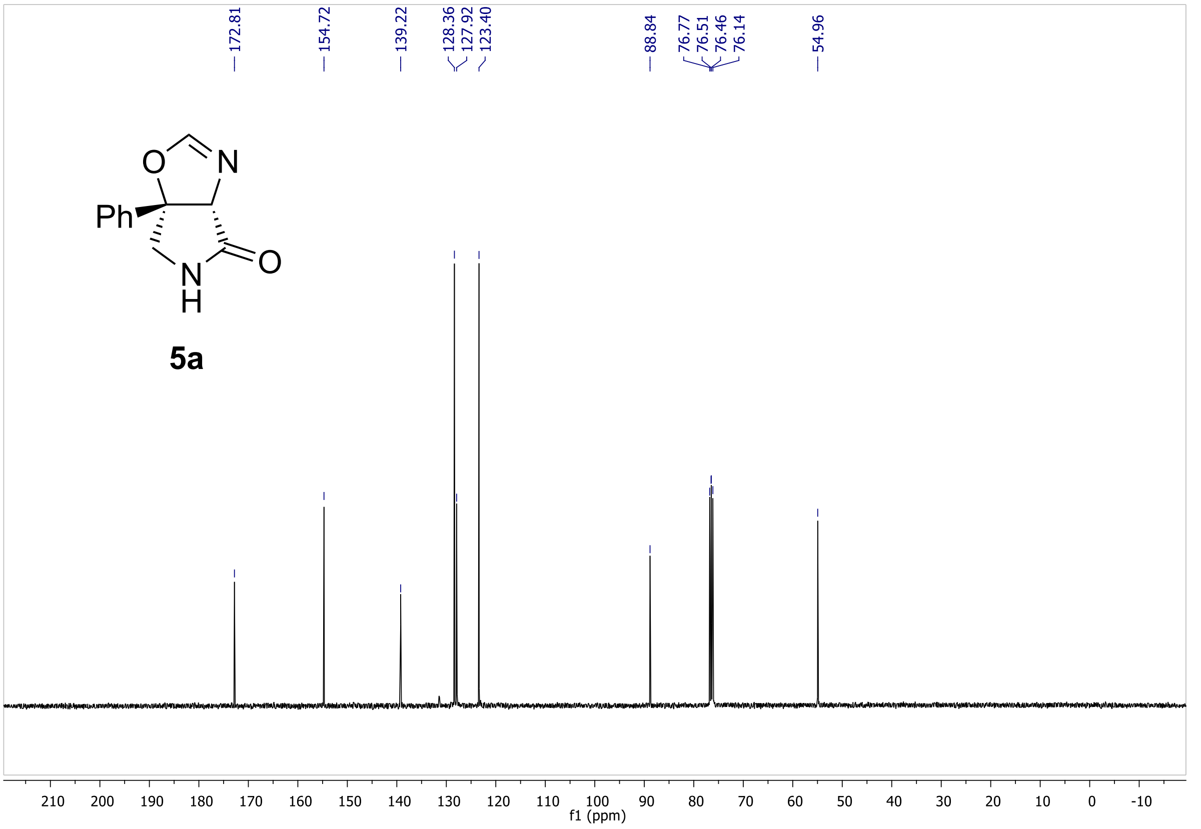

HPLC traces of of **5a**

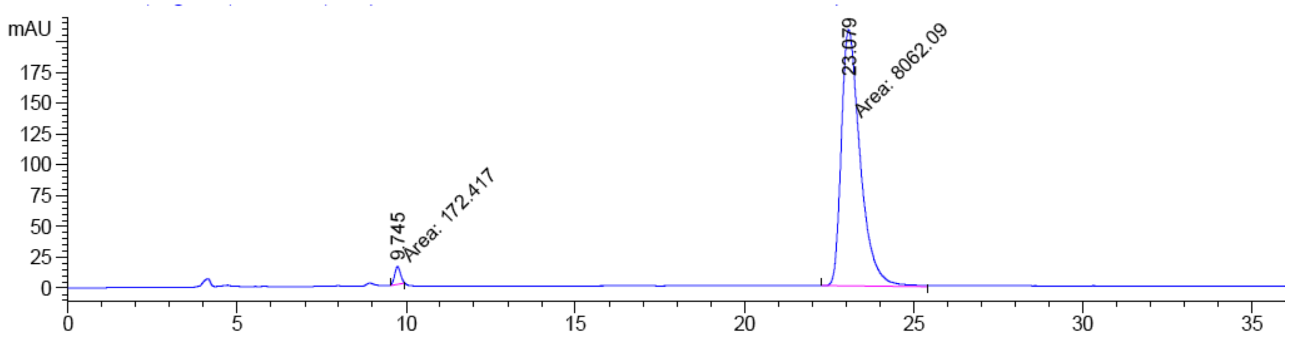

Peak #	RetTime [min]	Type	Width [min]	Area [mAU*s]	Height [mAU]	Area %
1	9.745	MM	0.1979	172.41728	14.52189	2.0938
2	23.079	MM	0.6471	8062.09473	207.65614	97.9062

HPLC traces of the enantiomer of **5a**

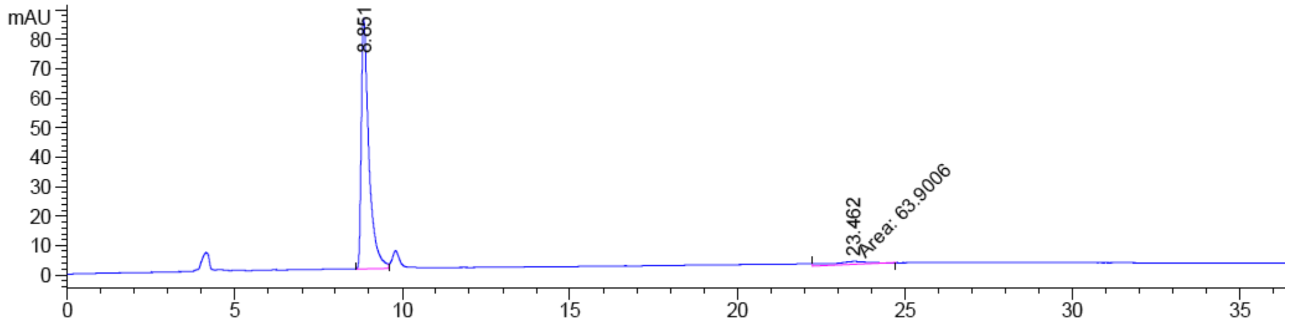

Peak #	RetTime [min]	Type	Width [min]	Area [mAU*s]	Height [mAU]	Area %
1	8.851	BB	0.2300	1342.28809	85.10344	95.4558
2	23.462	MM	1.2407	63.90058	8.58413e-1	4.5442

HPLC traces of mixture of both enantiomers

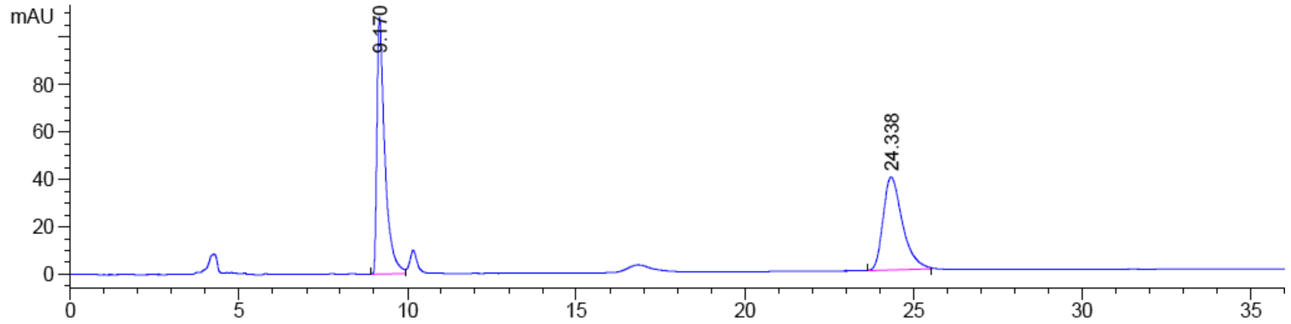

Peak #	RetTime [min]	Type	Width [min]	Area [mAU*s]	Height [mAU]	Area %
1	9.170	BV	0.2402	1761.40356	108.06648	52.9967
2	24.338	BB	0.6045	1562.20630	39.23745	47.0033

4.24 ^{1}H and ^{13}C-NMR spectra and HPLC traces of compound 5b

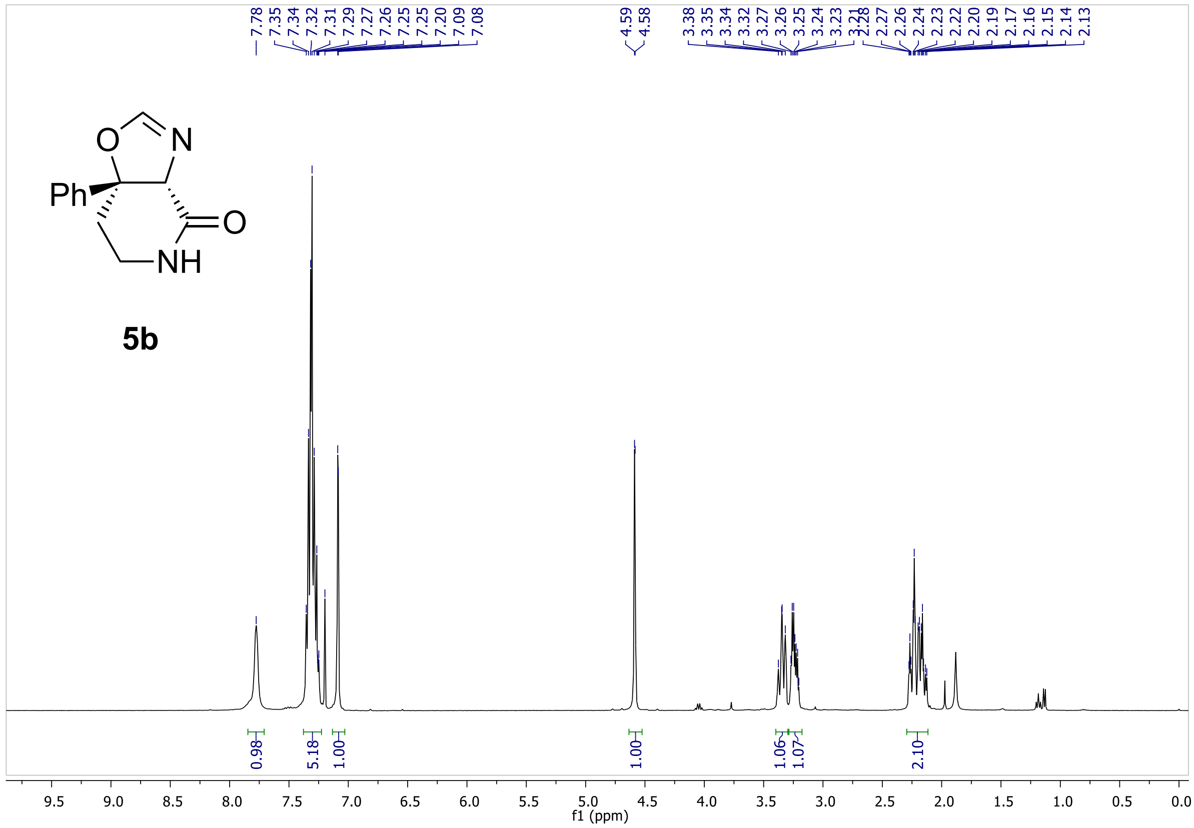

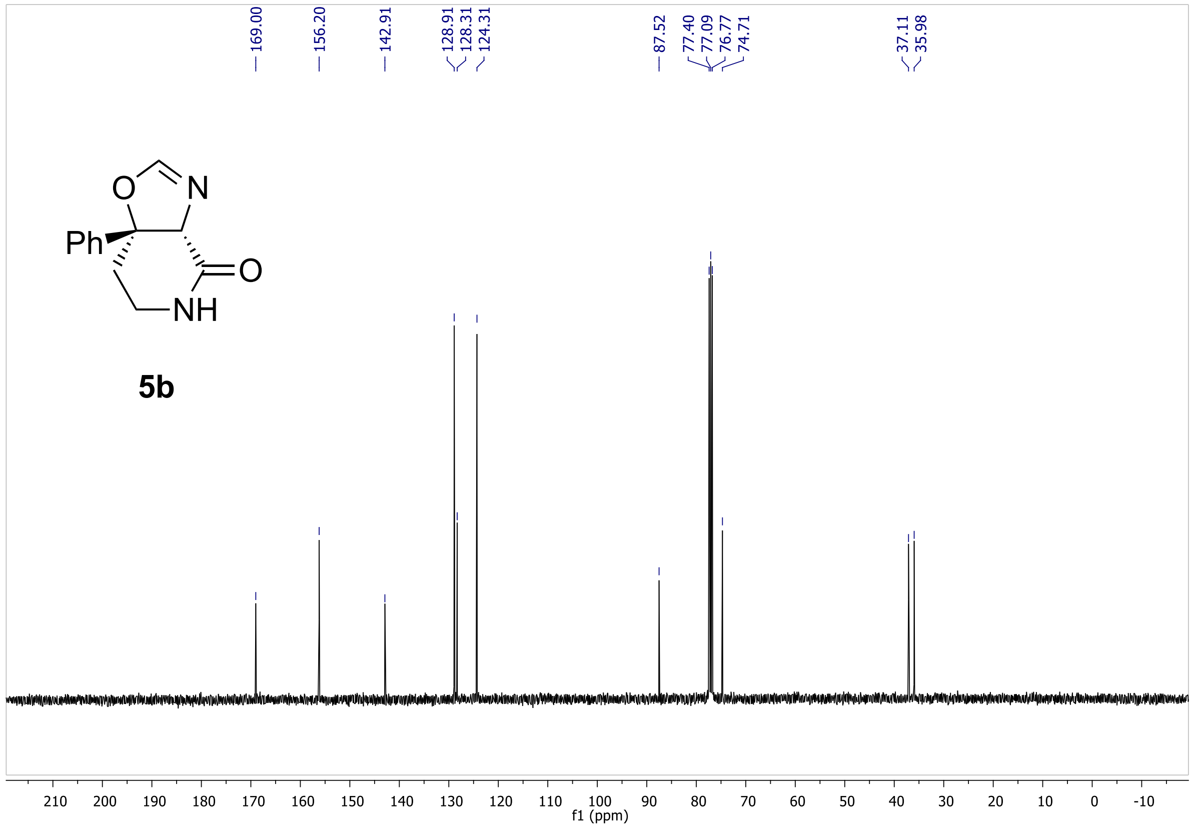

HPLC traces of of **5b**

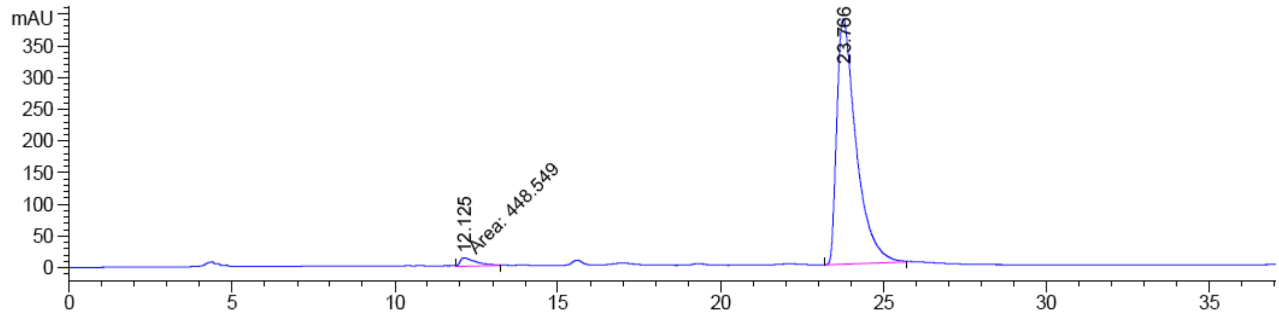

Peak #	RetTime [min]	Type	Width [min]	Area [mAU*s]	Height [mAU]	Area %
1	12.125	MM	0.5608	448.54941	13.33134	2.7296
2	23.766	BB	0.6087	1.59843e4	386.36707	97.2704

HPLC traces of the enantiomer of **5b**

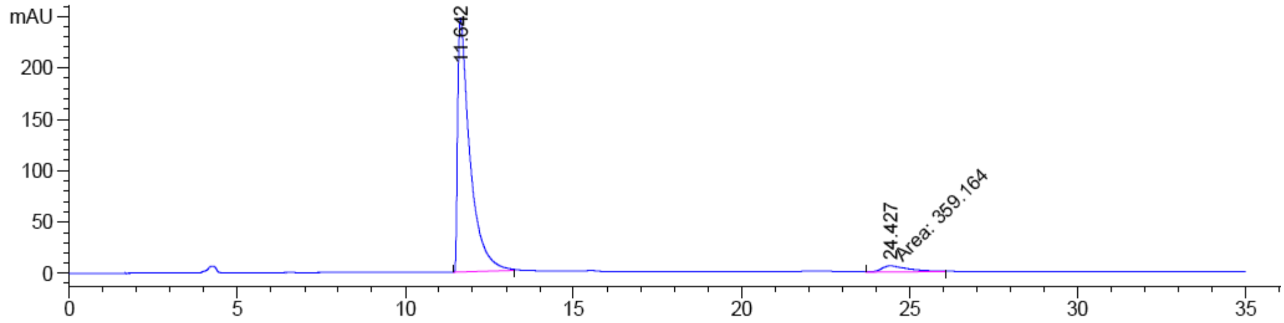

Peak #	RetTime [min]	Type	Width [min]	Area [mAU*s]	Height [mAU]	Area %
1	11.642	BB	0.3644	6287.49805	247.49672	94.5963
2	24.427	MM	0.9756	359.16354	6.13607	5.4037

HPLC traces of mixture of both enantiomers

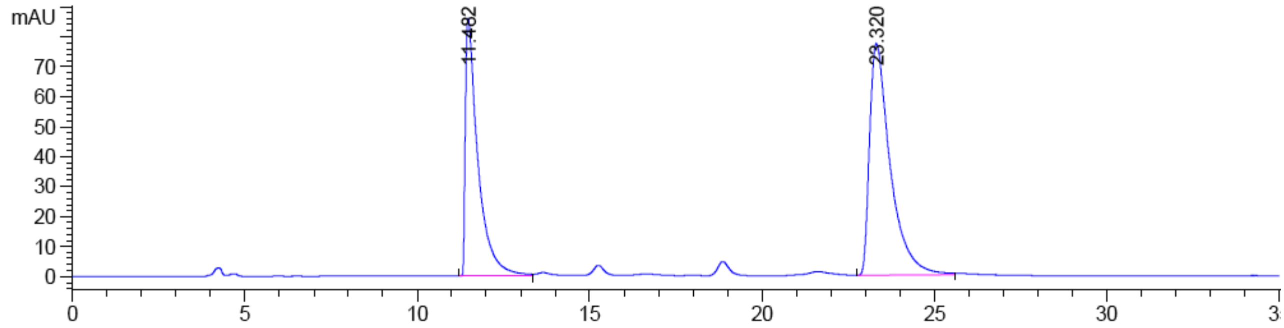

Peak #	RetTime [min]	Type	Width [min]	Area [mAU*s]	Height [mAU]	Area %
1	11.482	BV	0.3609	2184.88403	85.86687	40.4288
2	23.320	VB	0.6183	3219.39160	77.23882	59.5712

4.25 ^{1}H and ^{13}C-NMR spectra and HPLC traces of compound 6

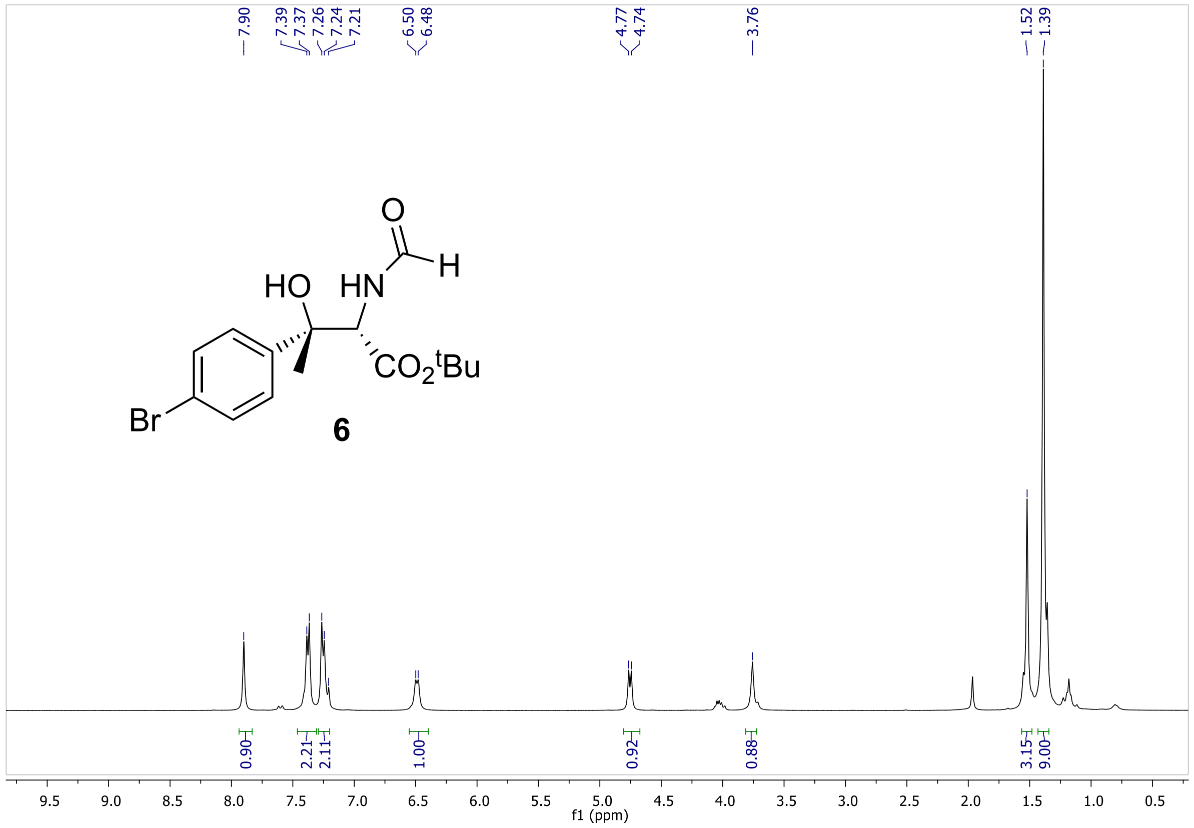

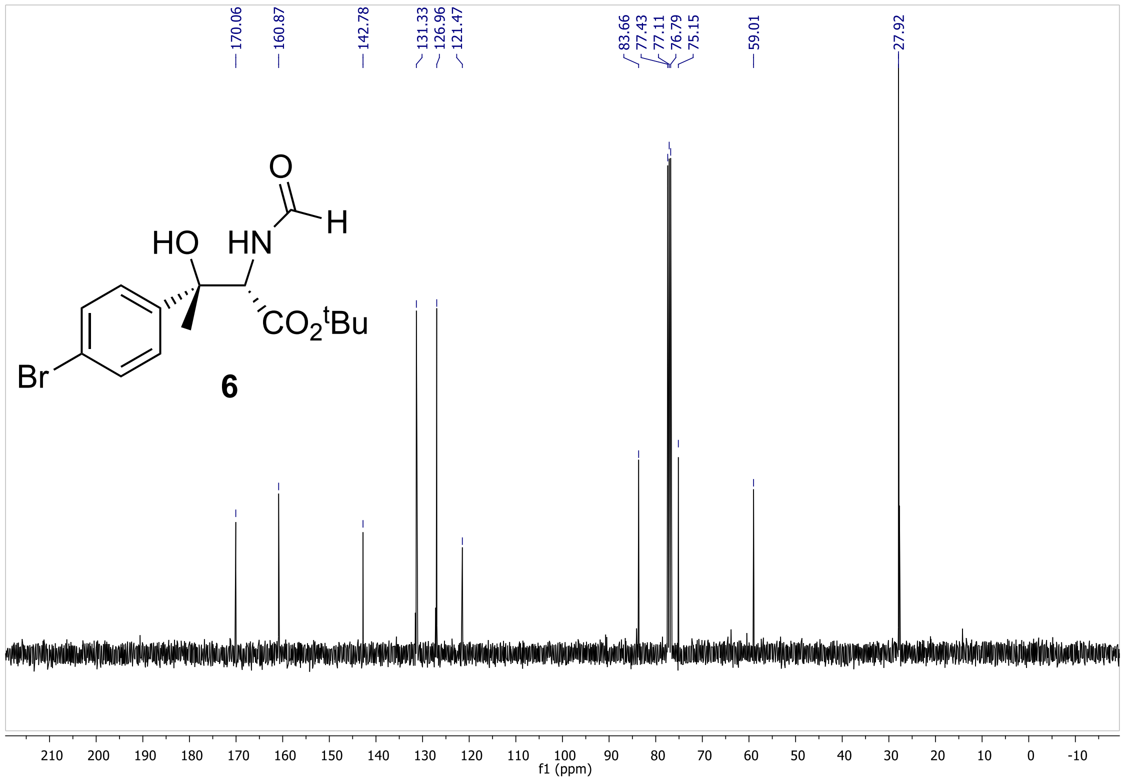

HPLC traces of racemic compound

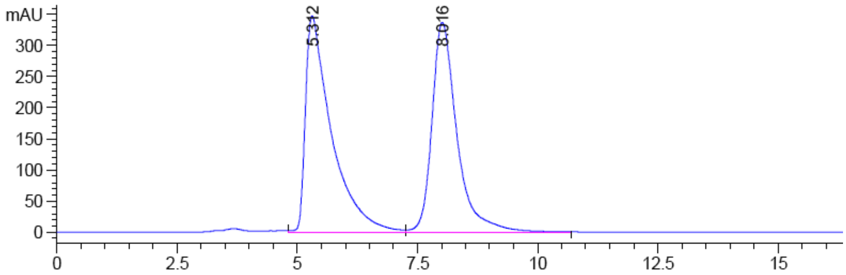

Peak #	RetTime [min]	Type	Width [min]	Area [mAU*s]	Height [mAU]	Area %
1	5.312	VV	0.5193	1.30780e4	347.28494	51.7679
2	8.016	VB	0.5383	1.21848e4	336.59964	48.2321

HPLC traces of **6**

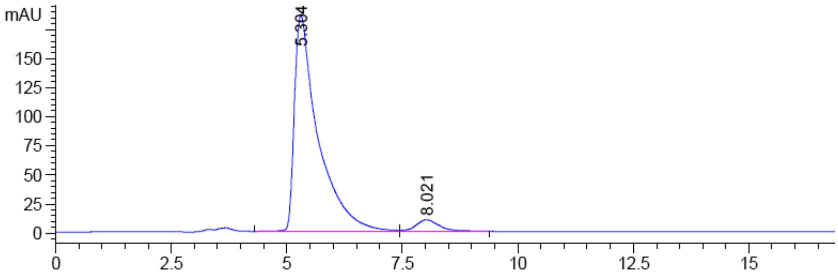

Peak #	RetTime [min]	Type	Width [min]	Area [mAU*s]	Height [mAU]	Area %
1	5.304	VV	0.5088	6702.44043	185.81700	94.6211
2	8.021	VB	0.5571	381.01132	10.17069	5.3789

4.26 ^{1}H and ^{13}C-NMR spectra and HPLC traces of compound 7

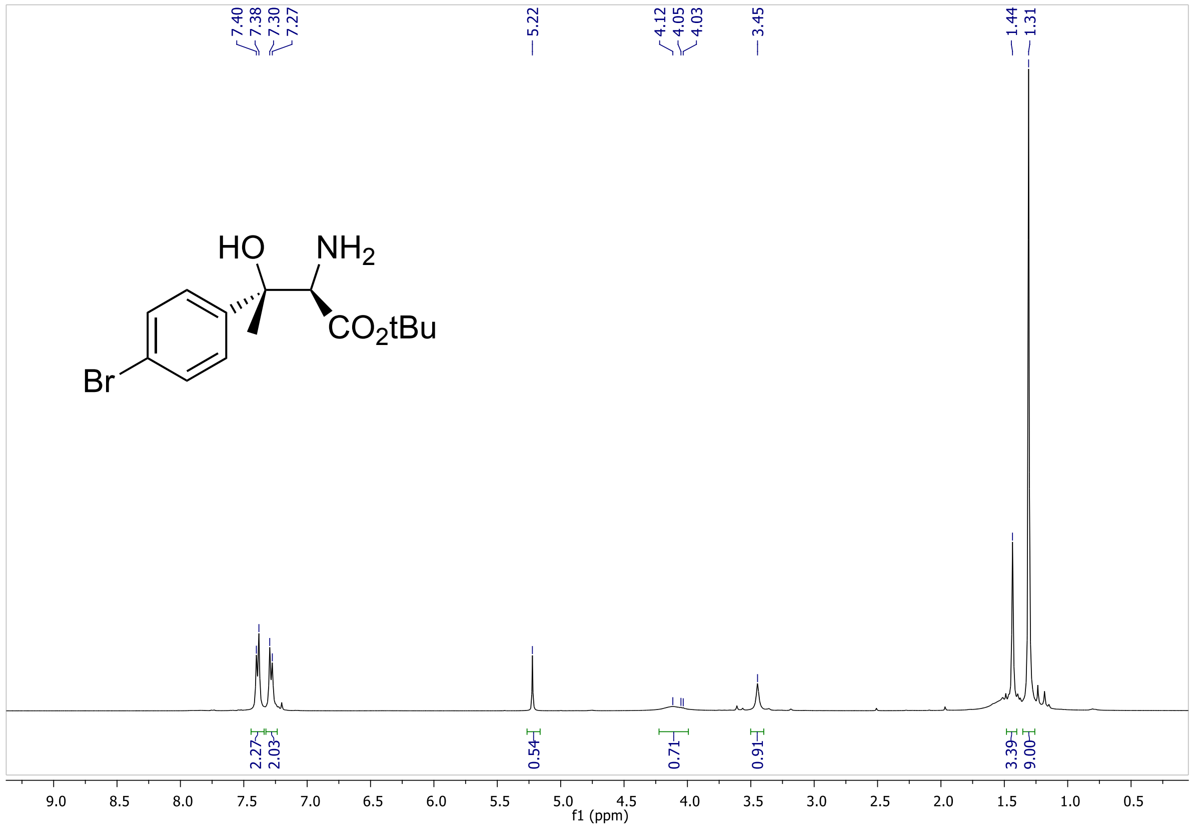

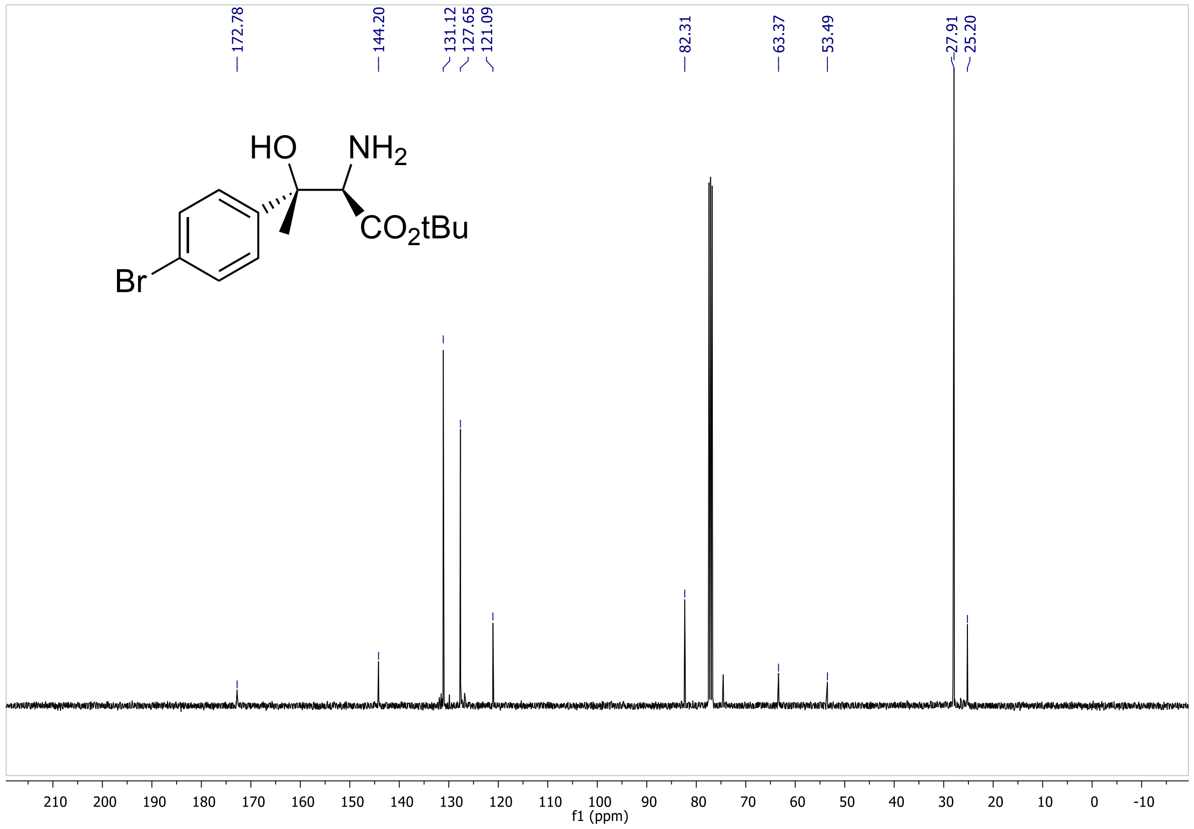

4.27 ^{1}H and ^{13}C-NMR spectra and HPLC traces of compound 8

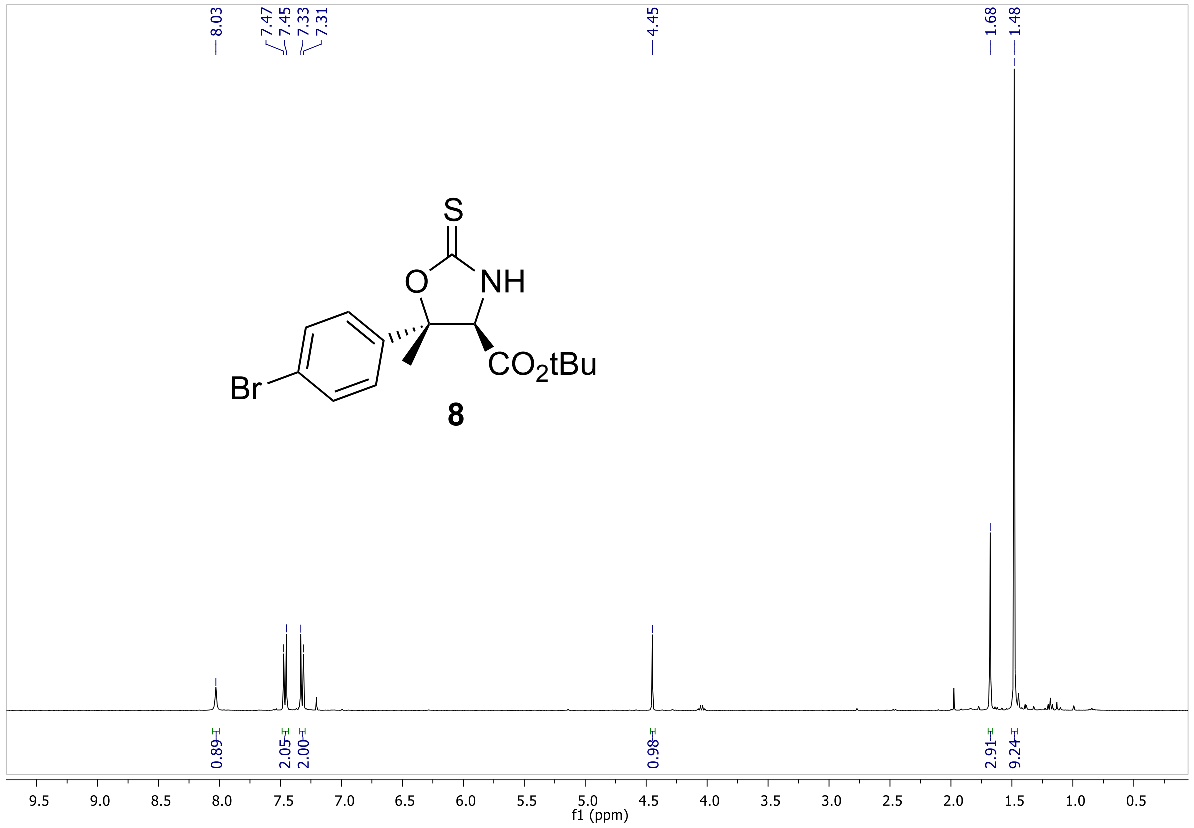

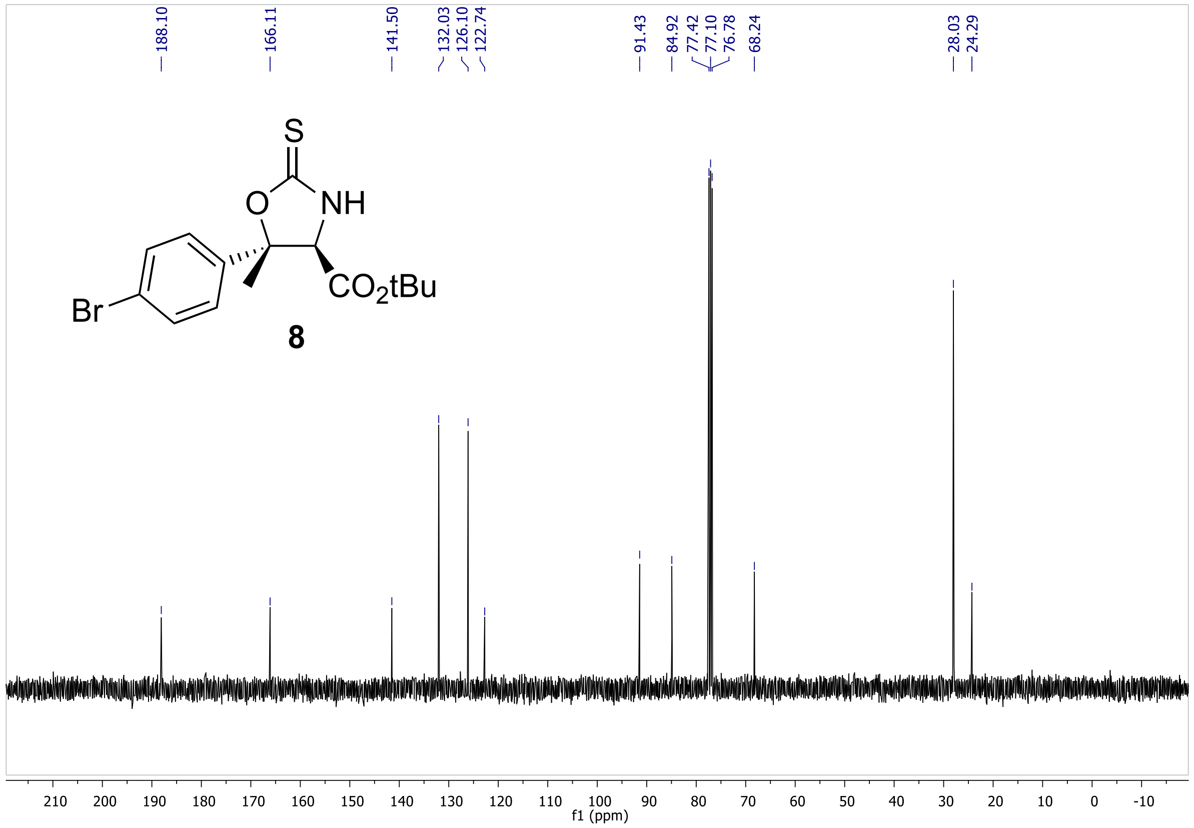

HPLC traces of racemic compound

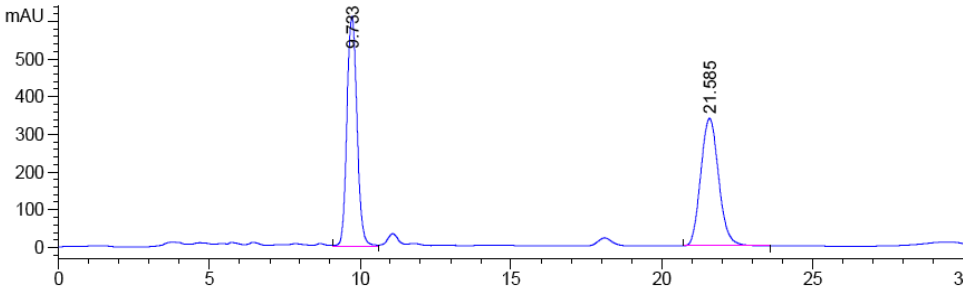

Peak #	RetTime [min]	Type	Width [min]	Area [mAU*s]	Height [mAU]	Area %
1	9.733	VV	0.3728	1.42701e4	610.20959	50.3676
2	21.585	BB	0.6522	1.40618e4	338.82504	49.6324

Before recrystalization

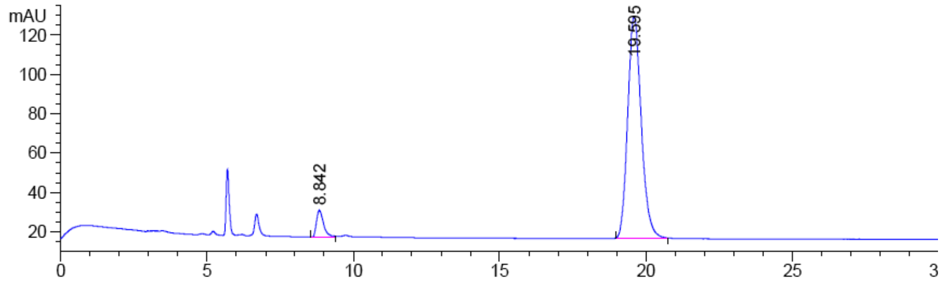

Peak #	RetTime [min]	Type	Width [min]	Area [mAU*s]	Height [mAU]	Area %
1	8.842	BB	0.2564	221.97612	13.46174	5.7443
2	19.595	BB	0.5008	3642.29297	112.76889	94.2557

After recrystalization

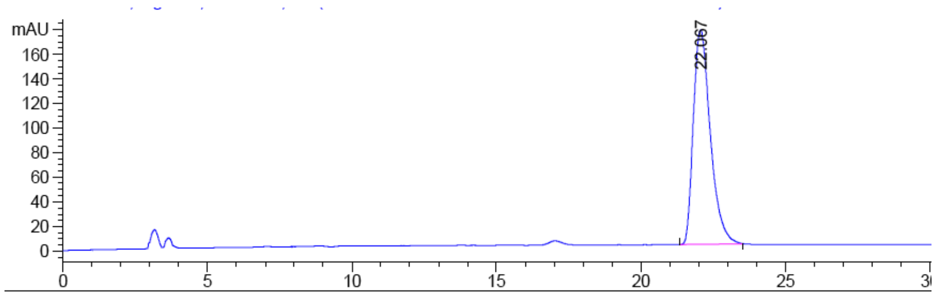

Peak #	RetTime [min]	Type	Width [min]	Area [mAU*s]	Height [mAU]	Area %
1	22.067	BB	0.6358	7138.50342	174.28699	100.0000

5. X-Ray data of compound 8

Low temperature single X-ray diffraction data were collected for **8** using a Nonius KCCD diffractometer. Data were reduced using DENZO-SMN[5] and solved using SIR92.[6] Structures were refined using CRYSTALS[7]. For **8**, the Flack x parameter[8] refined to 0.015(5). Full crystallographic data (in CIF format) is available as ESI and has been deposited with the Cambridge Crystallographic Data Centre (reference code **CCDC 1036558**).

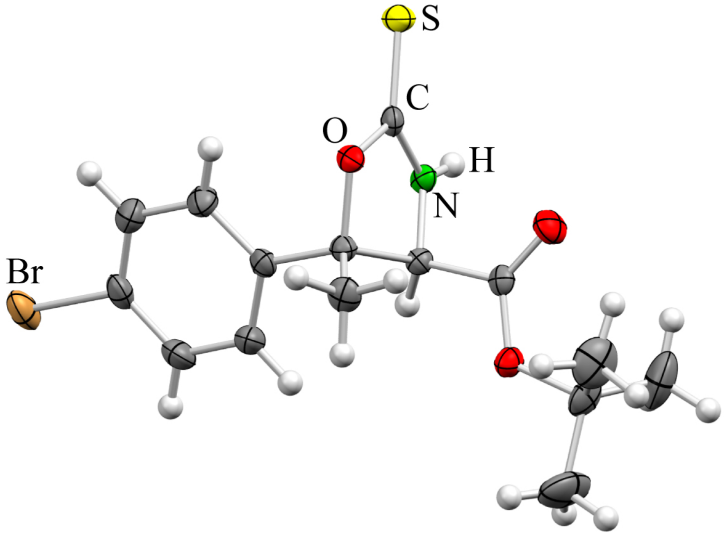

Figure S1. XRD molecular structure of compound **8** (thermal ellipsoids set at 60 % probability).

6. ^{1}H-NMR and ^{13}C-NMR spectra and HPLC traces of compound 4x, 4y and 4z.

6.1 Synthesis and characterization of *tert*-butyl 5,5-diethyl-4,5-dihydrooxazole-4-carboxylate 4x.

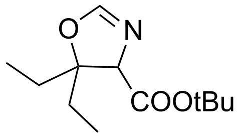

The general procedure was followed. The desired product was obtained as a colorless oil in 32% yield (25 mg); The er was determinated by HPLC using a Chiralpack OD-H [hexane/iso-propanol 90:10, λ 220, 1 mL/min] t (minor) = 4.48 min., t (major) = 5.35 min. (61:39). $[\alpha]_D^{20}$ = +4.96 (*c* 0.5, $CHCl_3$); ^{1}H

---

[5] Otwinowski & Minor, Processing of X-ray Diffraction Data Collected in Oscillation Mode, Methods Enzymol. **1997**, 276, Eds C. W. Carter, R. M. Sweet, Academic Press.

[6] A. Altomare, G. Cascarano, C. Giacovazzo, A. Guagliardi, M. C. Burla, G. Polidori & M. Camalli, *J. Appl. Cryst.* **1994**, *27*, 435

[7] P. W. Betteridge, J. R. Carruthers, R. I. Cooper, K. Prout, & D. J. Watkin, *J. Appl. Cryst*. **2003**, *36*, 1487. b) R. I. Cooper, A. L. Thompson & D. J. Watkin, *J. Appl. Cryst*. **2010**, *43*, 1100-1107

[8] a) H. D. Flack, *Acta Cryst*. **1983**, *A39*, 876-881. b) H. D. Flack & G. Bernardinelli, *J. Appl. Cryst*. **2000**, *33*, 1143-1148.

NMR (400 MHz, C_6D_6) δ 0.69 (t, *J* = 7.5 Hz, 3H), 0.84 (t, *J* = 7.5 Hz, 3H), 1.31 (s, 9H), 1.37-1.58 (m, 2H), 1.60-1.71 (m, 1H), 1.74-1.88 (m, 1H), 4.37 (d, *J* = 2.0 Hz, 1H), 6.51 (d, *J* = 2.0 Hz, 1H); ^{13}C NMR (100 MHz, C_6D_6) δ 6.9 (CH_3), 7.9 (CH_3), 25.9 (CH_2), 27.5 (3xCH_3), 30.0 (CH_2), 74.7 (CH), 80.7 (C), 89.5 (C), 155.0 (CH), 168.6 (C); IR ν_{max}/cm^{-1} 1730, 1668, 1369, 1155; HRMS (ES) calcd $C_{12}H_{21}NO_3$ (M^+) 227.1521, found 227.1527.

6.2 Synthesis and characterization of *tert*-butyl 5,5-dimethyl-4,5-dihydrooxazole-4-carboxylate 4y. The general procedure was followed. The desired product was obtained as a colorless oil in 40% yield (28 mg); The er was determined by HPLC using a Chiralpack OD-H [hexane/iso-propanol 90:10, λ 220, 1 mL/min] t (minor) = 4.89 min., t (major) = 6.12 min. (47:53). $[\alpha]_D^{20}$ = +12.0 (*c* 0.5, $CHCl_3$); ^{1}H NMR (400 MHz, C_6D_6) δ 1.12 (s, 3H), 1.25 (s, 3H), 1.29 (s, 9H), 4.24 (d, *J* = 2.0 Hz, 1H), 6.50 (d, *J* = 2.0 Hz, 1H); ^{13}C NMR (100 MHz, C_6D_6) δ 22.3 (CH_3), 27.5 (3xCH_3), 28.3 (CH_3), 76.7 (CH), 80.8 (C), 84.2 (C), 155.1 (CH), 168.4 (C); IR ν_{max}/cm^{-1} 1738, 1368, 1216, 1104; HRMS (ES) calcd $C_{10}H_{17}NNaO_3$ ($M+Na^+$) 222.1101, found 222.1103.

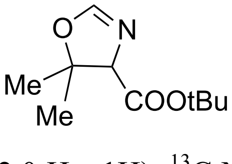

6.3 Synthesis and characterization of (4*R*,5*S*)-*tert*-butyl 5-cyclohexyl-5-methyl-4,5-dihydrooxazole-4-carboxylate 4z. The general procedure was followed. The desired product was obtained as a colorless oil in 78% yield (70 mg, trans:cis=85:15; data for the trans diastereoisomer); The er was determined by HPLC using a Chiralpack AD-H [hexane/iso-propanol 95:5, λ 220, 1 mL/min] t (minor) = 12.76 min., t (major) = 13.62 min. (67:33). $[\alpha]_D^{20}$ = - 18.5 (*c* 0.68, $CHCl_3$); ^{1}H NMR (400 MHz, C_6D_6) δ 0.73-0.85 (m, 1H), 1.01-1.03 (m, 2H), 1.29 (s, 3H), 1.32 (s, 9H), 1.51-1.76 (m, 6H), 4.46 (d, *J* = 2.0 Hz, 1H), 6.53 (d, *J* = 2.0 Hz, 1H); ^{13}C NMR (100 MHz, C_6D_6) δ 18.4 (CH_3), 26.1 (CH_2), 26.2 (CH_2), 26.4 (CH_2), 26.6 (CH_2), 27.6 (3xCH_3),47.7 (CH), 73.8 (CH), 80.6 (C), 89.0 (C), 154.8 (CH), 168.9 (C); HRMS (ES) calcd $C_{15}H_{26}NO_3$ $[M+H]^+$ 268.1907, found 268.1906.

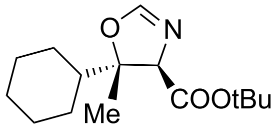

6.4 ^{1}H and ^{13}C-NMR spectra and HPLC traces of compound 4x

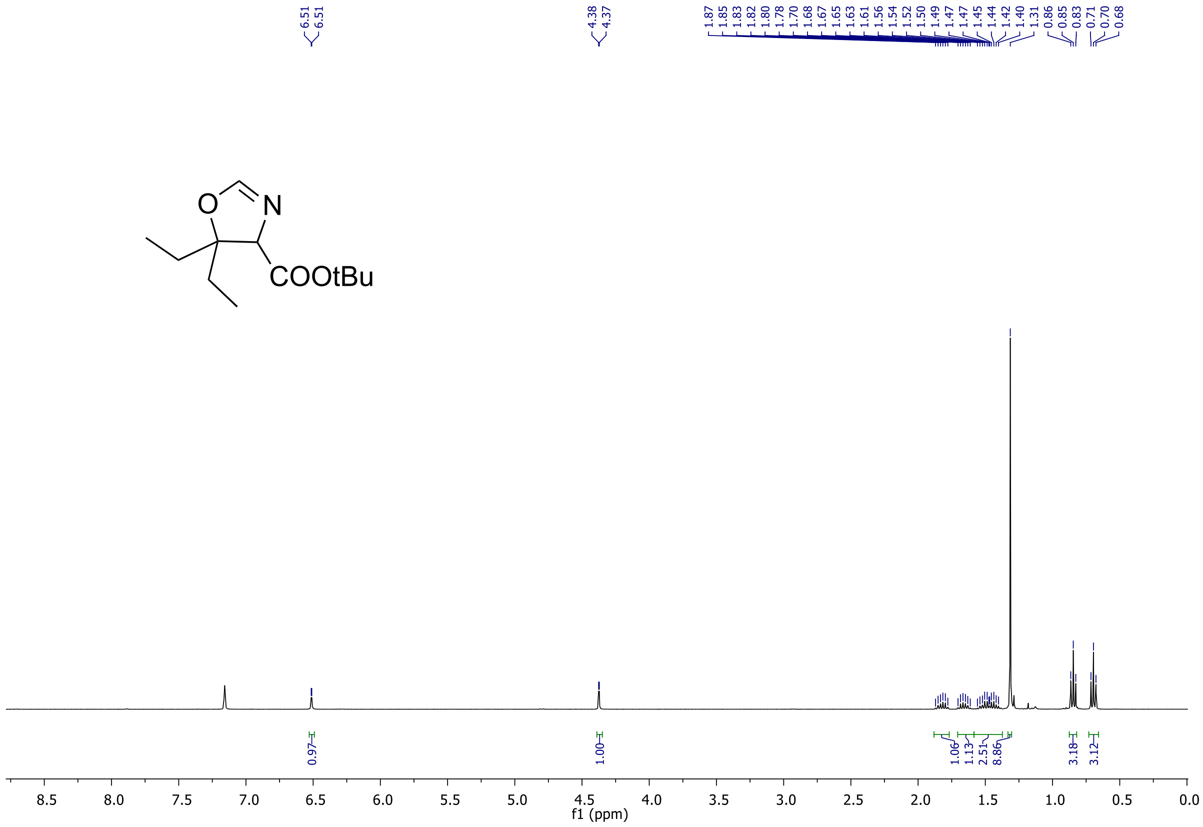

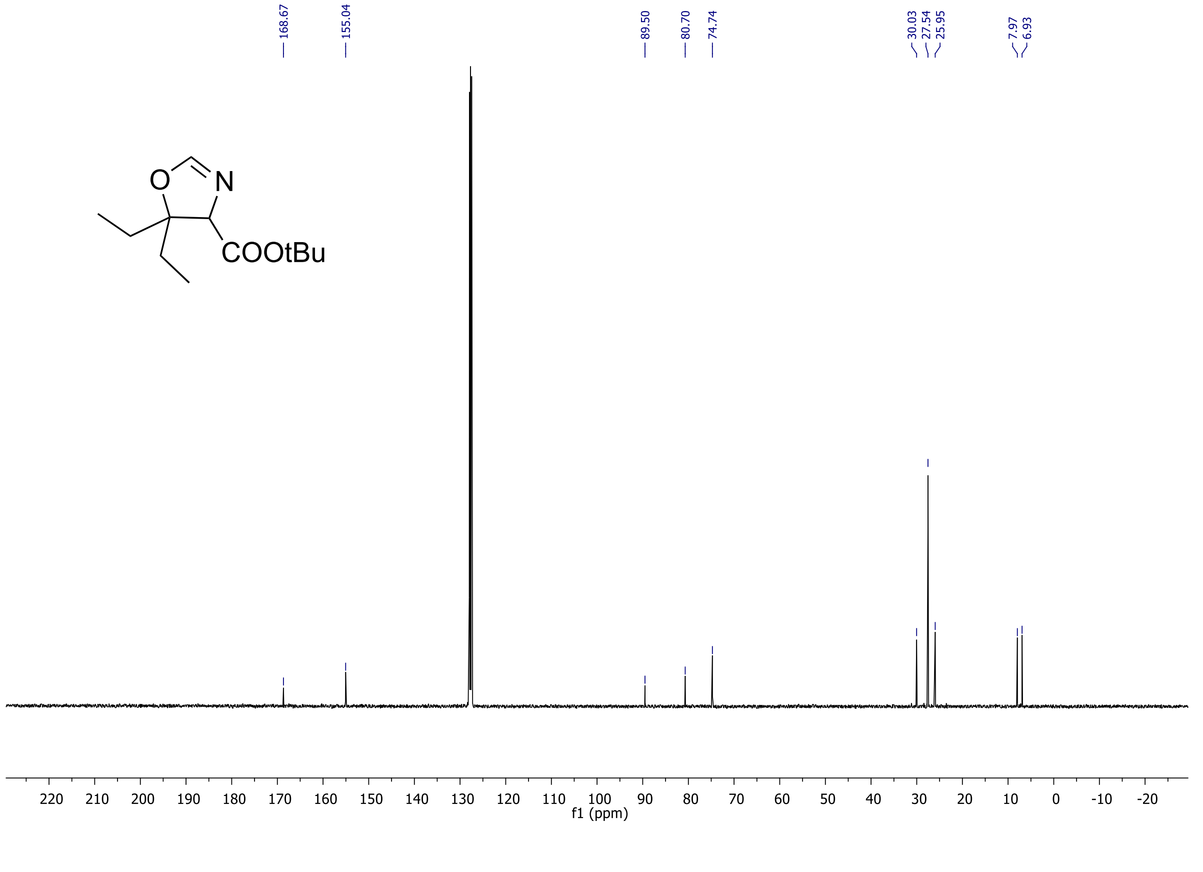

HPLC traces of racemic product

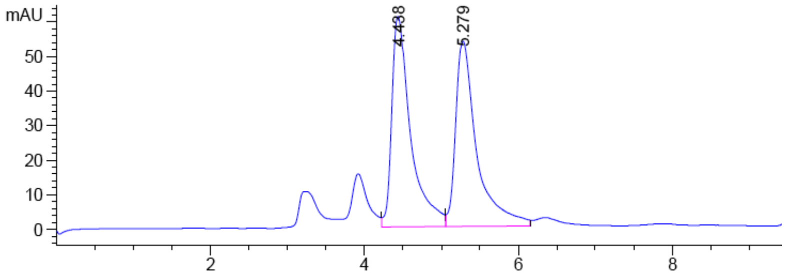

Peak #	RetTime [min]	Type	Width [min]	Area [mAU*s]	Height [mAU]	Area %
1	4.438	VV	0.2468	1028.21252	60.94788	50.3448
2	5.279	VB	0.2766	1014.13007	53.62664	49.6552

HPLC traces of **4x**

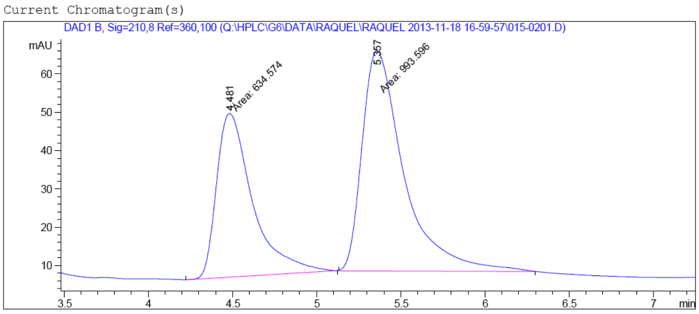

Signal 2: DAD1 B, Sig=210,8 Ref=360,100

Peak #	RetTime [min]	Type	Width [min]	Area [mAU*s]	Height [mAU]	Area %
1	4.481	MM	0.2473	634.57391	42.76239	38.9747
2	5.357	MM	0.2879	993.59576	57.51864	61.0253
Totals :				1628.16968	100.28104	

6.5 ^{1}H and ^{13}C-NMR spectra and HPLC traces of compound 4y

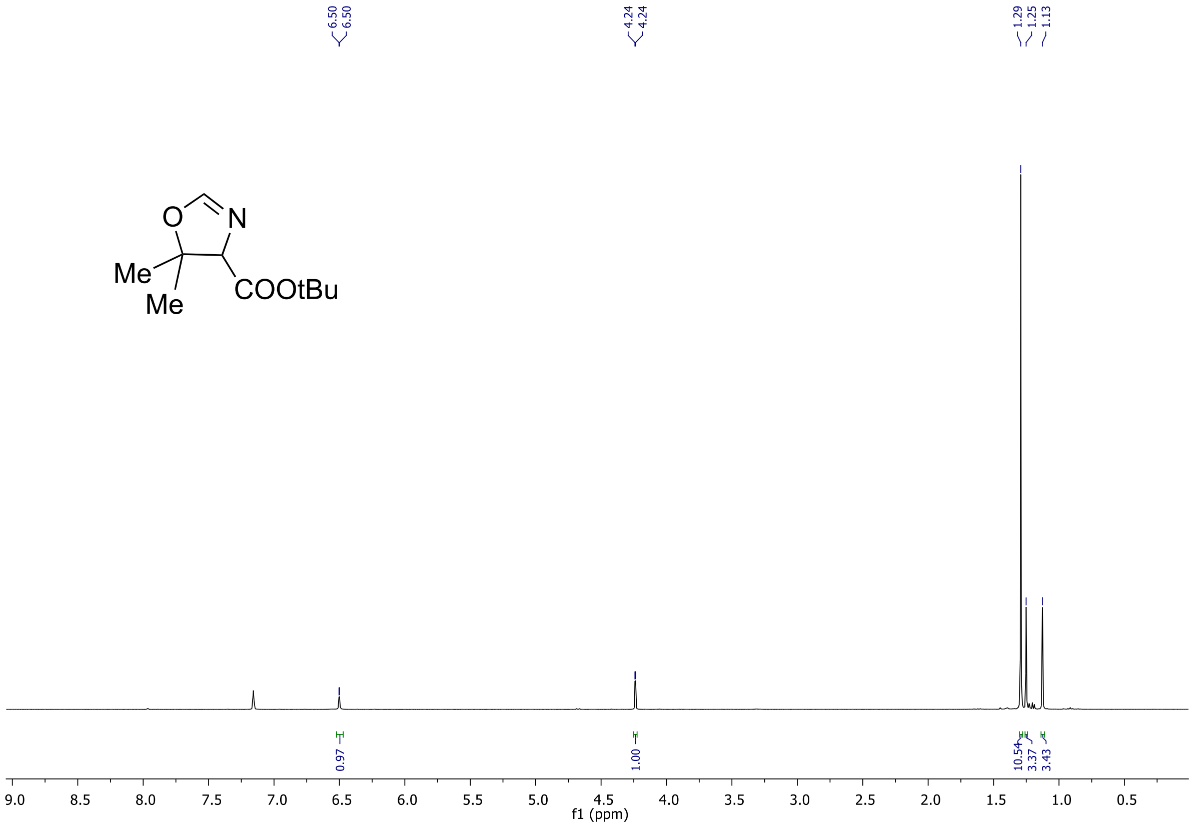

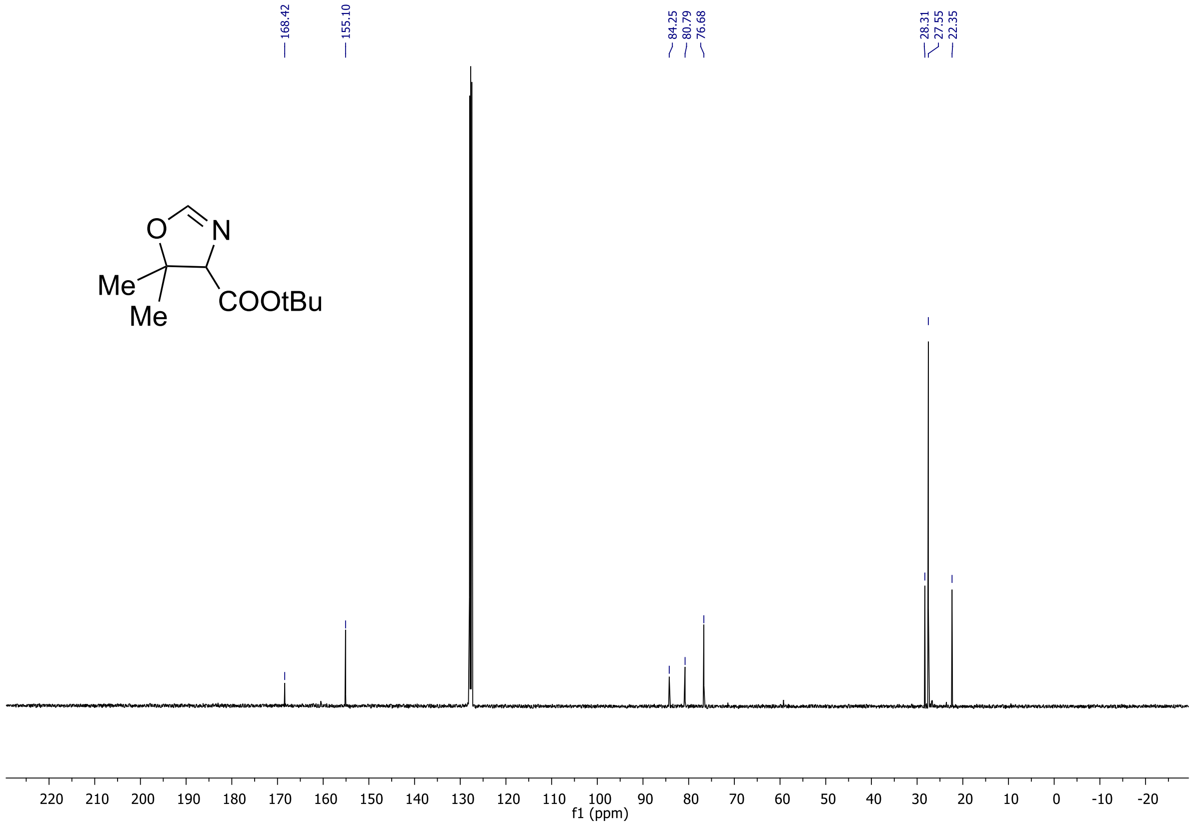

HPLC traces of racemic product

Current Chromatogram(s)

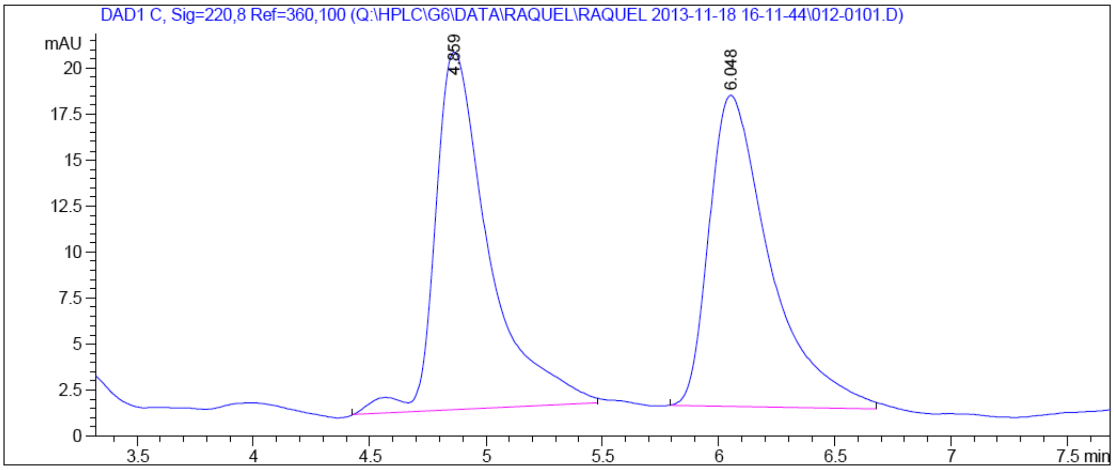

Peak #	RetTime [min]	Type	Width [min]	Area [mAU*s]	Height [mAU]	Area %
1	4.859	BB	0.2372	312.04694	19.45072	50.4168
2	6.048	BB	0.2698	306.88721	16.90664	49.5832

Totals : 618.93414 36.35736

HPLC traces of **4y**

Current Chromatogram(s)

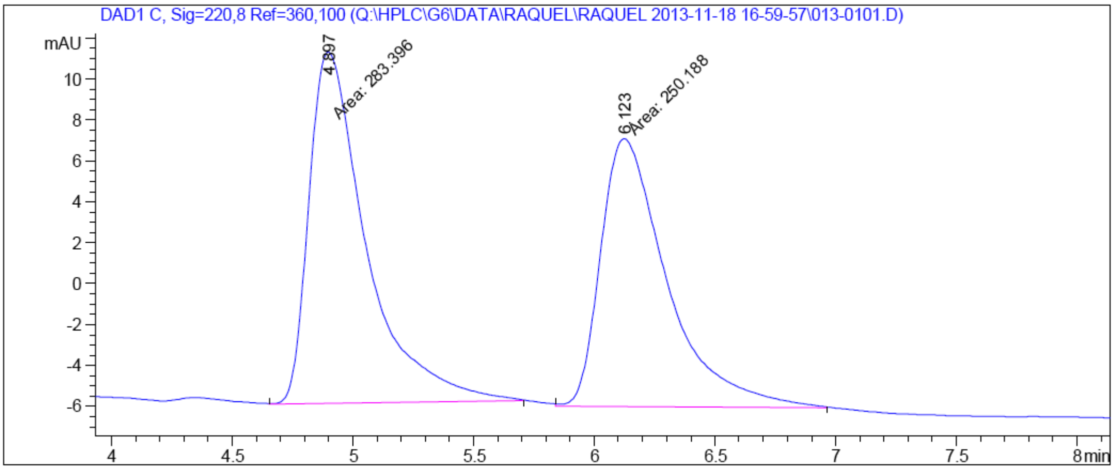

Signal 3: DAD1 C, Sig=220,8 Ref=360,100

Peak #	RetTime [min]	Type	Width [min]	Area [mAU*s]	Height [mAU]	Area %
1	4.897	MM	0.2748	283.39566	17.18750	53.1117
2	6.123	MM	0.3188	250.18811	13.08142	46.8883

Totals : 533.58377 30.26892

6.6 ^{1}H and ^{13}C-NMR spectra and HPLC traces of compound 4z

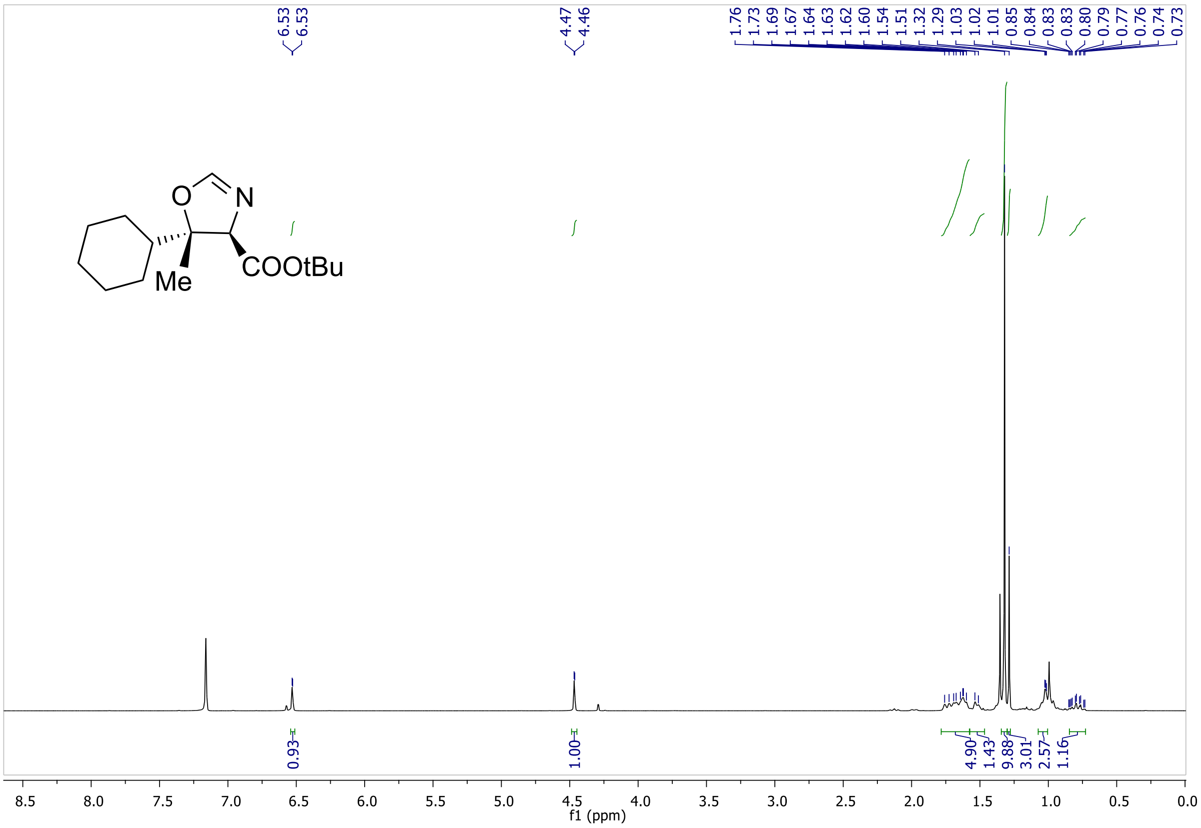

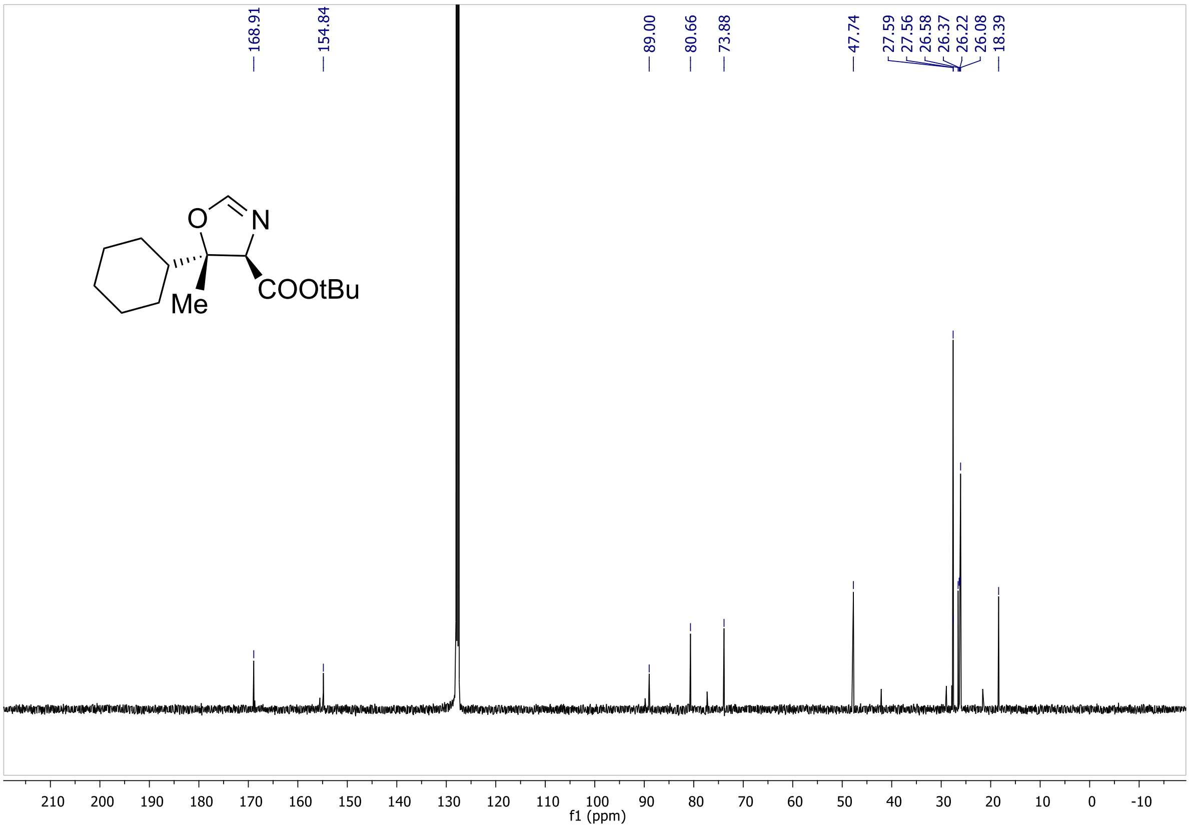

HPLC traces of **4z**

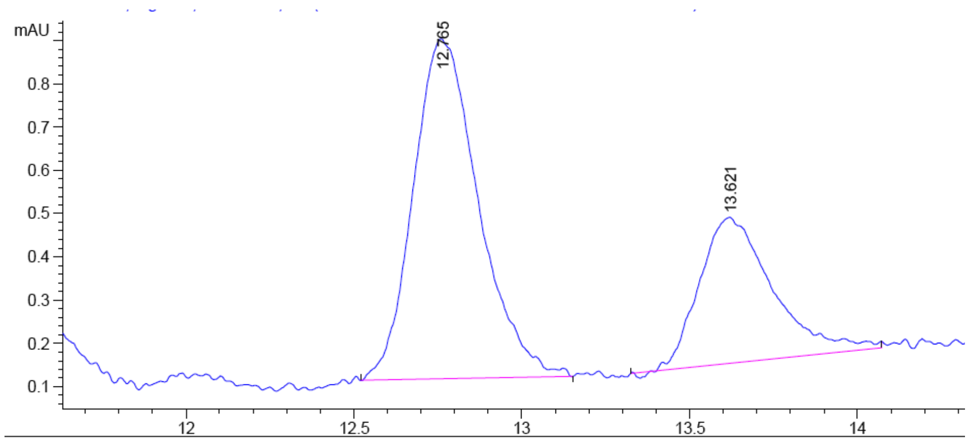

Peak #	RetTime [min]	Type	Width [min]	Area [mAU*s]	Height [mAU]	Area %
1	12.765	BB	0.2106	10.62484	7.81774e-1	67.4861
2	13.621	BB	0.2290	5.11890	3.37373e-1	32.5139

HPLC traces of the enantiomer of **4z**

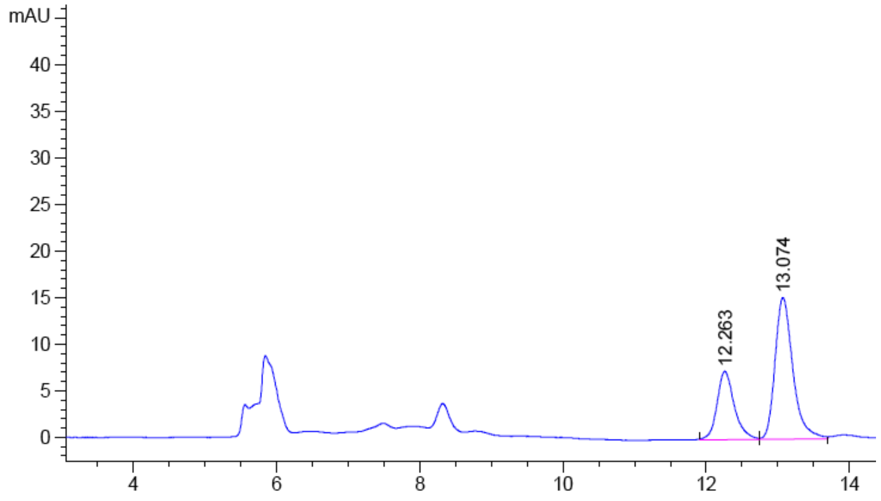

Peak #	RetTime [min]	Type	Width [min]	Area [mAU*s]	Height [mAU]	Area %
1	12.263	BV	0.2563	123.37828	7.33200	31.8211
2	13.074	VV	0.2633	264.34637	15.17248	68.1789

HPLC traces of mixture of both enantiomers

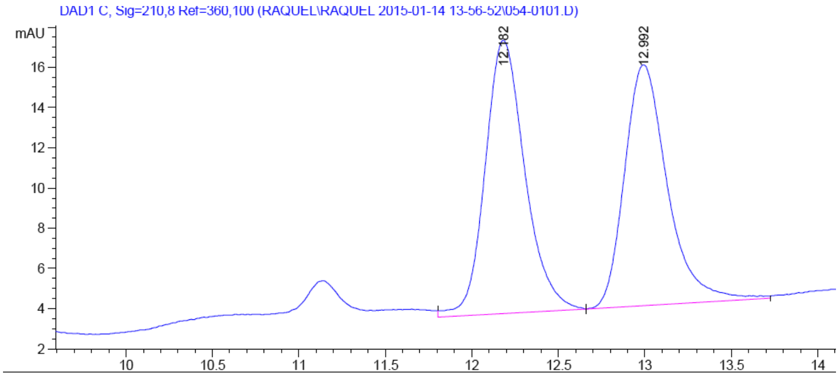

Peak #	RetTime [min]	Type	Width [min]	Area [mAU*s]	Height [mAU]	Area %
1	12.182	VV	0.2469	217.25031	13.56342	51.2339
2	12.992	VB	0.2658	206.78554	11.95432	48.7661
